# Analysis of vitamin D receptor binding affinities of enzymatically synthesized triterpenes including ambrein and unnatural onoceroids

**DOI:** 10.1038/s41598-024-52013-7

**Published:** 2024-01-16

**Authors:** Daijiro Ueda, Natsu Matsuda, Yuka Takaba, Nami Hirai, Mao Inoue, Taichi Kameya, Tohru Abe, Nao Tagaya, Yasuhiro Isogai, Yoshito Kakihara, Florian Bartels, Mathias Christmann, Tetsuro Shinada, Kaori Yasuda, Tsutomu Sato

**Affiliations:** 1 Graduate School of Science and Technology, Niigata University, Niigata, Japan; 2 Department of Pharmaceutical Engineering, Faculty of Engineering, Toyama Prefectural University, Imizu, Japan; 3 Graduate School of Medical and Dental Sciences, Niigata University, Niigata, Japan; 4 Institute of Chemistry and Biochemistry, Freie Unversität Berlin, Berlin, Germany; 5 Graduate School of Science, Osaka Metropolitan University, Osaka, Japan

**Keywords:** Biochemistry, Chemical biology

## Abstract

Onoceroids are a rare family of triterpenes. One representative onoceroid is ambrein, which is the main component of ambergris used as a traditional medicine. We have previously identified the onoceroid synthase, BmeTC, in *Bacillus megaterium* and succeeded in creating ambrein synthase by introducing mutations into BmeTC. Owing to the structural similarity of ambrein to vitamin D, a molecule with diverse biological activities, we hypothesized that some of the activities of ambergris may be induced by the binding of ambrein to the vitamin D receptor (VDR). We demonstrated the VDR binding ability of ambrein. By comparing the structure–activity relationships of triterpenes with both the VDR affinity and osteoclastic differentiation-promoting activity, we observed that the activity of ambrein was not induced via the VDR. Therefore, some of the activities of ambergris, but not all, can be attributed to its VDR interaction. Additionally, six unnatural onoceroids were synthesized using the BmeTC reactions, and these compounds exhibited higher VDR affinity than that of ambrein. Enzymatic syntheses of onoceroid libraries will be valuable in creating a variety of bioactive compounds beyond ambergris.

## Introduction

**Figure 1:**
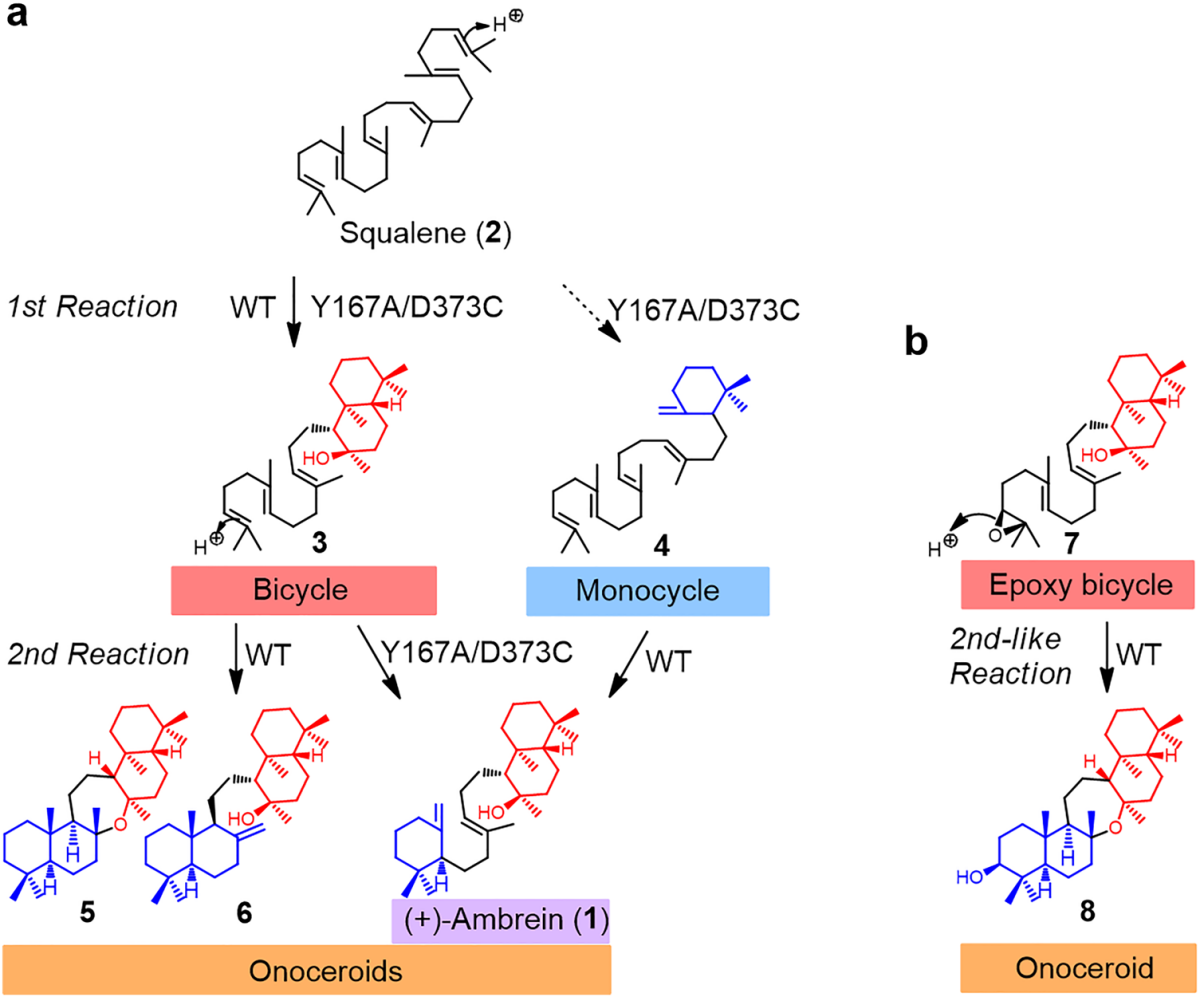
Synthetic pathway of onoceroids reported previously. (**a**) Conversion pathway of **2** into onoceroids catalyzed by BmeTC^WT^ and BmeTC^Y167A/D373C^. The major and minor pathways are indicated by solid and dashed arrows, respectively. (**b**) Conversion pathway of **7** into an onoceroid catalyzed by BmeTC^WT^.

Onoceroids are a rare family of triterpenes that are biosynthesized by cyclizing from both termini of squalene (**2**) and its derivatives (mono- and dioxidosqualene). Owing to this unique biosynthesis mechanism, most onoceroids harbor non-fused ring structures at both ends, such as the ambrane skeleton (6 + 6/6) of **1** and onocerane skeleton (6/6 + 6/6) of **6** (Fig. 1a). Nevertheless, only few studies describing the discovery of onoceroids as natural products as wells as their biological activities are available^1–4^. One of the representative onoceroids is ambrein (**1**; Fig. 1a), the main component of ambergris produced by sperm whales^1,5,6^. Ambergris is used in the perfume industry and traditional medicine^1,5,6^. The photo-oxidative decomposition of **1** converts it into aromatic components while also exhibiting various biological activities^1,5,6^. We have previously identified the first onoceroid synthase (BmeTC) in *Bacillus megaterium* and reported that one enzyme catalyzes the stepwise cyclization of **2** from both ends (Fig. 1a; **2** → **3** → **5** + **6**)^1^. The BmeTC reaction using unnatural triterpene **4,** which is produced by a mutant squalene-hopene cyclase (SHC), as a substrate successfully formed **1** (Fig. 1a; **4** → **1**)^1^. Furthermore, a BmeTC variant (Y167A/D373C) was developed as an ambrein (**1**) synthase that efficiently produces **1** as the main product (Fig. 1a; main pathway: **2** → **3** → **1**)^5^. In addition, we reported that an intermediate **8** for the synthesis of cupacinoxepin, a natural onoceroid isolated from *Cupania cinerea*, can be synthesized from the BmeTC reaction with substrate **7**, an epoxy form of **3** (Fig. 1b; **7** → **8**)^7^.

**Figure 2:**
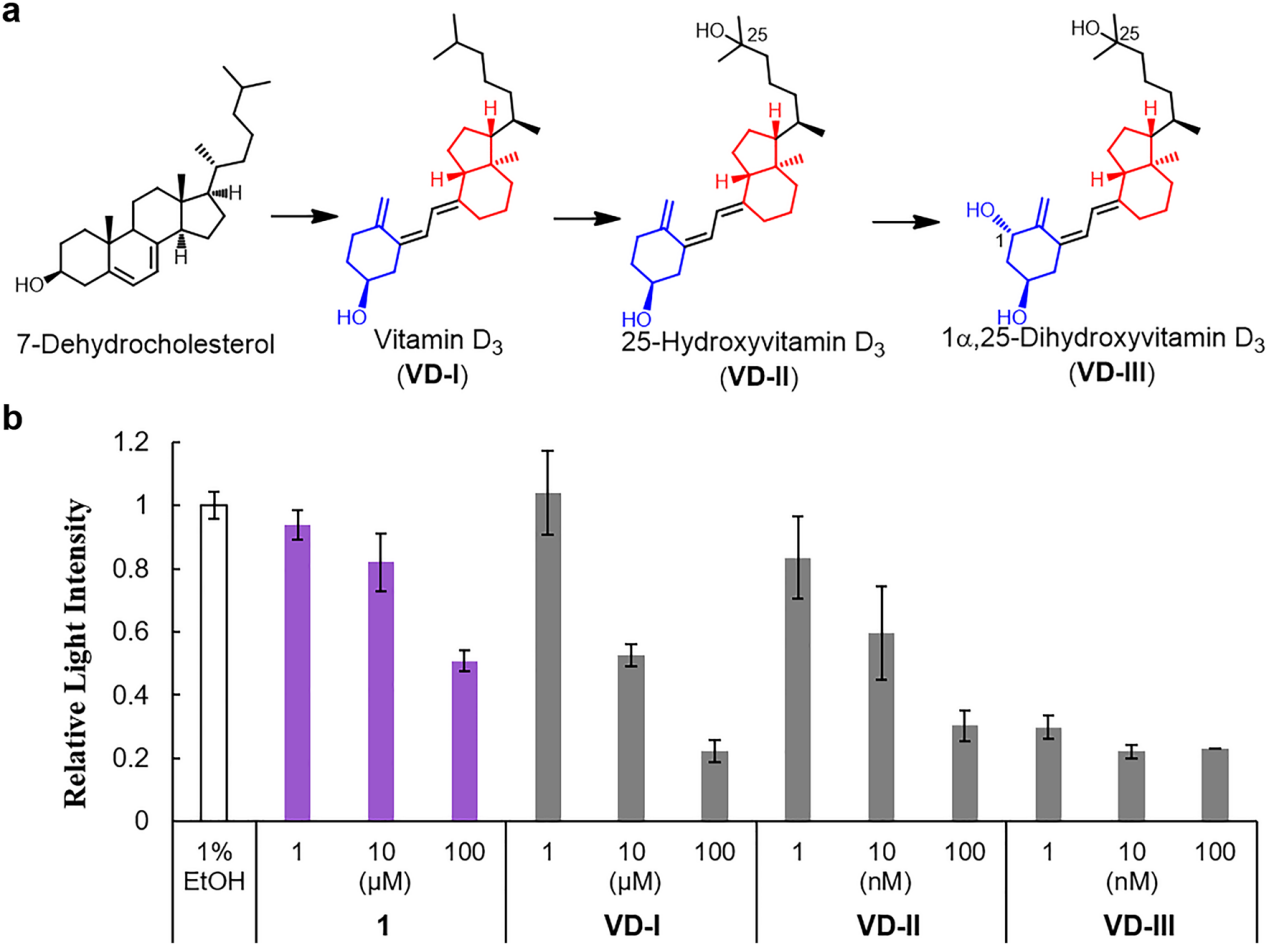
VDR binding affinity of **1**. (**a**) Biosynthesis and metabolic pathway of vitamin D_3_. (**b**) Comparison of binding affinities of **1** and **VD-I-III** to the ligand binding domain of the human VDR. Relative light intensity, compared to that of the control (1% ethanol), is shown. Data are represented as the mean ± SD of at least four independent determinations.

The vitamin D family also has non-fused mono- and bicyclic structures similar to those of onoceroids (especially **1**) (Fig. 2a)^8–10^. However, vitamin D is not an onoceroid, as it is known to be biosynthesized from sterol (ex. 7-dehydrocholesterol), which has a fused tetracyclic structure at one end (Fig. 2a)^8–10^. Vitamin D_3_ (**VD-I**) biosynthesized in animals is metabolized to 25-hydroxyvitamin D_3_ (**VD-II**) in the liver, and further metabolized in the kidney into the active form of vitamin D_3,_ 1α, 25-dihydroxy vitamin D_3_ (**VD-III**) (Fig. 2a)^8–10^. **VD-III** strongly binds to the vitamin D receptor (VDR) to promote various physiological activities such as bone formation, cell proliferation, and cell differentiation. In this study, we focused on the structural similarities between **1** and members of the vitamin D family and gained insight into various biological effects of ambergris and **1**. Furthermore, to advance the development of drugs with a higher biological activity than that of ambergris, we identified the compounds with a higher VDR affinity than that of **1** among the onoceroids synthesized using BmeTC.

## Results

### VDR binding ability of **1**

Ambergris is used not only as a fragrance but also as a traditional medicine with a wide range of biological activities^1,5,6^. As the vitamin D_3_ group (**VD-I-III**) with non-fused mono- and bicyclic structures is molecularly similar to **1** (Fig. 2a) and also known to have various biological effects^8–10^, we hypothesized that some of the activities of ambergris and **1** may be mediated by the vitamin D receptor (VDR). Therefore, we analyzed the VDR binding affinity of **1**. We observed that **1** bound to the VDR at concentrations that could exhibit our previously reported biological effects (promoting osteoclast differentiation and inhibiting amyloid-β-induced neuronal cell death)^7^, although the binding affinity of **1** exhibited a much lower EC_50_ value (112 ± 29 μM for **1** versus 0.00064 ± 0.00022 μM for **VD-III**) than the active form of vitamin D (Fig. 2b, Table S1). Therefore, it is possible that some of the diverse biological effects of ambergris and **1** are mediated via the VDR.

### Structure–activity relationships of triterpenes for VDR affinities

**Figure 3:**
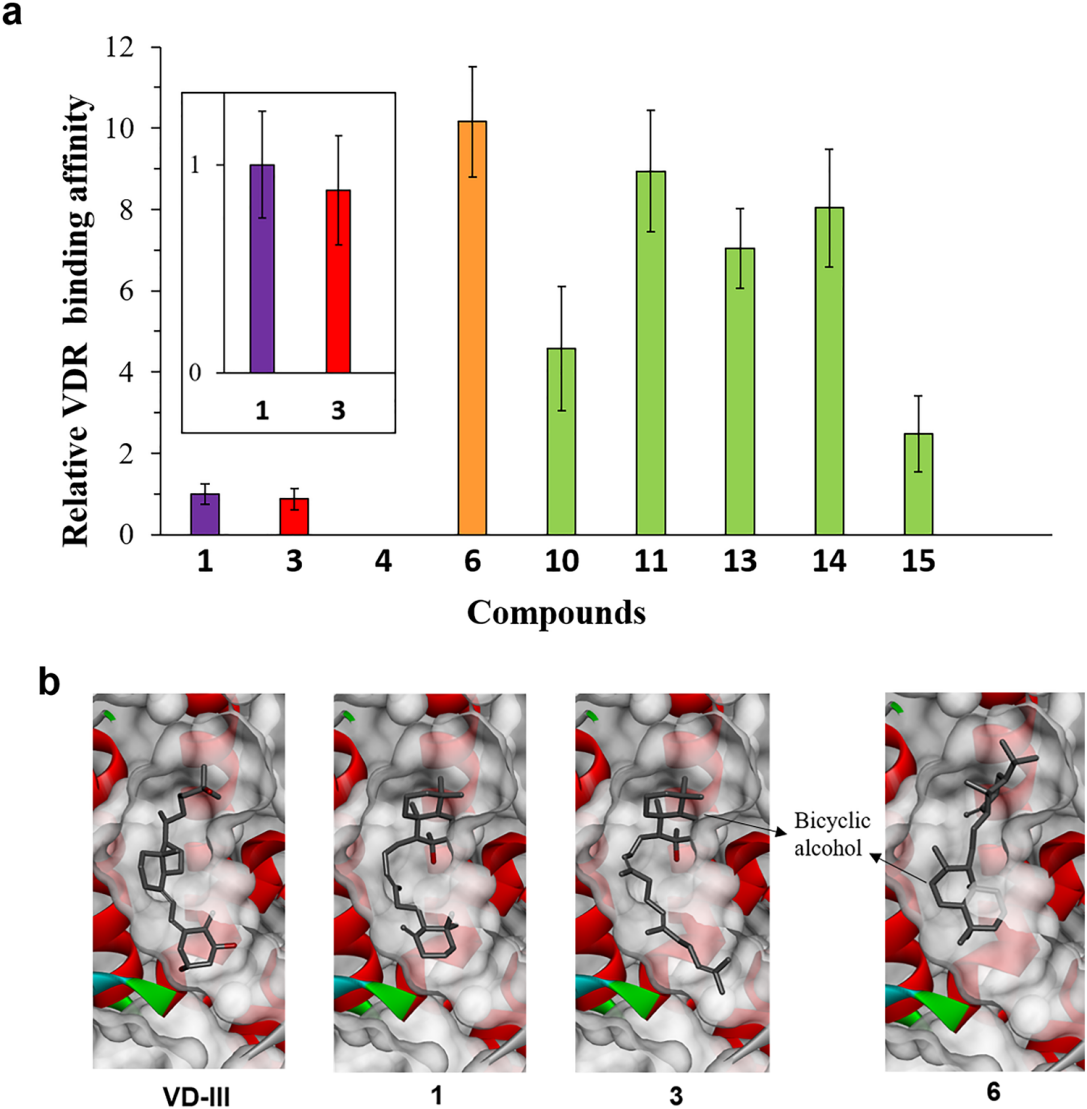
Interaction between triterpenes and the VDR. (**a**) The relative VDR binding affinity of each compound. Each value was determined based on the concentration at which the luminescence reached 50% of the maximum value and normalized to that of **1**. Data are represented as the mean ± SD of at least triplicate determinations. A graph showing only the affinities of **1** and **3** is also included. (**b**) Comparison of the complex structures of the human VDR with **VD-III**, **1**, **3**, and **6**.

Using previously isolated intermediates and final products (**3**, **4**, and **6**) produced by BmeTCs, we analyzed the structure–activity relationship relevant for **1** to bind to the VDR. The binding affinities of **4** and **3** (Fig. 1a), which have only one of the monocyclic or bicyclic skeletons of **1**, were compared; **3** had similar binding affinities to **1**, whereas **4** did not bind to the VDR (Fig. 3a, Table S1). These results suggest that the bicyclic structure of **1** is important for binding to the VDR. Our docking models with the VDR suggest that the monocyclic and bicyclic orientations of both **VD-III** and **1** were consistent (Fig. 3b). The affinity of **6** was the highest and was tenfold higher than that of **1** (Fig. 3a). The docking model of the VDR with **6** suggests that the binding mode of **6** may be largely different from that of **VD-III**, **1**, and **3** (Fig. 3b). As the calculated binding energies of compounds in docking simulations (**VD-III** < **6** < **1**; Table S2) were consistent with the experimental results of their VDR affinities (**VD-III** > **6** > **1**; Fig. 3a; Table S1), the model combining **6** would be reasonable. These results indicate that using other onoceroids, which also have a different binding mode compared to that of **VD-III**, it is possible to develop a compound with a higher affinity for the VDR than **1**.

### Structure–activity relationships of triterpenes for osteoclastic differentiation-promoting activities

**Figure 4:**
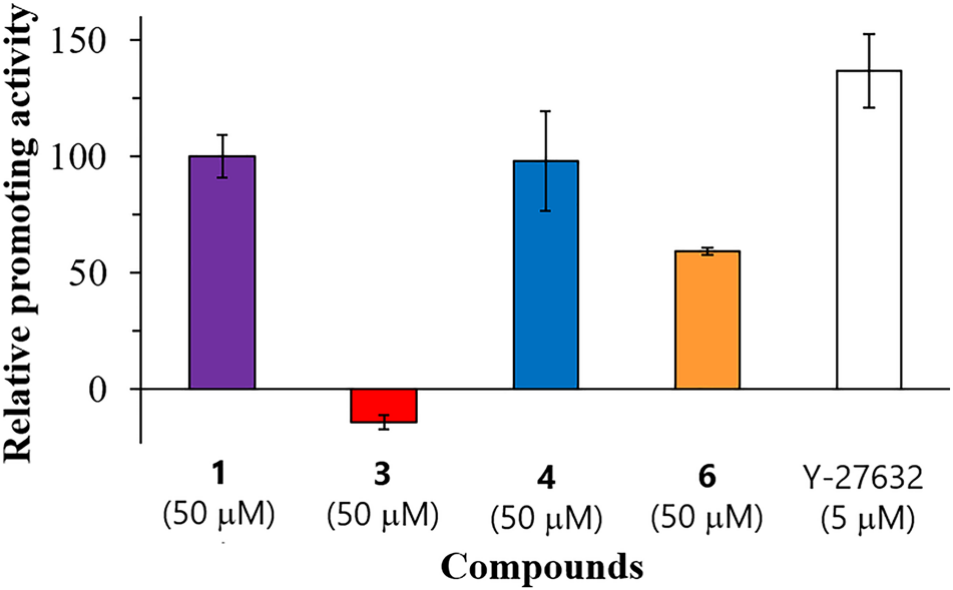
Osteoclastic differentiation-promoting activity of triterpenes. Relative promoting activity is a value relative to the number of osteoclasts increased by the addition of 50 μM **1**. Y-27632^22^ is a positive control. Data are expressed as mean ± SD (n = 3).

**VD-III** promotes the differentiation of osteoclast precursor RAW264.7 cells into osteoclasts and that VDR is expressed in RAW264.7 cells^11,12^. Therefore, the positive regulation of osteoclastic differentiation by **VD-III** could be mediated via the VDR in osteoclast precursor cells. Furthermore, we previously reported that **1** promoted osteoclast differentiation^5^. In this study, by comparing the structure–activity relationship between the osteoclastic differentiation-promoting activity and the VDR binding affinity, we evaluated whether **1** exhibited its activity via the VDR. The monocyclic **4** showed similar osteoclastic differentiation-promoting activity as **1**, while the bicyclic **3** showed no activity, (Fig. 4) despite **1** and **3** exhibiting higher VDR binding affinities (Fig. 3a). In addition, the activity of **6**, whose VDR binding affinity was tenfold higher than that of **1** (Fig. 3a), was not comparable to that of **1** (Fig. 4). Therefore, the structure–activity relationship between the two analyses was significantly different, thus, suggesting that the osteoclastic differentiation-promoting activity of **1** may not be mediated via the VDR. A similar approach can be adopted to estimate whether other biological activities of **1** are mediated via the VDR. In addition, it has become clear that the essential structure for exhibiting **1**-like biological activity differs depending on the type of biological activity.

### Enzymatic synthesis of unnatural onoceroids

**Figure 5:**
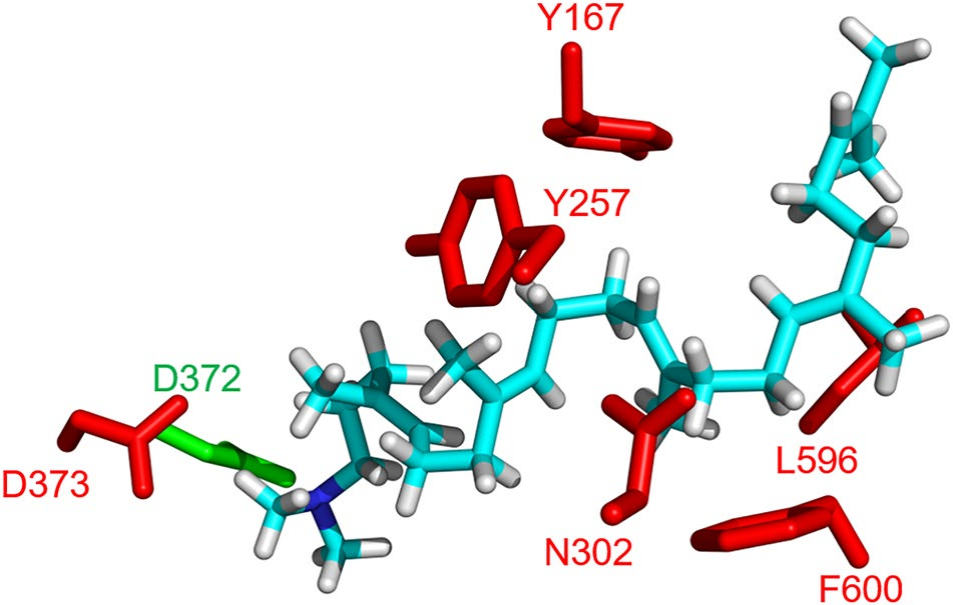
Homology model of the BmeTC. The 3D structure of BmeTC was modeled using AlphaFold2. The structure was constructed using the coordinates of SHC complexed with 2-azasqualene inhibitor [Protein Data Bank (PDB) code: 1UMP] as a template and visualized using PYMOL. Blue: 2-azasqualene; red: residues targeted in this study; green: initiation site.

**Figure 6:**
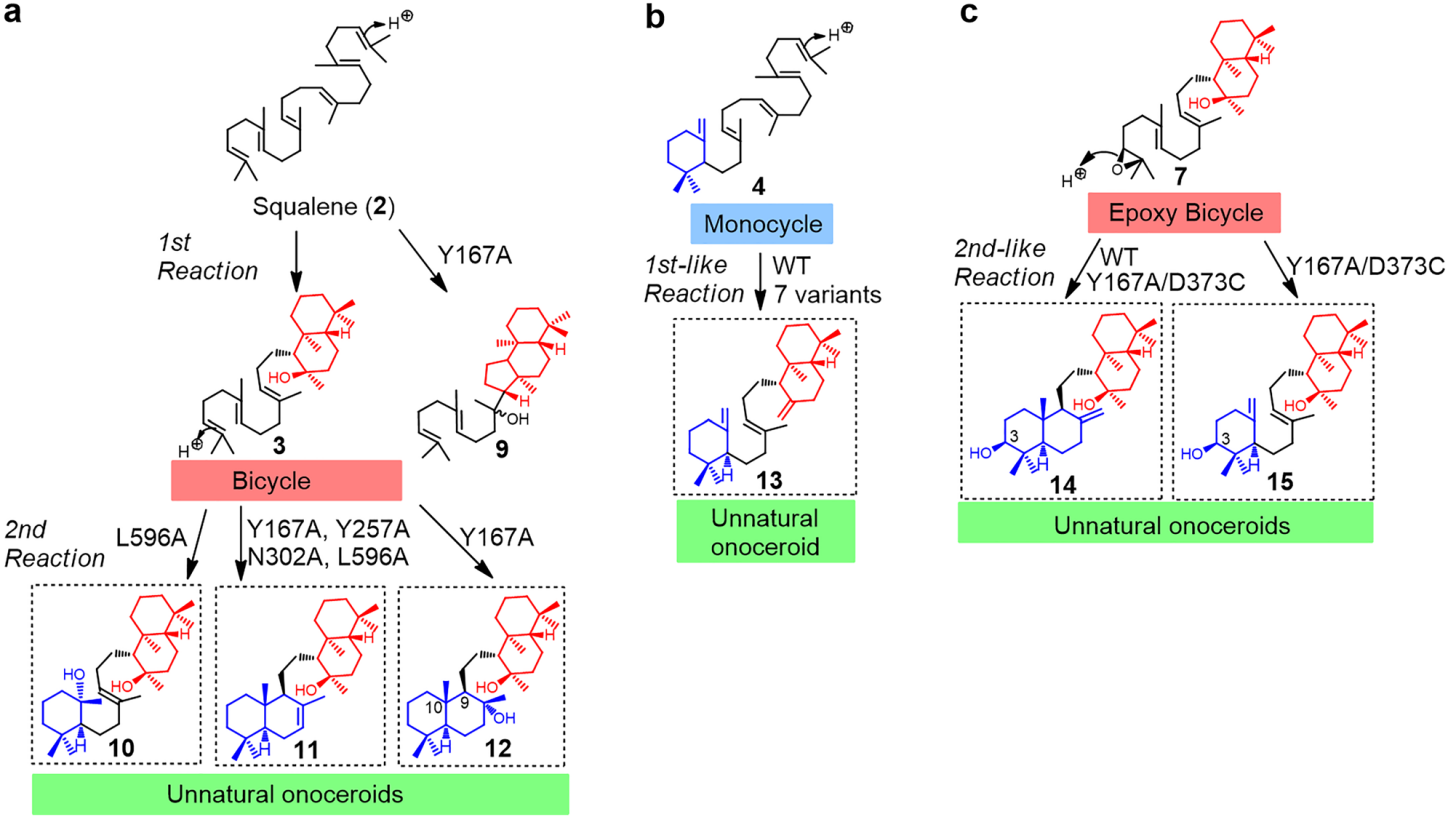
Pathway of onoceroid synthesis discovered in this study. The unnatural onoceroids isolated for the first time in this study are indicated in the dashed boxes.

Because **6** exhibits a higher VDR affinity than **1**, we planned to search for high-VDR-affinity compounds among the enzymatically synthesized unnatural onoceroids. We previously detected abnormal products of the BmeTC variants during the development of ambrein synthase (substituted residues are shown in Fig. 5). In this study, six novel products (**10**–**15**) were isolated from the reaction of wild-type or variant BmeTCs with substrates **2**, **4**, or **7**, and their structures were determined using mass spectrometry (MS) and nuclear magnetic resonance (NMR) (Figs. 6, S1–S52). All compounds (**10**–**15**) were unnatural onoceroids. Compared to **1**, compounds **10**, **13**, and **15** had ambrane skeletons and differed in mono-, bi-, and monocyclic structures, respectively (Fig. 6). Alternatively, **11**, **12**, and **14** had onocerane skeletons, and all differed in their bicyclic structure from that of the natural onoceroid **6** (blue in Fig. 6). Although **12** had the same planar structure as the natural product isolated from *Aristolochia giberti*, the stereochemistry at positions 9 and 10 of **12** was opposite to that of the natural product (Fig. 6a)^13^.

When **2** was used as a substrate, BmeTC variants produced **10**, **11**, and **12** as minor products along with the normal products **3**, **5**, and **6** (Fig. 6a, Table 1). As **10**–**12** were also produced from substrate **3**, **10**–**12** could be synthesized from substrate **2** through two steps using **3** as an intermediate (Figs. 6a, S53). During the conversion of **3** to **10** by the BmeTC^L596A^ (Fig. 6a), the reaction was terminated at the monocyclic stage through water addition rather than via a double bond attack to the carbocation for bicyclic formation. This suggests that the L596 residue prevents water entry during the second step of the bicyclic formation. The BmeTC^Y167A^ has already been shown to produce tricyclic **9** as an abnormal product from substrate **2** (Fig. 6a, Table 1)^14^. This indicates that the Y167 residue may be located near the C-ring within the active site and functions to stop the reaction in the first bicyclic step^14^. The present study isolated unidentified products (**11** and **12**) of the BmeTC^Y167A^ that are less abundant than **9** (Fig. 6, Table 1). These compounds (**11** and **12**) would be formed in the second step of cyclization with **3** as an intermediate (Fig. 6), thus, suggesting that the Y167 residue would be involved in the termination of both the first- and second-step reaction. The other three variants (Y257A, N302A, and L596A) also yielded **11** (Table 1), indicating that Y257, N302, and L596 residues are also involved in the termination of the second step.

**Table 1:** Ratio of products formed from substrate **2**.

	3	5	6	9	10	11	12	A^a^
WT	45.1	36.5	18.4	–	–	–	–	–
Y167A	1.6	20.8	41.9	21.6	–	4.8	3.0	6.3
Y257A	–	17.5	57.4	–	–	9.1	–	16.0
N302A	13.4	10.1	67.1	–	–	1.9	–	7.5
L596A	7.2	31.6	52.5	–	6.6	2.1	–	–
F600A	65.5	12.3	13.3	–	–	–	–	8.9

^a^Because **A** could not be isolated, its structure could not be determined.

Although BmeTC^WT^ was reported to synthesize **1** from substrate **4** (Fig. 1a, Table 2)^1^, the structure of its minor product **13** remained unknown. In this study, **13** was isolated from the reaction of BmeTC^I594G^, which produces more **13** than the wild type or other variants. Furthermore, we showed that **13** is an unnatural onoceroid with the ambrane skeleton (Fig. 6). In addition, we discovered that the production ratio of **13** in BmeTC^L596A^ was extremely low (Table 2). Among the wild type and all variants of BmeTCs, BmeTC^L596A^ yielded the maximum concentration of **1** using **4** as the substrate^5^. Therefore, it was suggested that BmeTC^L596A^ would have an active site shape in which water addition is preferred over deprotonation following the occurrence of bicyclic carbocation in the reaction of substrate **4**.

**Table 2:** Ratio of products formed from substrate **4**.

	1	13	B^a^
WT	56.3	43.7	–
Y167A	90.0	10.0	–
Y257A	45.3	54.7	–
N302A	33.4	52.5	14.1
I594G	29.3	70.7	–
L596A	92.0	8.0	–
F600A	31.5	40.4	28.1

^a^As **B** could not be isolated, its structure could not be determined.

We had previously reported the formation of **8** from substrate **7** in the reaction of BmeTC^WT^ (Fig. 1b)^7^. However, the structure of **14**, a minor product, remained unsolved (Table 3). Because a new product **15** was produced in addition to **8** and **14** by the ambrein synthase (Y167A/D373C) (Table 3), **14** and **15** were isolated from the enzymatic reaction products of the ambrein synthase. In addition, the ambrein synthase (Y167A/D373C) was shown to form an ambrane backbone from substrate **7** similar to that of **3**. Lastly, it has been shown that a cyclic structure with a β-hydroxy at the 3-position can be formed from a substrate with an *S*-epoxy at the end by wild type and variant BmeTCs, which is similar to the reaction of the SHC^15^.

**Table 3:** Ratio of products formed from substrate **7**.

	8	14	15
WT	53.9	46.1	–
Y167A/D373C	17.8	20.4	61.8

### VDR binding affinities of unnatural onoceroids

The five newly isolated unnatural onoceroids (**10**, **11**, **13**–**15**) were also analyzed for their VDR binding affinities. All onoceroids exhibited more potent VDR binding affinities than that of **1** (Fig. 3a, Table S1). The onoceroids **11**, **13**, and **14** had higher affinity than **10** and **15**, suggesting that, similar to **6**, the bicyclic structure with a double bond is important for binding to the VDR. In addition, **13** showed a higher affinity than **1** and **4**, strongly supporting the importance of the bicyclic structure with a double bond for the VDR binding. As shown in the VDR structure following **6** binding (Fig. 3b), the bicyclic structure with a double bond probably has a higher affinity for the recognition site of the bicyclic and side chain of **VD-III** than the bicyclic structure with a hydroxyl group. As there are no amino acid residues that form hydrogen bonds with the hydroxyl groups of **1** and **6** in the VDR structure, the bicyclic carbon skeleton may be more important for VDR binding than the hydroxyl groups. The onoceroids synthesized in this study may be used in the future to develop useful substances that exhibit biological activity via the VDR. As we were able to develop ambrein (**1**) synthase by mutating BmeTC^5^, we may also be able to create enzymes that efficiently synthesize other onoceroids such as **6**, **11**, **13**, and **14**.

## Discussion

In this study, we showed that some biological effects associated with ambergris may be induced by **1** binding to the VDR. Furthermore, we succeeded in enzymatically synthesizing unnatural onoceroids that exhibit more potent VDR affinity than that of the natural product **1**, which may lead to the development of drugs with superior biological activity compared to that of ambergris. By contrast, we discovered that the VDR was not involved in the osteoclastic differentiation promoting effects of **1**, indicating that the biological activities of ambergris and **1** have diverse sites of action. Since the important structures of **1** differ depending on the type of biological activity, expanding the diversity of the onoceroid library through enzymatic synthesis will become increasingly valuable in the future.

The present study also points to a new direction in triterpene cyclase research. Following analysis of the variant triterpene cyclases based on their three-dimensional structure, the structures of abnormal products have been used to understand the catalytic mechanism of the target residues^16,17^. Furthermore, the use of unnatural substrates has led to the synthesis of natural products^7^. To the best of our knowledge, this is the first study that used variant enzymes or unnatural substrates to produce unnatural triterpenes and explored their biological activity. Considering that **1** could be efficiently synthesized^5^, it will be possible to create enzymes suitable for producing unnatural triterpenes. Hence, industrial biosynthesis of biologically active unnatural triterpenes that are useful to humans is attainable in the future.

## Methods

### General procedure and materials

NMR spectra were acquired using a Bruker DMX 600 spectrometer at 600 MHz for proton and 125 MHz for carbon. Gas chromatography-mass spectrometry (GC–MS) analyses (injection temperature: 300 °C; oven temperature: 220–300 °C at an increment of 1 °C/min^−1^) were conducted on a JMST100GCV spectrometer (JEOL, Tokyo, Japan) fitted with a DB1 capillary column (30 m × 0.25 mm × 0.25 μm; J&W Scientific, Inc., Folsom, CA, USA), using the EI mode at 70 eV. HRMS was performed on a JMST100LP spectrometer (JEOL) using ESI mode. GC analyses (injection temperature: 300 °C; oven temperature: 220–300 °C at an increment of 3 °C min^−1^) were carried out on a Shimadzu GC2014 chromatograph equipped with a flame ionization detector (30 m × 0.32 mm × 0.25 μm; J&W Scientific, Inc.). The specific rotation was measured using a Horiba SEPA300 polarimeter. *Escherichia coli JM109* was used for sequencing analysis. Compound **2** was procured from Kanto Kagaku. Products **3**, **4**, and **6** were isolated in previous studies^1,5,18^. Cell-free extracts from *E. coli* BL21(DE3) harboring pColdTF-BmeTC^X^ were prepared as previously reported^5^. All enzymatically synthesized triterpenes were confirmed to have a purity of 99% or higher using HPLC and used in the analysis of the VDR binding affinities and osteoclastic differentiation-promoting activities. **VD-I** was purchased from Wako Pure Chemicals (Osaka, Japan). **VD-II** and **VD-III** were purchased from Cayman Chemical Company (Ann Arbor, MI, USA).

### Isolation and structural determination of **10**

The method for isolating product **10** is similar to that previously described^5^ and is summarized in Fig. S1. To isolate product **10** formed using BmeTC^L596A^, **2** (324 mg) was emulsified with Tween 80 (6.5 g) in buffer A (162 mL) and incubated with cell-free extract (648 mL) containing 0.1% ascorbic acid at 30 °C for 64 h. Subsequently, 15% KOH/MeOH solution (1215 mL) was added to the reaction mixture and lipophilic products were extracted with *n*-hexane (1 L × 3) and concentrated. The *n*-hexane extract (278.2 mg) was partially purified using silica gel (15 g) column chromatography with *n*-hexane and *n*-hexane/EtOAc (100:20). The fraction (Fra. A: 104.5 mg) eluted with *n*-hexane contained substrates **2**, whereas the fraction (Fra. B: 134.1 mg) eluted with *n*-hexane/EtOAc (100:20) contained **2**, **6**, and **10**. Pure product **10** (oil; 6.9 mg) was obtained using HPLC (Packed Column Capcell Pak C18, 4.6 × 150 mm; SHISEIDO) with acetonitrile from Fra. B.

The structure of product **10** was determined using MS (Fig. S2) and NMR spectroscopy (Figs. S3–S9). [a]^25^_D_ = − 26.064 (*c* = 0.09 in EtOH); HRMS (ESI): *m*/*z*: calcd for C_30_H_54_NaO_2_ (**10**): 469.4016 [M+Na]^+^, found: 469.4023.

### Isolation and structural determination of **11** and **12**

The method for isolating **11** and **12** is similar to that previously described^5^ and is summarized in Fig. S10. To isolate products **11** and **12** formed using the BmeTC^Y167A^, 350 mg of **2** was emulsified with TritonX-100 (7.0 g) in buffer A (162 mL), and incubated with cell-free extract (500 mL) containing 0.1% ascorbic acid at 30 °C for 64 h. Subsequently, 15% KOH/MeOH solution (750 mL) was added to the reaction mixture and lipophilic products were extracted with *n*-hexane (1.41 L × 3) and concentrated. The *n*-hexane extract (429.4 mg) was partially purified using silica gel (25 g) column chromatography with *n*-hexane and *n*-hexane/EtOAc (100:20). The fraction (98.7 mg) eluted with *n*-hexane/EtOAc (100:20) contained **9**, **11**, and **12**. This fraction was partially purified using silica gel (2 g) column chromatography with *n*-hexane, *n*-hexane/EtOAc (100:1), *n*-hexane/EtOAc (100:5), and *n*-hexane/EtOAc (100:20). The fraction (Fra. A: 69.7 mg) eluted with *n*-hexane/EtOAc (100:1) contained product **11** whereas the fraction (Fra. B: 8.8 mg) eluted with *n*-hexane/EtOAc (100:20) contained product **12**. Pure product **11** (oil; 0.7 mg) was obtained using the SiO_2_ HPLC (Inertsil 100A, 7.6 × 250 mm; GL Science, Tokyo, Japan) with *n*-hexane/THF (100:2) from Fra. A, and pure **12** (oil; 1.25 mg) were obtained using the SiO_2_ HPLC (Inertsil 100A, 7.6 × 250 mm; GL Science) with *n*-hexane:2-propanol (100:2) from Fra. B.

The structure of **11** was determined using MS (Fig. S11) and NMR spectroscopy (Figs. S12–S18). [α]^25^_D_ = − 38.41 (*c* = 0.07 in EtOH); HRMS (ESI): *m*/*z*: calcd for C_30_H_52_NaO (**11**): 451.3910 [M+Na]^+^, found: 451.3906. The structure of **12** was determined using MS (Fig. S19) and NMR spectroscopy (Figs. S20–S26). [α]^25^_D_ = − 122.80 (*c* = 0.12 in EtOH); HRMS (ESI): *m*/*z*: calcd for C_30_H_54_NaO_2_ (**12**): 469.4016 [M+Na]^+^, found: 469.4010.

### Isolation and structural determination of **13**

The method for isolating **13** is similar to that previously described^5^ and is summarized in Fig. S27. To isolate product **13** formed using the BmeTC^I594G^, 13 mg of **4** was emulsified with TritonX-100 (260 mg) in buffer A (130 mL) and incubated with cell-free extract (520 mL) containing 0.1% ascorbic acid at 30 °C for 112 h. Subsequently, 15% KOH/MeOH solution (780 mL) was added to the reaction mixture and lipophilic products were extracted with *n*-hexane (1.43 L × 3) and concentrated. The *n*-hexane extract (2.35 g) was partially purified using silica gel (120 g) column chromatography with *n*-hexane and *n*-hexane/EtOAc (100:20). The fraction (13.0 mg) eluted with *n*-hexane contained **4** and **13**. Pure **13** (oil; 0.32 mg) was obtained using the SiO_2_ HPLC (Inertsil 100A, 7.6 × 250 mm; GL Science) with *n*-hexane from Fra. A.

The structure of **13** was determined using MS (Fig. S28) and NMR spectroscopy (Figs. S29–S35). + 24.47 (*c* = 0.05 in CHCl_3_); HRMS (EI): *m*/*z*: calcd for C_30_H_50_ (**13**): 410.3907, found: 410.3918.

### Isolation and structural determination of **14** and **15**

The method for isolating **14** and **15** is similar to that previously described^5^ and is summarized in Fig. S36. To isolate products **14** and **15** formed using the BmeTC^Y167A/D373C^, 10 mg of **12** was emulsified with TritonX-100 (500 mg) in buffer A (100 mL), and incubated with cell-free extract (400 mL) containing 0.1% ascorbic acid at 30 °C for 64 h. Subsequently, 15% KOH/MeOH solution (600 mL) was added to the reaction mixture and lipophilic products were extracted with *n*-hexane (1.1 L × 3) and concentrated. The *n*-hexane extract (215.5 mg) was partially purified using silica gel (10.8 g) column chromatography with *n*-hexane and *n*-hexane/EtOAc (100:20). The fraction (Fra. B:131.9 mg) eluted with *n*-hexane/EtOAc (100:20) contained **14** and **15**. This fraction was partially purified using silica gel (6.5 g) column chromatography with *n*-hexane, *n*-hexane/EtOAc (100:1), *n*-hexane/EtOAc (100:5), and *n*-hexane/EtOAc (100:20). The fraction (Fra. C: 11.7 mg) eluted with *n*-hexane/EtOAc (100:1) contained **14** and **15.** Pure **14** (oil; 4.2 mg 41 min) and pure **15** (oil; 2.4 mg 49 min) were obtained using the SiO_2_ HPLC (Inertsil 100A, 7.6 × 250 mm; GL Science) with *n*-hexane/2-propanol (100:2) from Fra. C.

The structure of **14** was determined using MS (Fig. S37) and NMR spectroscopy (Figs. S38–S44). [α]^25^_D_ = − 166.67 (*c* = 0.05 in EtOH); HRMS (ESI): *m*/*z*: calcd for C_30_H_52_NaO_2_ (**14**): 467.3860 [M+Na]^+^, found: 467.3883. The structure of **15** was determined by MS (Fig. S45) and NMR spectroscopy (Figs. S46–S52). [α]^25^_D_ = − 72.77 (c = 0.10 in EtOH); HRMS (ESI): *m*/*z*: calcd for C_30_H_52_NaO_2_ (**15**): 467.3860 [M+Na]^+^, found: 467.3870.

### Analysis of VDR binding affinity

The binding affinity of each compound for the human VDR was evaluated as described previously^10,19^. In brief, 49 μL of cell lysate derived from recombinant *E. coli* expressing split-luciferase vitamin D biosensor protein was added to individual wells of a 96-well plate. Next, 1 μL of ethanol solution of each compound at appropriate concentrations was added to each well and incubated for 30 min at 25 °C. Subsequently, 50 μL of the Luciferin solution containing 20 mM of MgSO_4_, 2 mM of d-luciferin, and 4 mM of adenosine triphosphate in 25 mM Tris–HCl (pH 7.4) was introduced into each well and incubated for 30 min at 25 °C. The luminescence (photon counts) was measured using a luminometer (Infinite 200 Pro 96-microplate luminometer, Tecan, Switzerland). The relative VDR binding affinity of each compound was determined based on the concentration at which the relative light intensity reached 50% of the maximum value (X). X values were calculated using the following equation:

$$X = {10}^{\left[log\left(\frac{A}{B}\right)\times\frac{50-C}{D-C}+ log \left(B\right)\right]}.$$

In this equation, A and B correspond to concentrations near 50% of the maximum relative light intensity. C and D represent the relative light intensity (%) at concentrations B and A, respectively. Such an analysis for product **12** was not performed as the structure of **12** changed during storage following structural determination.

### Docking simulations with the VDR

The docking simulations were performed based on the X-ray crystallographic structure of the human VDR complexed with **VD-III**, **1**, **3**, and **6** (pdb ID: 1db1)^20^ as follows. To analyze interactions between the VDR and triterpenes, docking models of the apoprotein and ligand were constructed using CDOCKER simulation^21^. For the top-ten docking poses with the best CDOCKER scores, the binding energies were estimated based on the in situ ligand minimization protocol packaged in Discovery Studio 2020, Biovia. The poses with the best binding energies were adopted as the docking models. The binding energy for the native **VD-III** was also obtained by the same procedures as for the triterpene ligands, whereas the model with the native ligand was almost the same as the experimental structure. The binding energies are listed in Table S2.

### Cell culture and osteoclastic differentiation

RAW264.7 cells were used as pre-osteoclastic cells. Cells were cultured in α-MEM medium containing 10% fetal bovine serum, 2 mM l-glutamine, 100 U/mL penicillin, and 100 μg/mL streptomycin. Osteoclastic differentiation was induced following treatment with 100 ng/mL sRANKL (Oriental Yeast, Japan), and each sample was added to the medium and incubated for 4 days with 5% CO_2_ at 37 °C. Cells were fixed in 10% glutaraldehyde for 15 min at 37 °C and subsequently incubated for 10 min at 37 °C in tartrate-resistant acid phosphatase (TRAP) buffer, containing 0.1 M sodium acetate, 0.1 M acetic acid, 10 mg/mL naphthol AS-MX phosphate, 0.1% Triton X-100, 0.3 M potassium tartrate, and 0.3 mg/mL Fast Red Violet LB Salt. TRAP-positive dark-red cells with more than three nuclei were manually counted under a light microscope (20× objective) as multinucleated osteoclasts. Y-27632^22^ was used as a positive control.

### Supplementary Information

Supplementary Information.

## Data Availability

Data supporting the findings of this study are available within the article and the Supplementary Information files as well as from the corresponding author upon reasonable request.
