# Supplementary material for: Analysis of vitamin D receptor binding affinities of enzymatically synthesized triterpenes including ambrein and unnatural onoceroids
Source: Sci Rep. 2024 Jan 16;14:1419. doi: 10.1038/s41598-024-52013-7 (PMC10792010; doi:10.1038/s41598-024-52013-7)
Supplement: Supplementary file 1 — Supplementary Information. [file 41598_2024_52013_MOESM1_ESM.pdf]

Supporting Information

Analysis of vitamin D receptor binding affinities of enzymatically synthesized triterpenes including ambrein and unnatural onoceroids

Daijiro Ueda[1], Natsu Matsuda[1], Yuka Takaba[1], Nami Hirai[1], Mao Inoue[1], Taichi Kameya[1], Tohru Abe[1], Nao Tagaya[2], Yasuhiro Isogai[2], Yoshito Kakihara[3], Florian Bartels[4], Mathias Christmann[4], Tetsuro Shinada[5], Kaori Yasuda[2*], Tsutomu Sato[1**]

Graduate School of Science and Technology, Niigata University,[1] Department of Pharmaceutical Engineering, Toyama Prefectural University ,[2] Graduate School of Medical and Dental Sciences, Niigata University [3] ,Institute of Chemistry and Biochemistry, Freie Unversität Berlin, [4] Graduate School of Science, Osaka Metropolitan University[5]

* kyasuda@pu-toyama.ac.jp, ** satot@agr.niigata-u.ac.jp

Contents

1. Isolation and structural analysis of compounds.	S3-48
2. Gas chromatogram of reaction products and residual substrate.	S49
3. Vitamin D binding affinities of vitamin Ds and triterpenes.	S50
4. Binding energies of native and synthetic ligands in the complex structures of VDR.	S51

1. Isolation and structural analysis of compounds

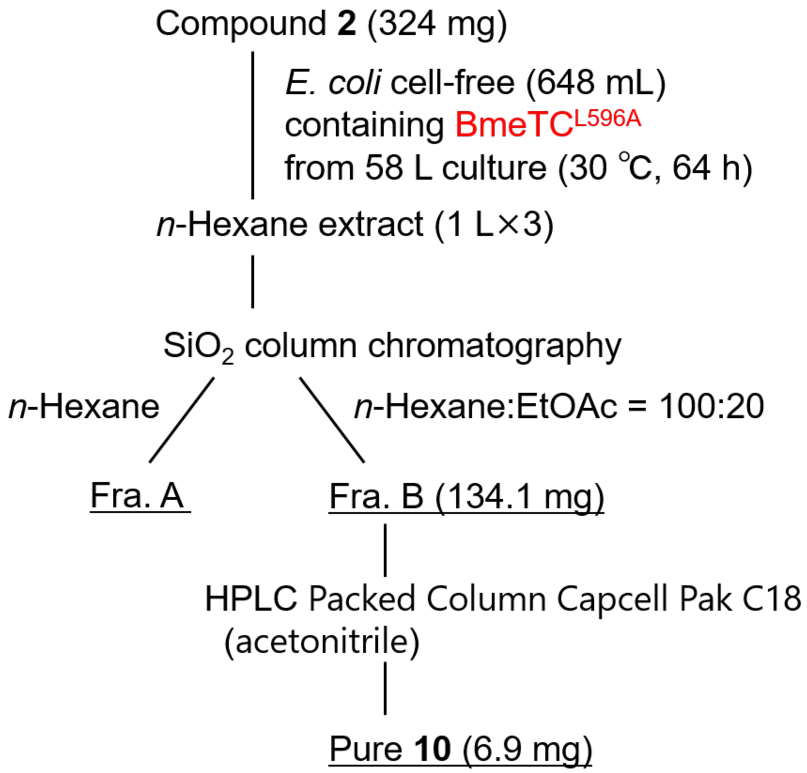

Figure S1. Isolation of **10**

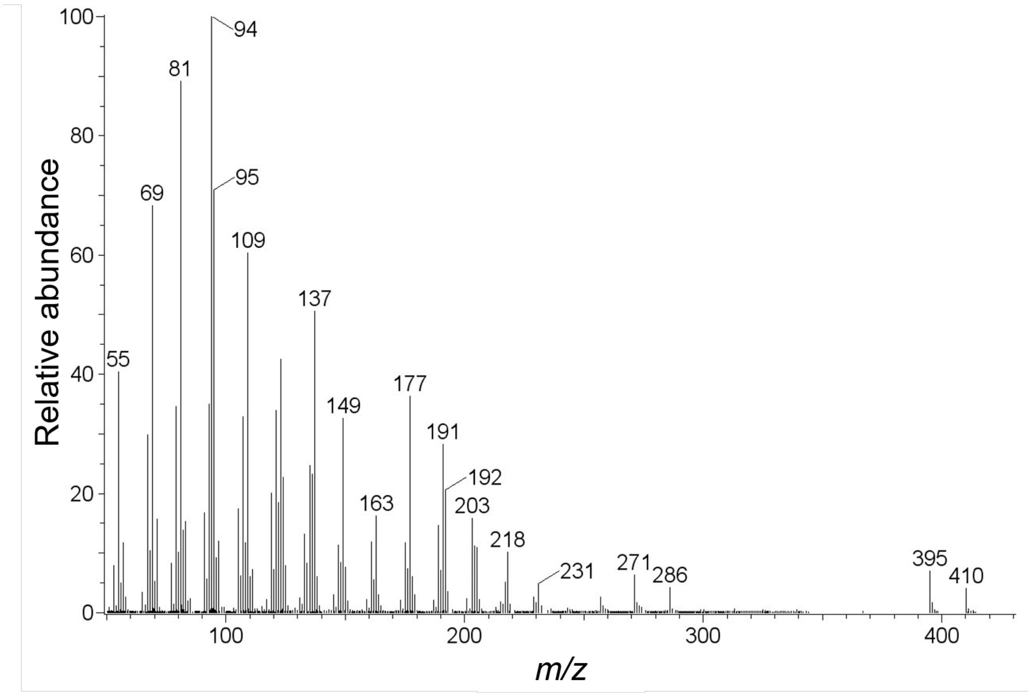

Figure S2. Mass spectrum (EI) of the compound **10**

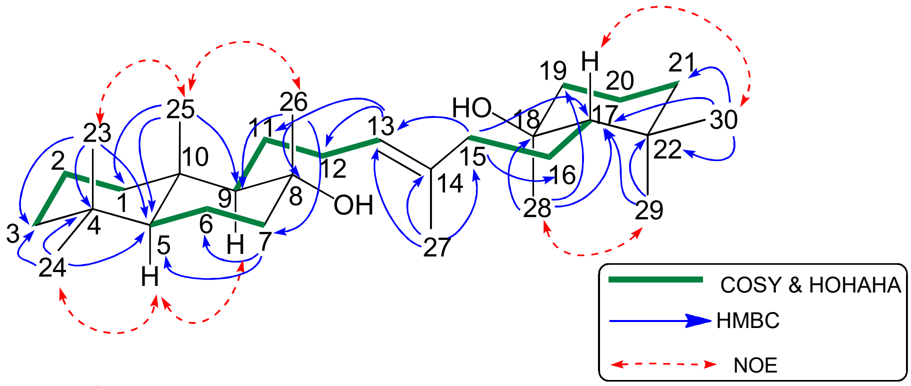

NMR data, δ ppm, in C_6D_6

NO.	^{1}H	^{13}C	NO.	^{1}H	^{13}C	NO.	^{1}H	^{13}C	NO.	^{1}H	^{13}C
1	1.04 (m); 1.76 (m)	40.1 (t)	9	1.15 (m)	60.9 (d)	17	1.23 (m)	56.2 (d)	25	0.80 (3H, s)	15.5 (q)
2	1.46 (m); 1.61 (m)	18.8 (t)	10	——	39.2 (s)	18	——	73.6 (s)	26	1.17 (3H, s)	24.2 (q)
3	1.23 (m); 1.43 (m)	41.9 (t)	11	1.46 (m); 1.73 (m)	25.8 (t)	19	1.41 (m); 1.78 (m)	44.1 (t)	27	1.91 (3H, s)	16.5 (q)
4	——	33.3 (s)	12	2.40 (m)	31.5 (t)	20	1.20 (m); 1.59 (m)	20.9 (t)	28	1.22 (3H, s)	23.6 (q)
5	0.92 (m)	56.2 (d)	13	5.60 (1H, t, 6.8 Hz)	126.2 (d)	21	1.23 (m); 1.43 (m)	42.3 (t)	29	0.87 (3H, s)[a]	26.2 (q)
6	1.20 (m); 1.59 (m)	20.9 (t)	14	——	136.0 (s)	22	——	35.6 (s)	30	1.07 (3H, s)	33.6 (q)[c]
7	1.46 (m); 1.87 (m)	45.0 (t)	15	2.45 (m)	42.9 (t)	23	0.86 (3H, s)[a]	21.6 (q)[b]			
8	——	73.6 (s)	16	1.61 (m); 1.84 (m)	25.0 (t)	24	0.96 (3H, s)	33.1 (q)[c]			

a-c: these signals are exchageable.

Figure S3. NMR assignment of compound **10** measured in C_6D_6.

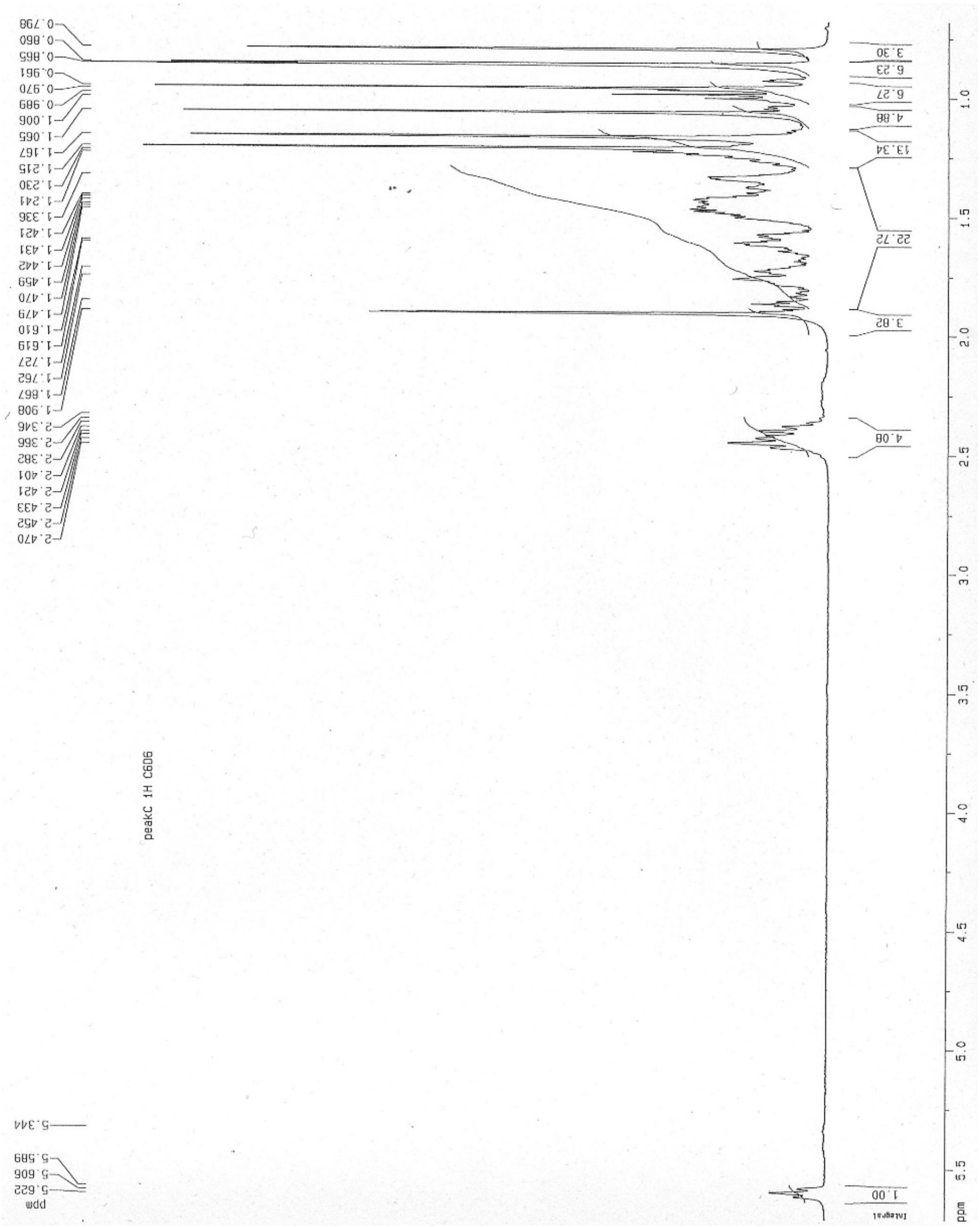

Figure S4. ^{1}H NMR spectrum of **10** measured in C_6D_6

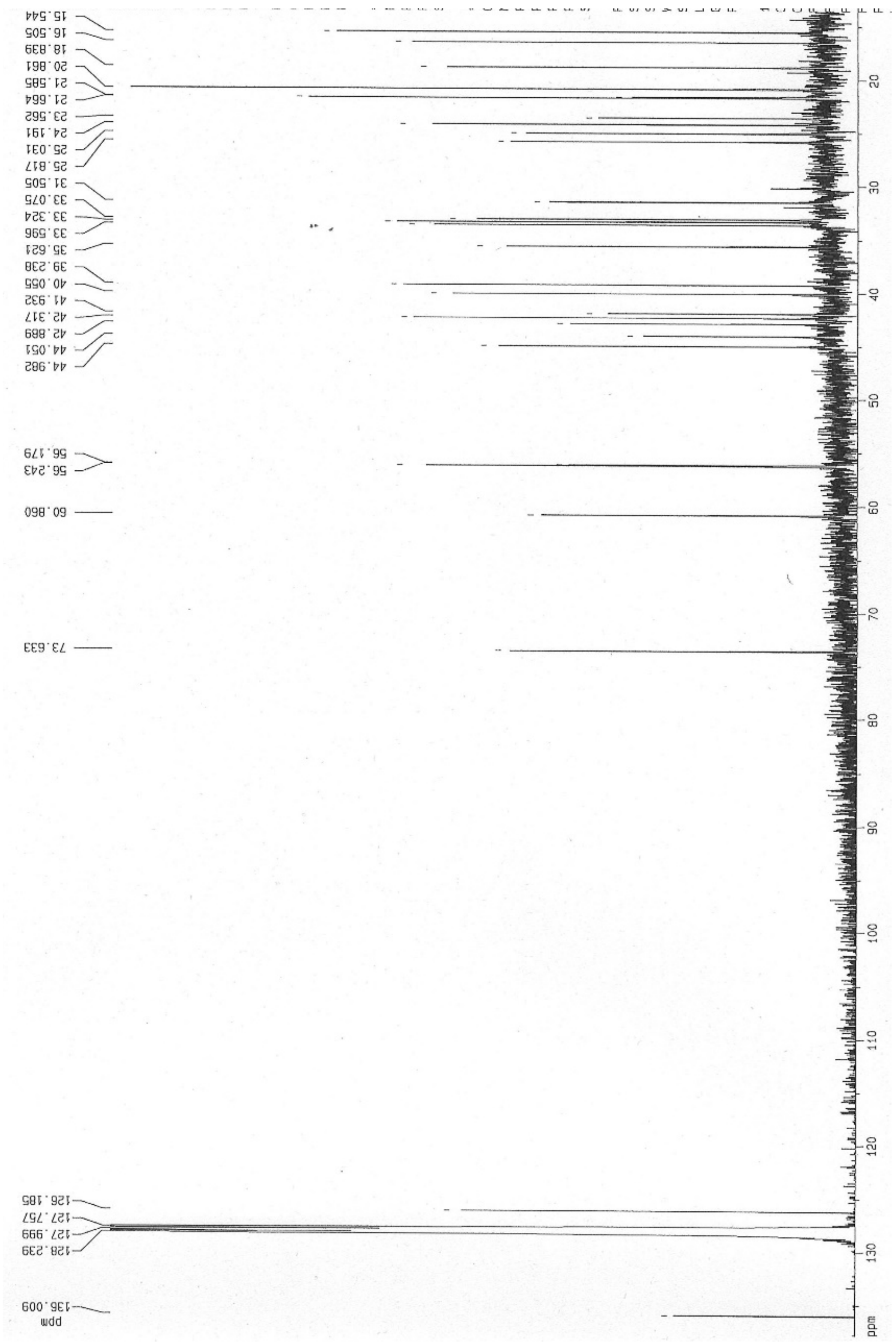

Figure S5. ^{13}C NMR spectrum of **10** measured in C_6D_6

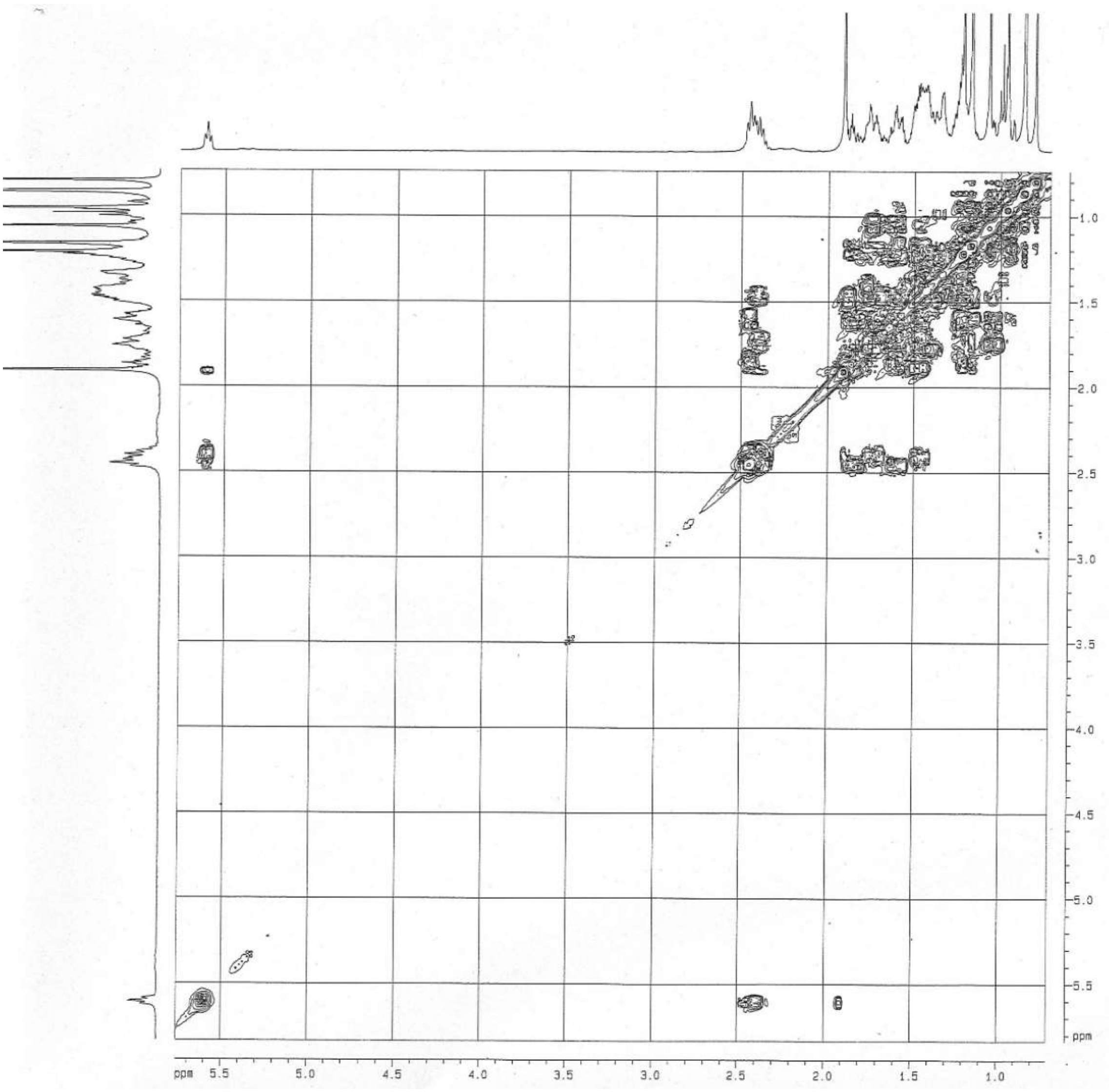

Figure S6. ^{1}H-^{1}H COSY spectrum of **10** measured in C_6D_6

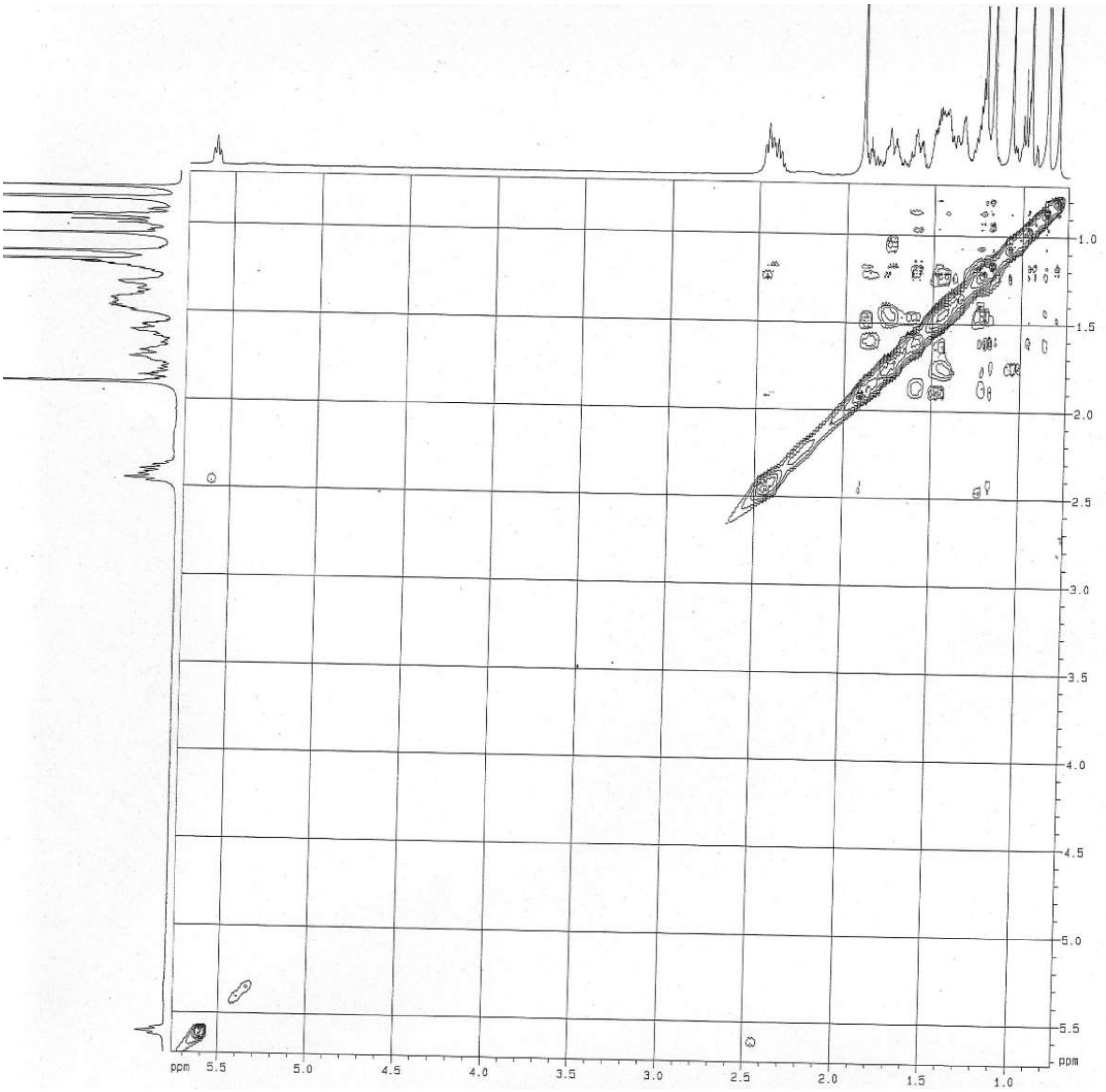

Figure S7. NOESY spectrum of **10** measured in C_6D_6

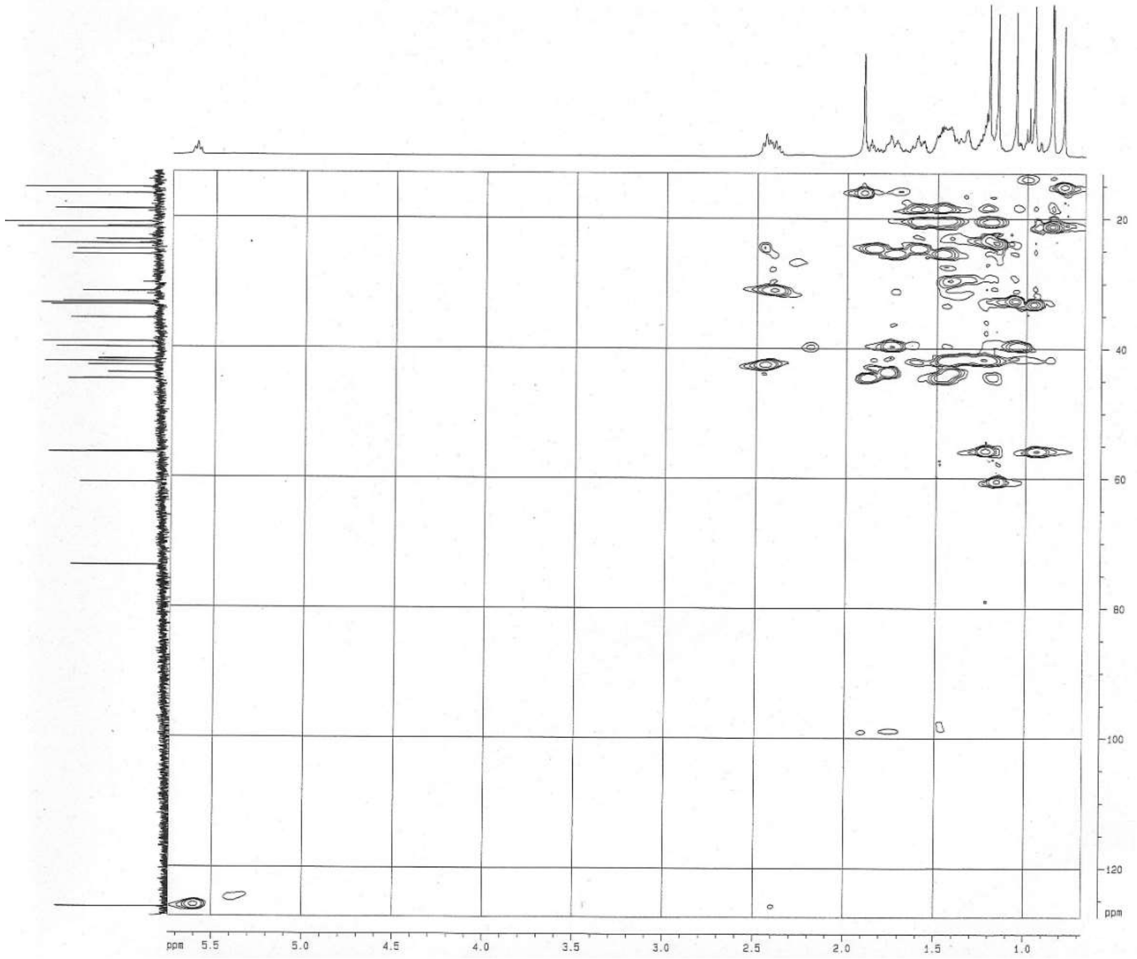

Figure S8. HSQC spectrum of **10** measured in C_6D_6

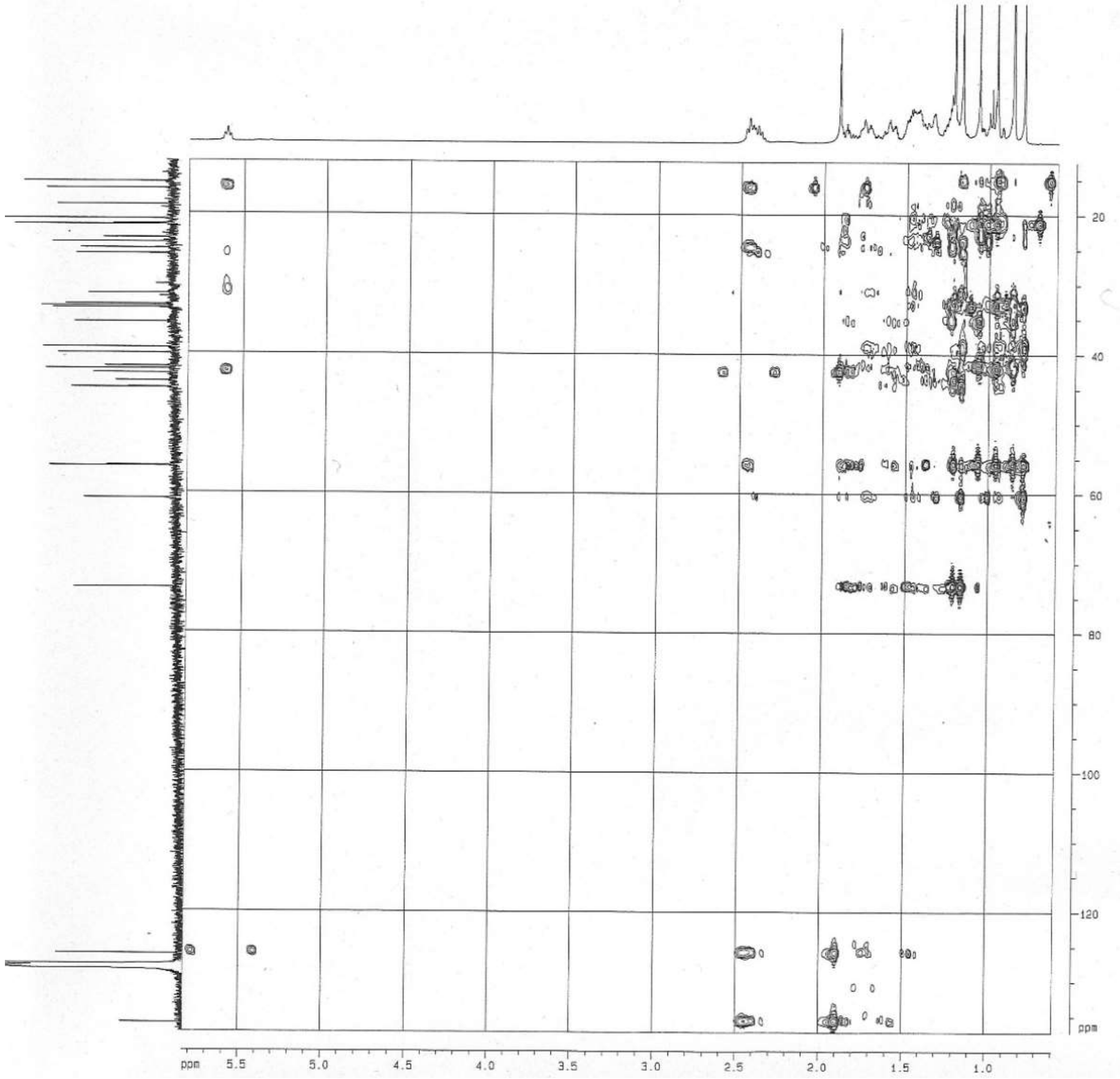

Figure S9. HMBC spectrum of **10** measured in C_6D_6

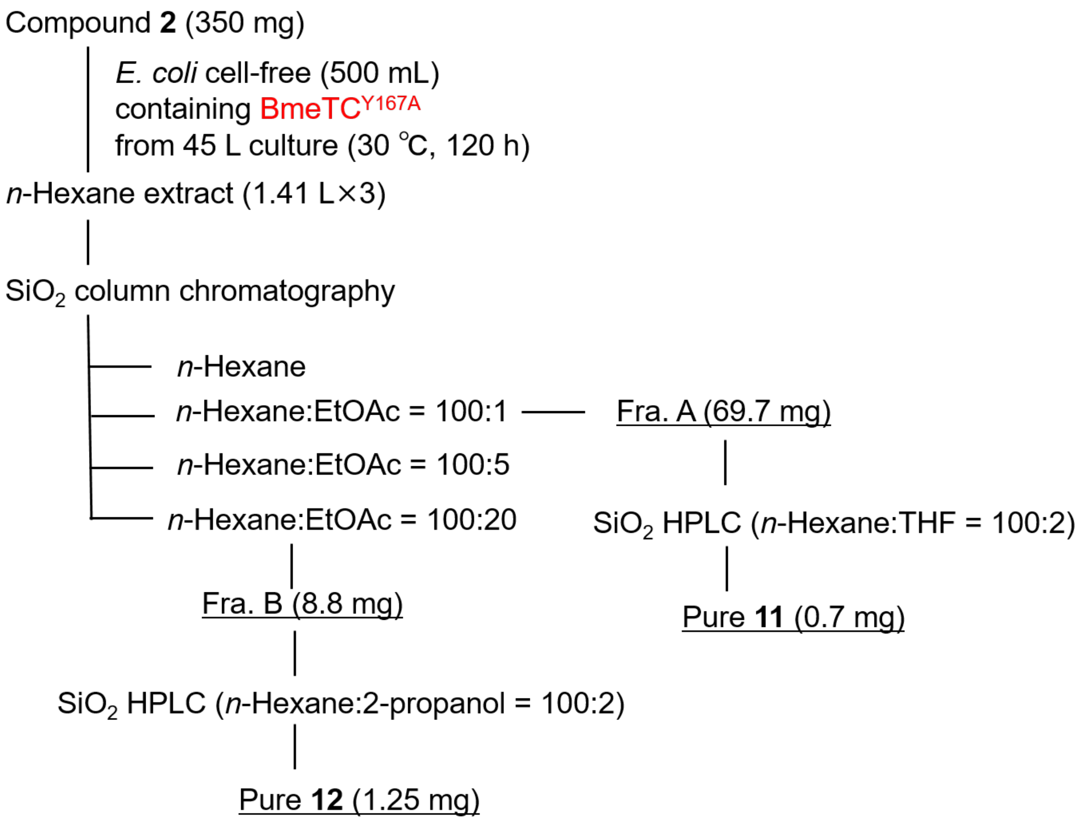

Figure S10. Isolation of **11** and **12.**

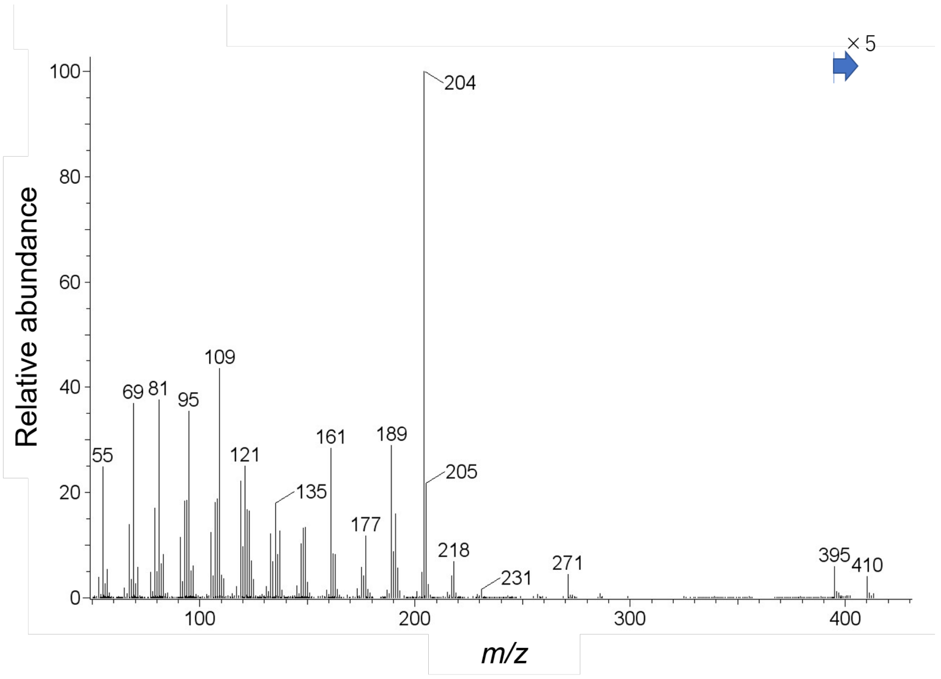

Figure S11. Mass spectrum (EI) of the compound **11.**

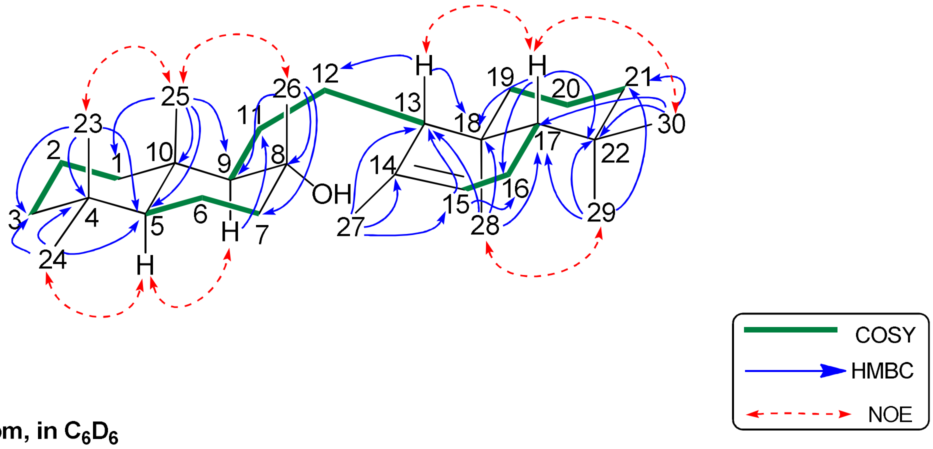

NMR data, δ ppm, in C_6D_6

NO.	^{1}H	^{13}C	NO.	^{1}H	^{13}C	NO.	^{1}H	^{13}C	NO.	^{1}H	^{13}C
1	1.06 (m); 2.08 (m)	39.5 (t)[a]	9	0.95 (m)	62.9 (d)	17	1.35 (m)	50.5 (d)	25	0.84 (s)	15.7 (q)
2	1.50 (m); 1.65 (m)	18.9 (t)[b]	10	——	39.0 (s)	18	——	37.0 (s)	26	1.19 (s)	24.2 (q)
3	1.22 (m); 1.46 (m)	42.3 (t)[c]	11	1.20 (m); 1.89 (m)	29.3 (t)	19	1.06 (m); 2.08 (m)	39.8 (t)[a]	27	2.13 (s)	22.9 (q)
4	——	33.1 (s)[d]	12	1.34 (m); 1.60 (m)	32.1 (t)	20	1.50 (m); 1.65 (m)	19.3 (t)[b]	28	1.05 (s)	13.9 (q)
5	0.85 (m)	56.1 (d)	13	1.72 (m)	56.6 (d)	21	1.22 (m); 1.46 (m)	42.7 (t)[c]	29	1.02 (s)	22.1 (q)
6	1.60 (m)	20.6 (t)	14	——	136.1 (s)	22	——	33.3 (s)[d]	30	1.00 (s)	33.6 (q)[e]
7	1.28 (m); 1.81 (m)	45.1 (t)	15	5.62 (1H, m)	122.0 (d)	23	0.87 (s)	21.6 (q)			
8	——	73.6 (s)	16	2.08 (m)	24.3 (t)	24	0.97 (s)	33.4 (q)[e]			

a-e: exchangeable, benzene: 7.26 ppm (^{1}H); 128.0 ppm (^{13}C)

Figure S12. NMR assignment of compound **11** measured in C_6D_6.

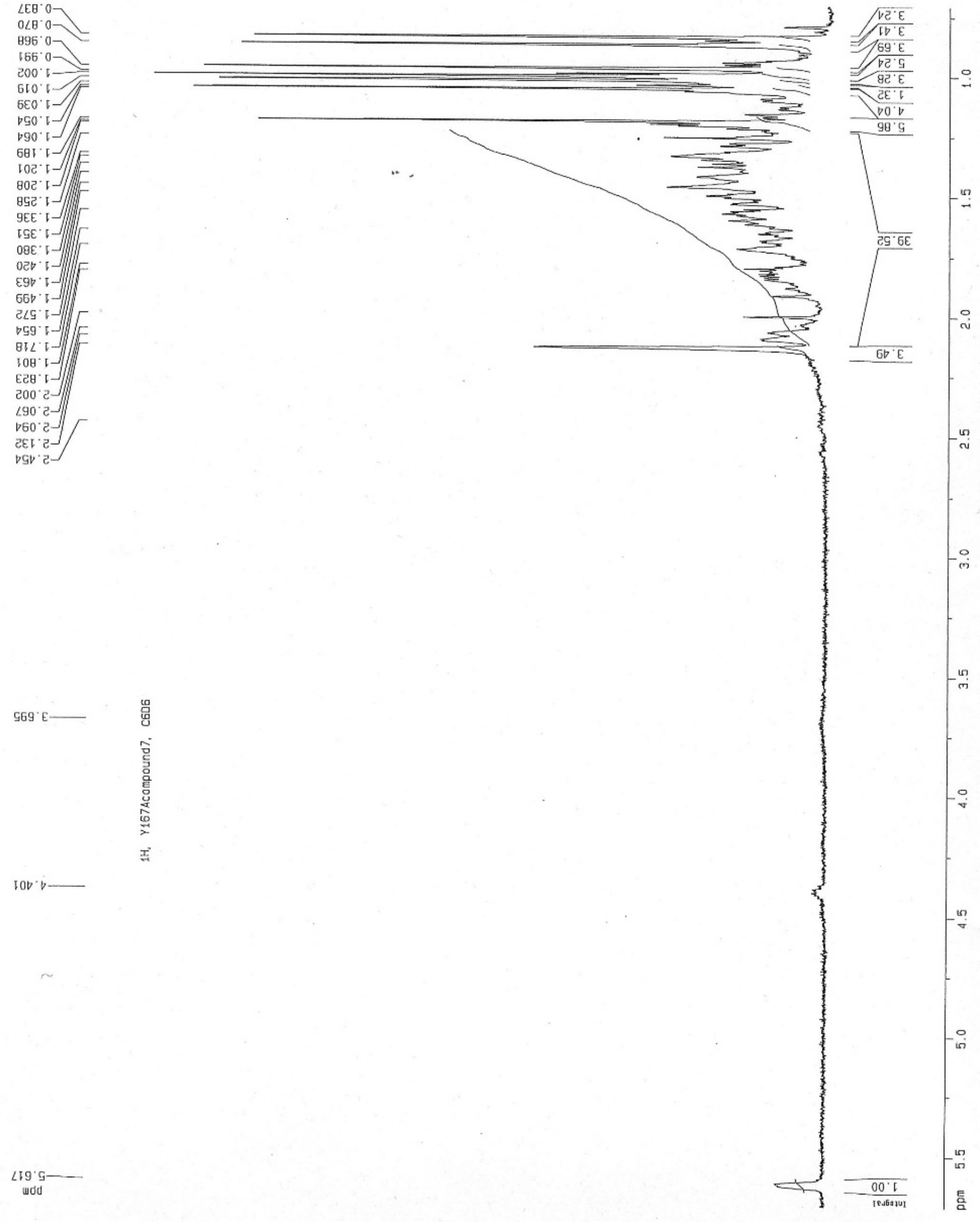

Figure S13. ^{1}H NMR spectrum of **11** measured in C_6D_6

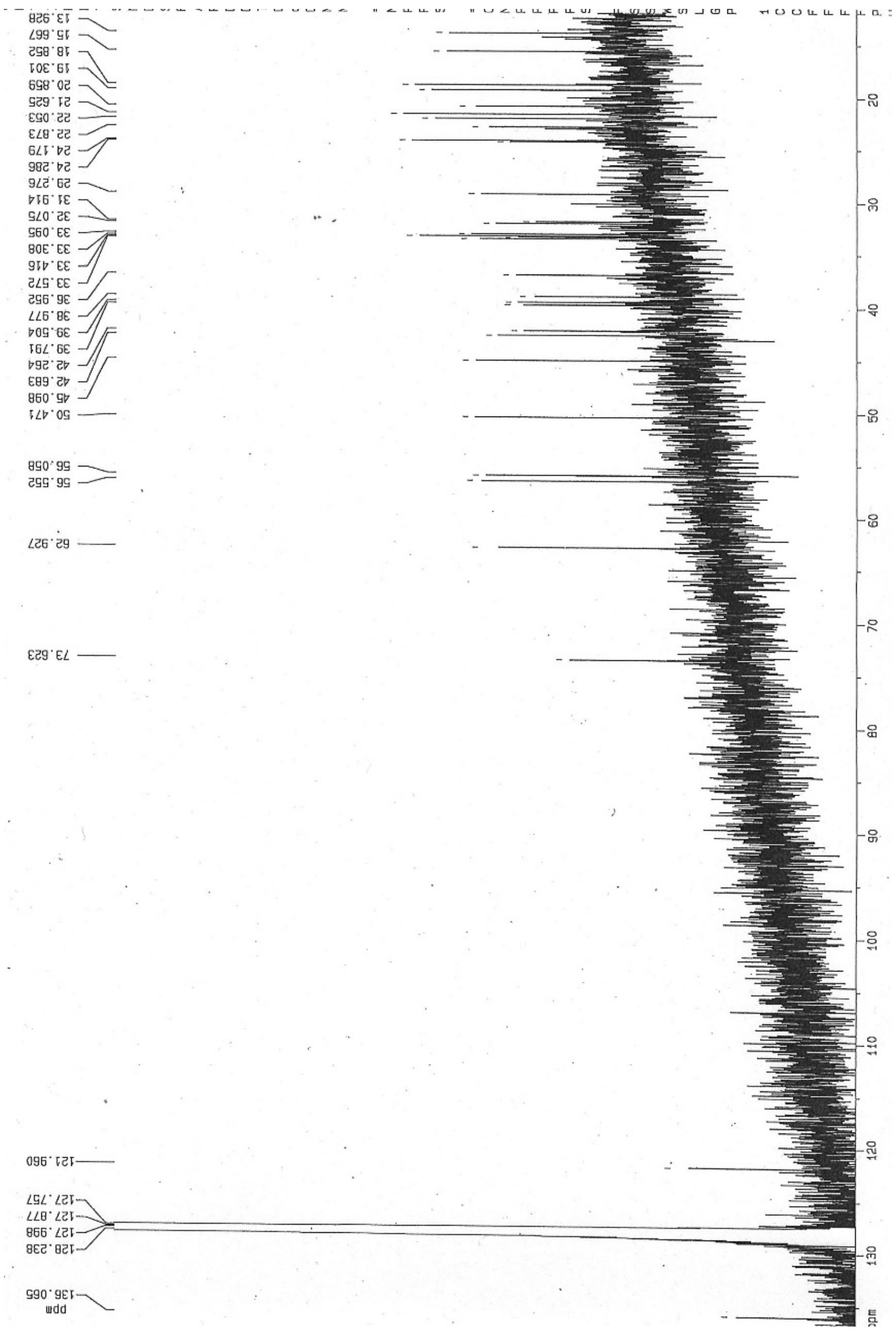

Figure S14. ^{13}C NMR spectrum of **11** measured in C_6D_6

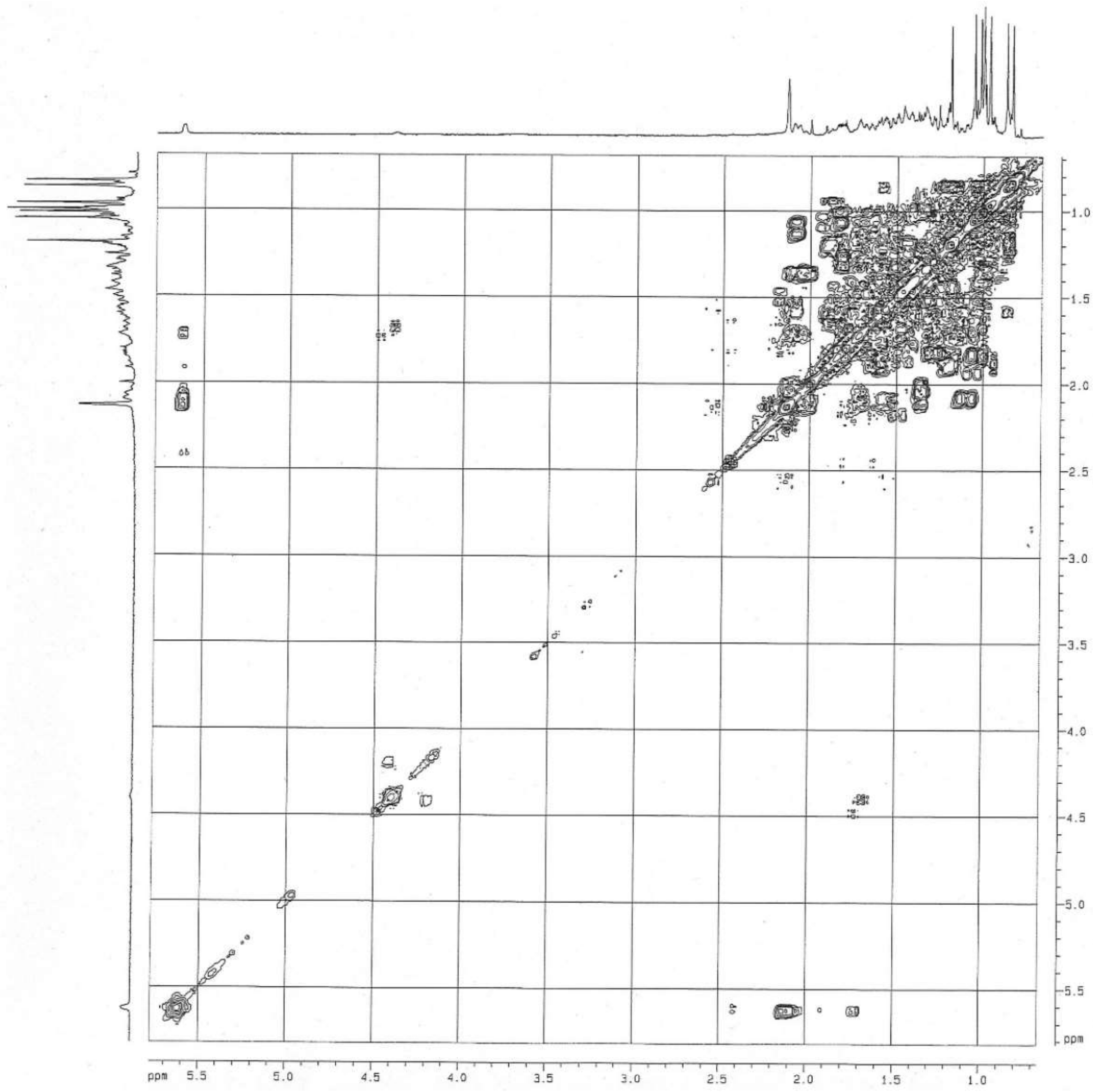

Figure S15. ^{1}H-^{1}H COSY spectrum of **11** measured in C_6D_6

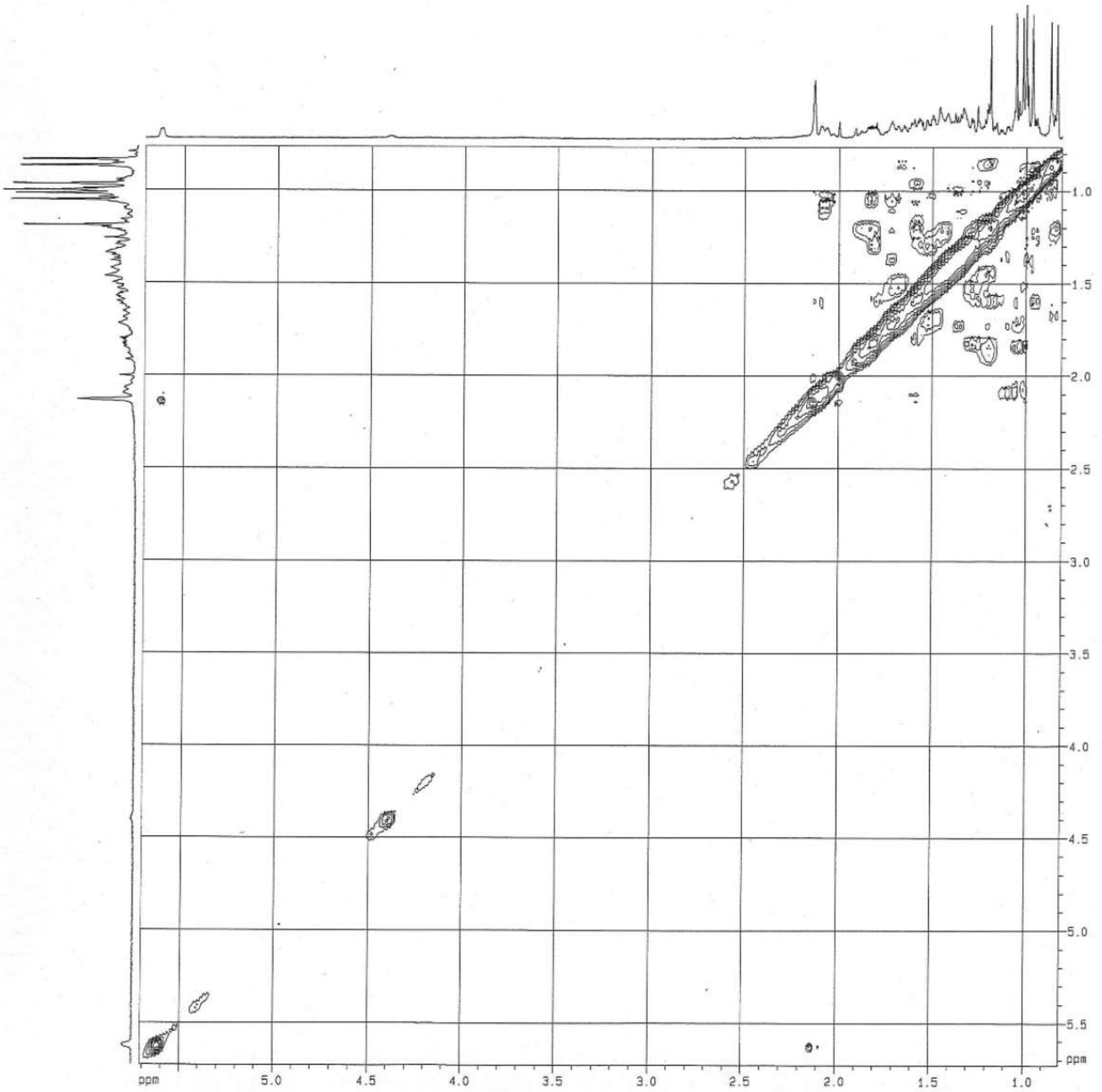

Figure S16. NOESY spectrum of **11** measured in C_6D_6

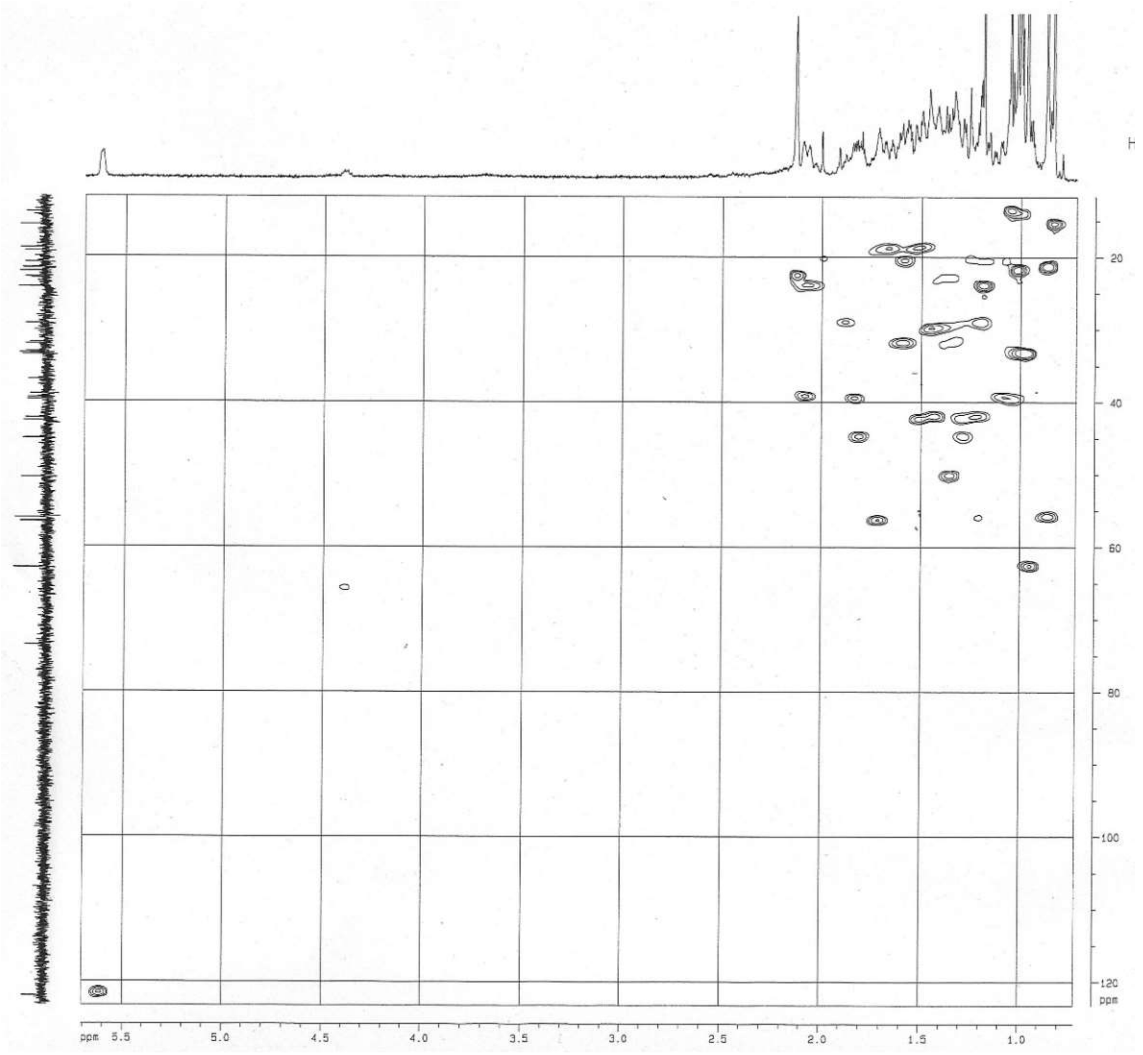

Figure S17. HSQC spectrum of **11** measured in C_6D_6

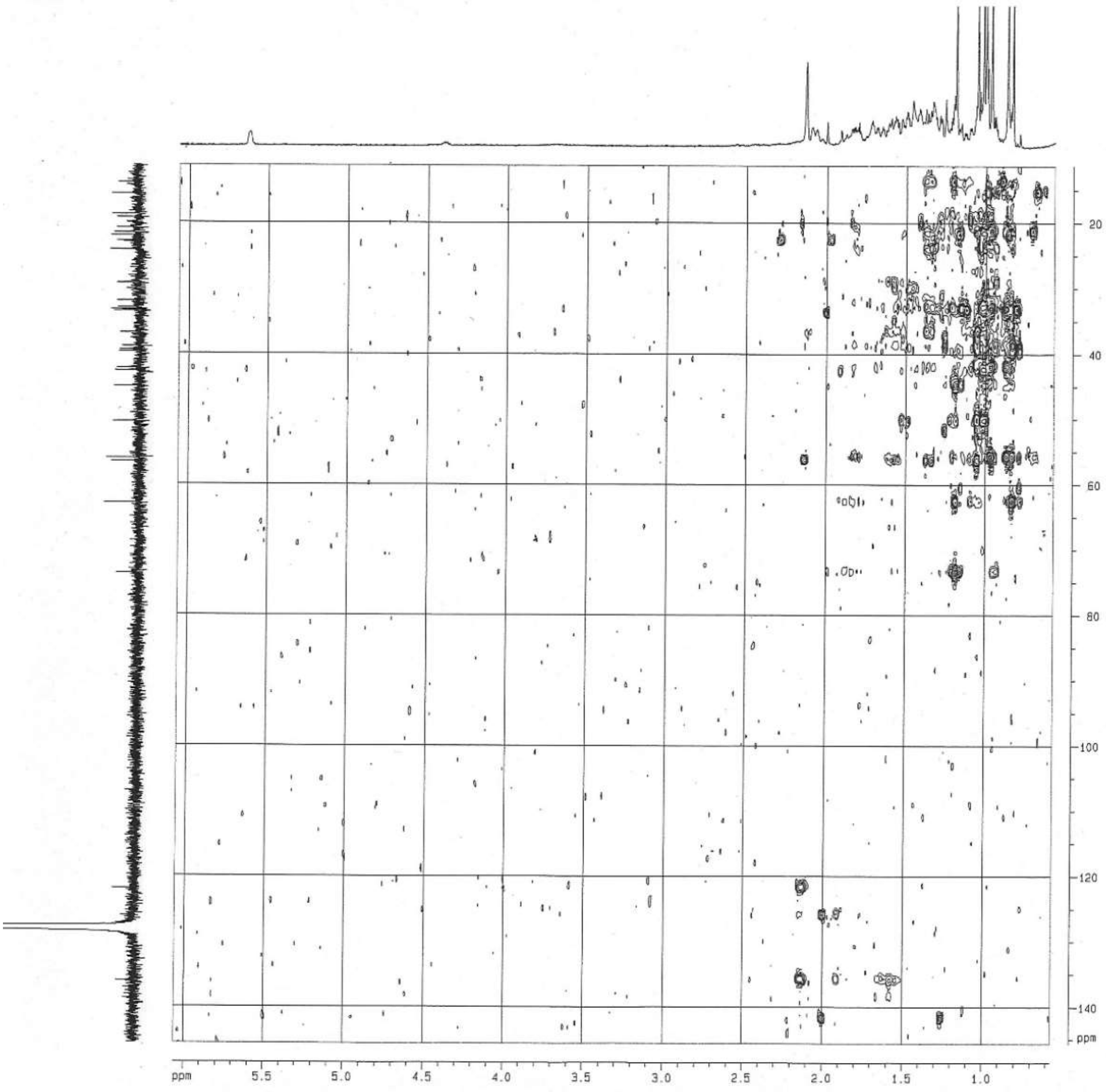

Figure S18. HMBC spectrum of **11** measured in C_6D_6

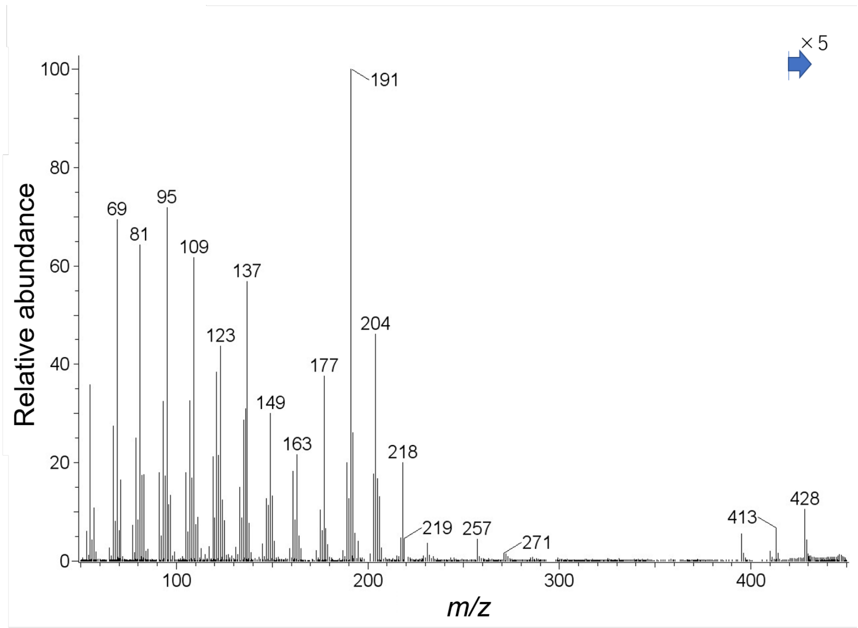

Figure S19. Mass spectrum (EI) of the compound **12.**

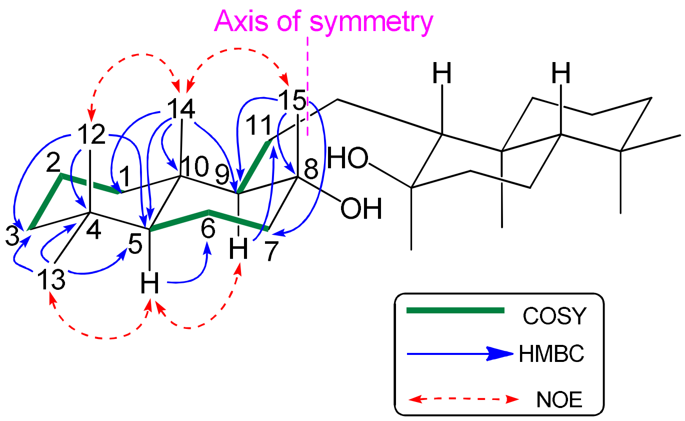

NMR data, δ ppm, in C_6D_6

NO.	^{1}H	^{13}C	NO.	^{1}H	^{13}C
1	0.95 (m); 1.68 (m)	39.9 (t)	9	1.25 (m)	63.6 (d)
2	1.35 (m); 1.54 (m)	18.9 (t)	10	——	38.8 (s)
3	1.10 (m); 1.32 (m)	42.3 (t)	11	1.49 (m); 1.64 (m)	29.9 (t)
4	——	33.3 (s)	12	0.76 (s)	21.6 (q)
5	0.90 (m)	56.3 (d)	13	0.84 (s)	33.5 (q)
6	1.23 (m); 1.56 (m)	20.8 (t)	14	0.76 (s)	16.0 (q)
7	1.58 (m); 1.90 (m)	44.1 (t)	15	1.25 (s)	24.5 (q)
8	——	74.5 (s)			

(benzene, ^{1}H: 7.15 ppm; ^{13}C: 128.0 ppm)

Figure S20. NMR assignment of compound **12** measured in C_6D_6.

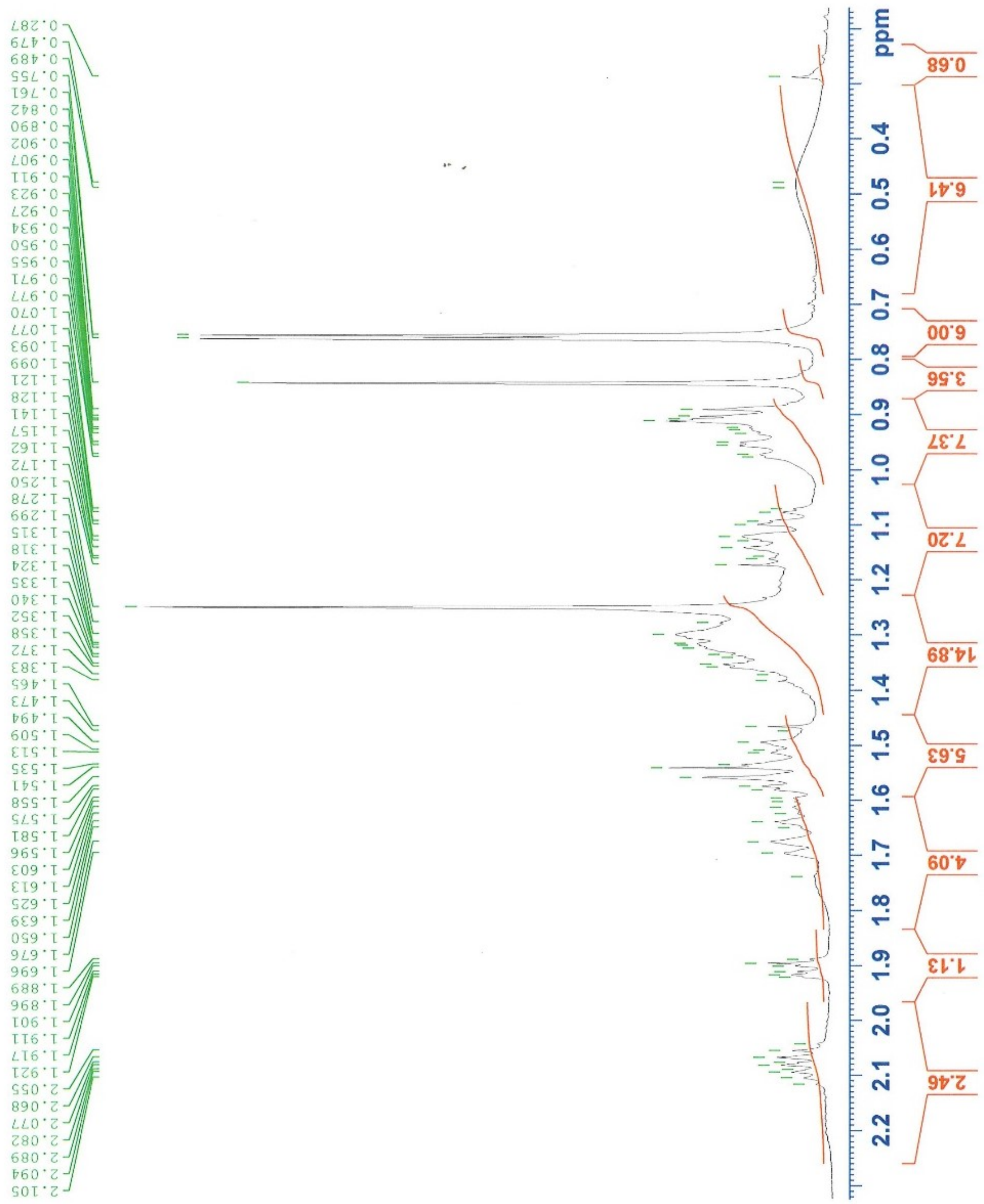

Figure S21. ^{1}H NMR spectrum of **12** measured in C_6D_6

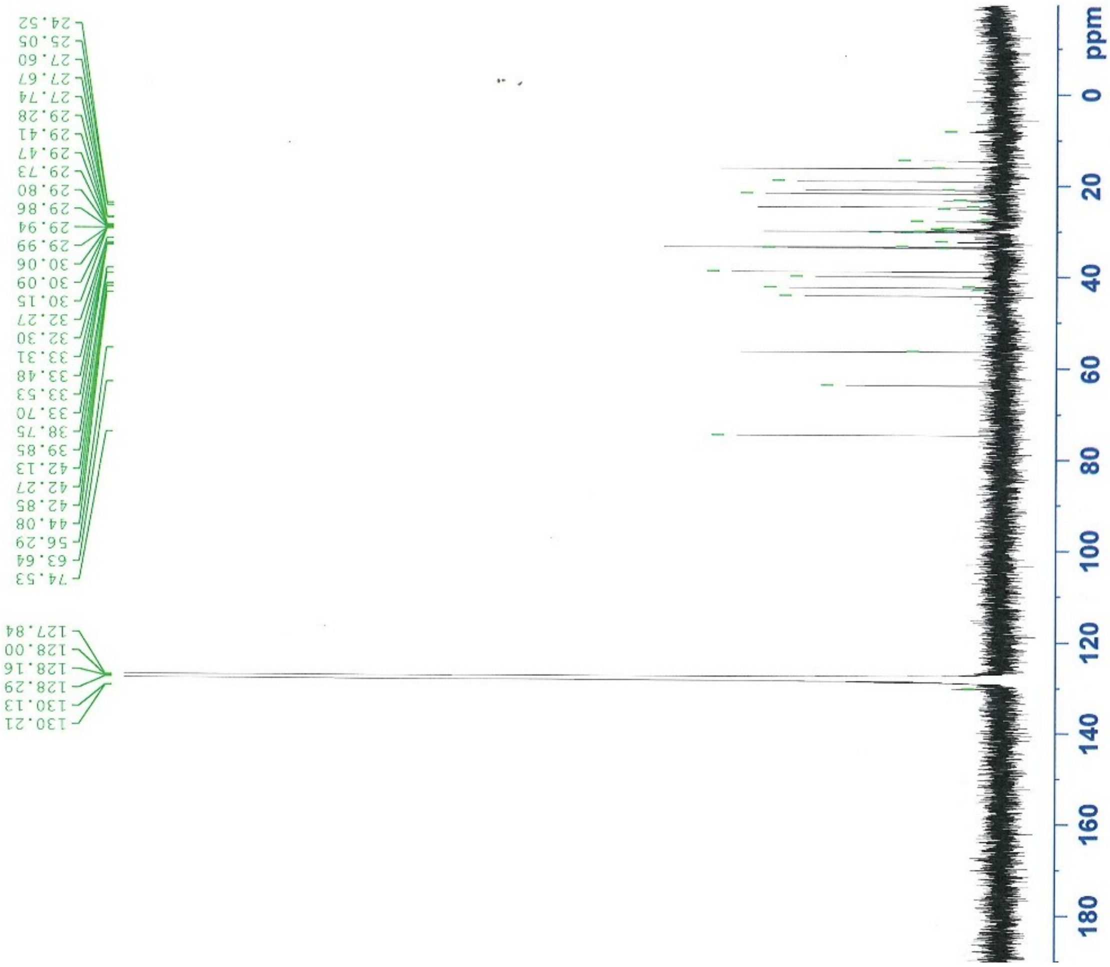

Figure S22. ^{13}C NMR spectrum of **12** measured in C_6D_6

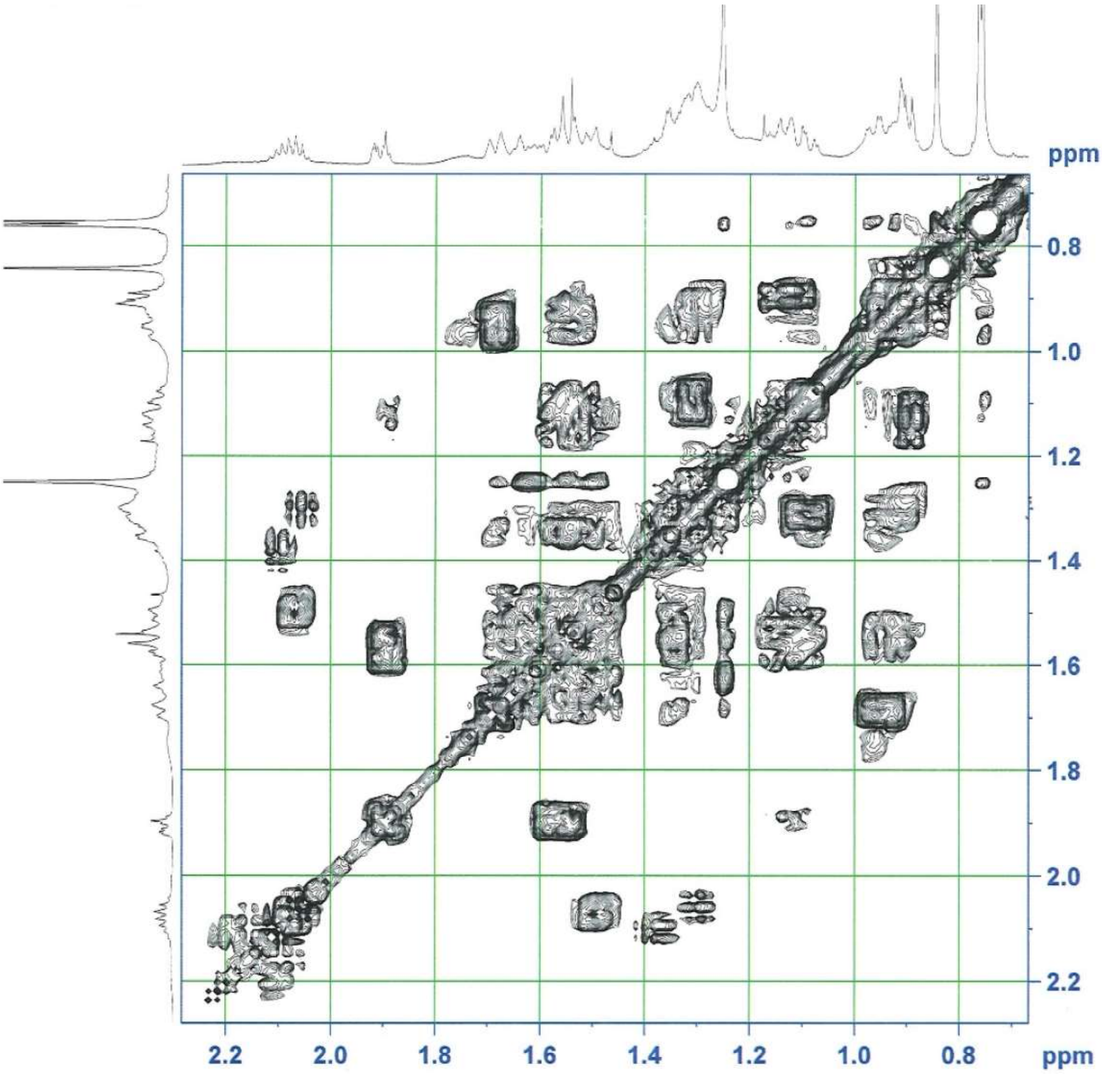

Figure S23. ^1H-^1H COSY spectrum of **12** measured in C_6D_6

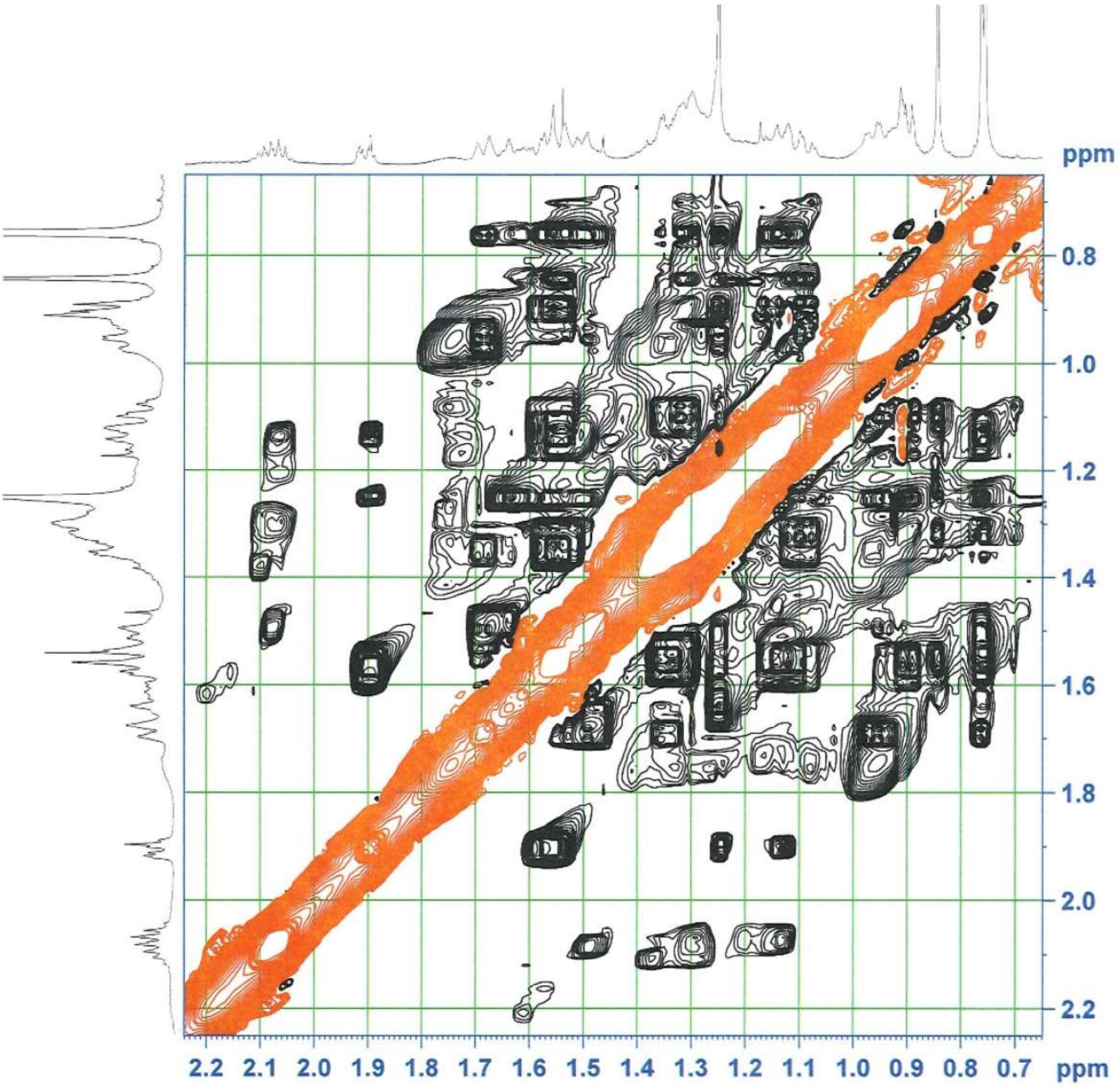

Figure S24. NOESY spectrum of **12** measured in C_6D_6

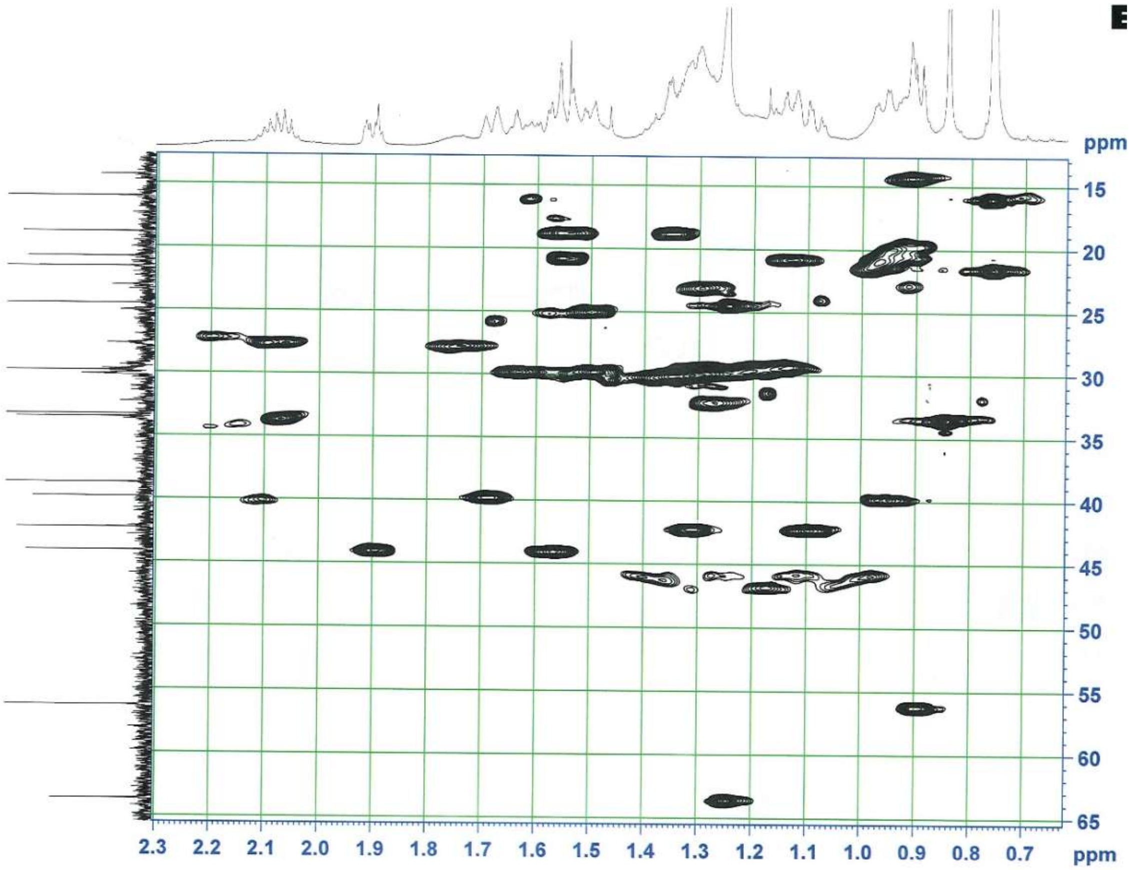

Figure S25. HSQC spectrum of **12** measured in C_6D_6

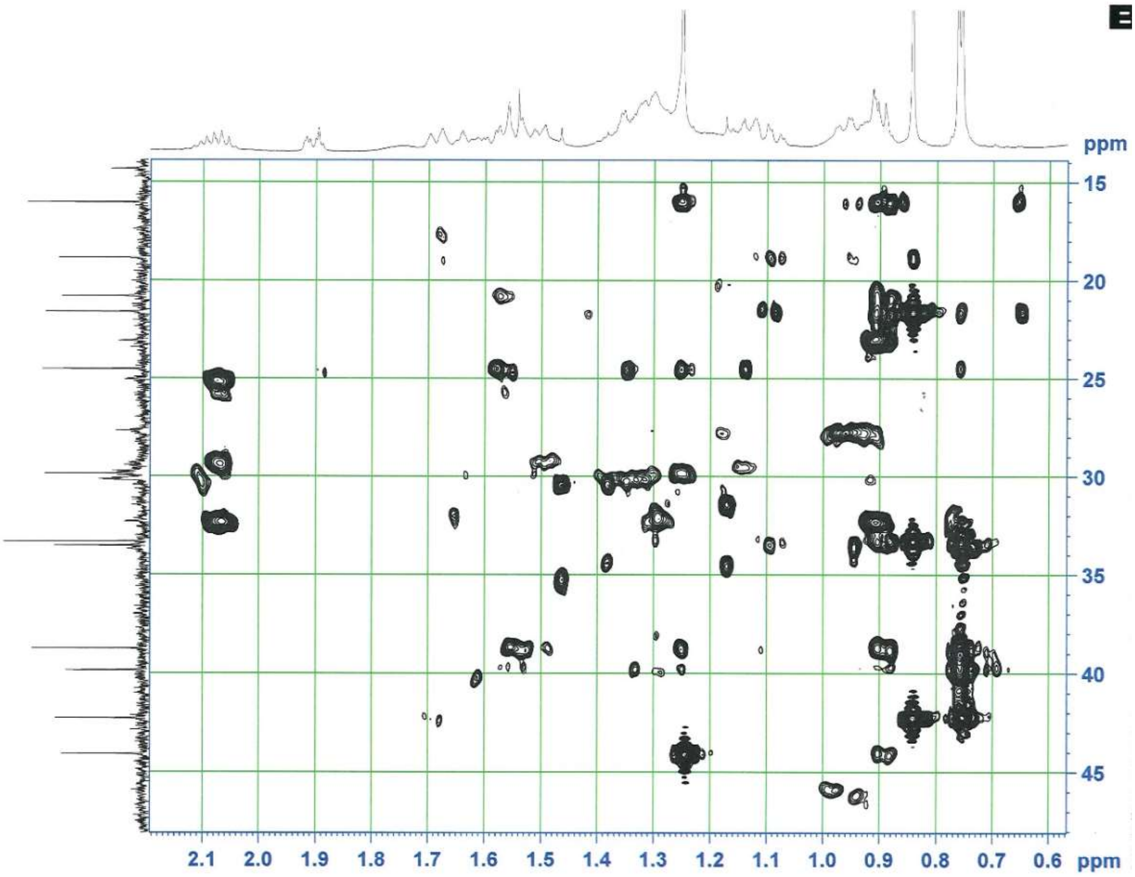

Figure S26. HMBC spectrum of **12** measured in C_6D_6

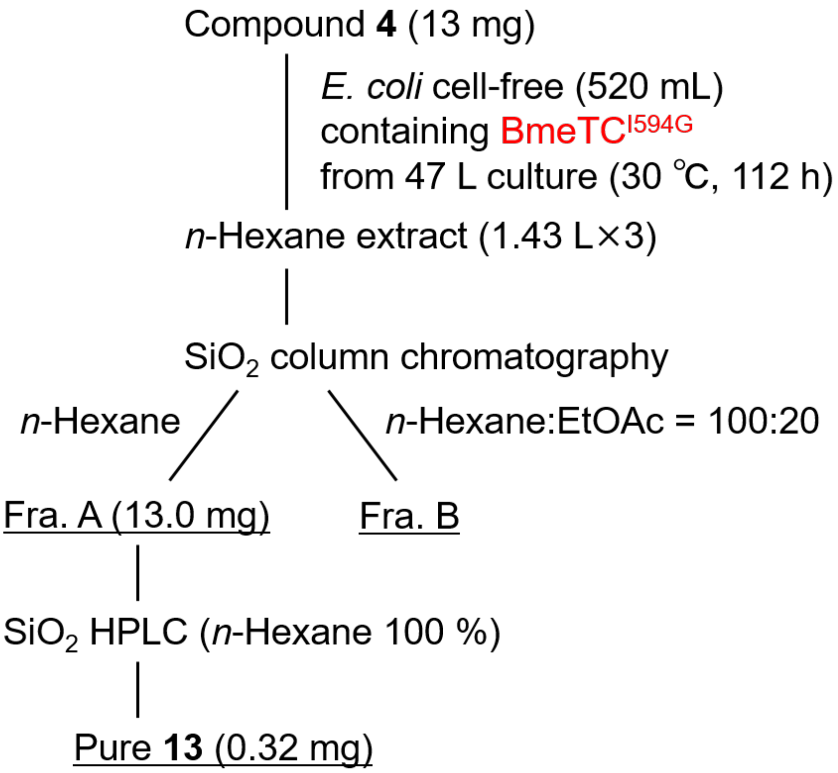

Fig. S27. Isolation of **13.**

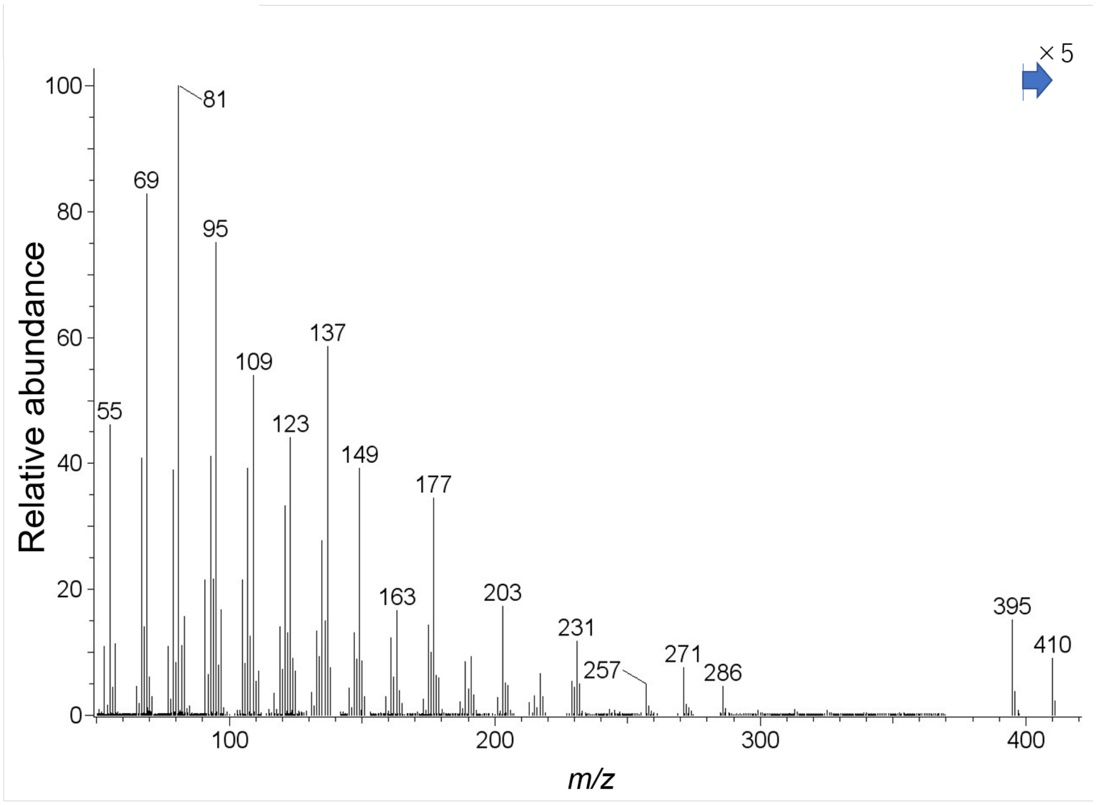

Figure S28. Mass spectrum (EI) of the compound **13**

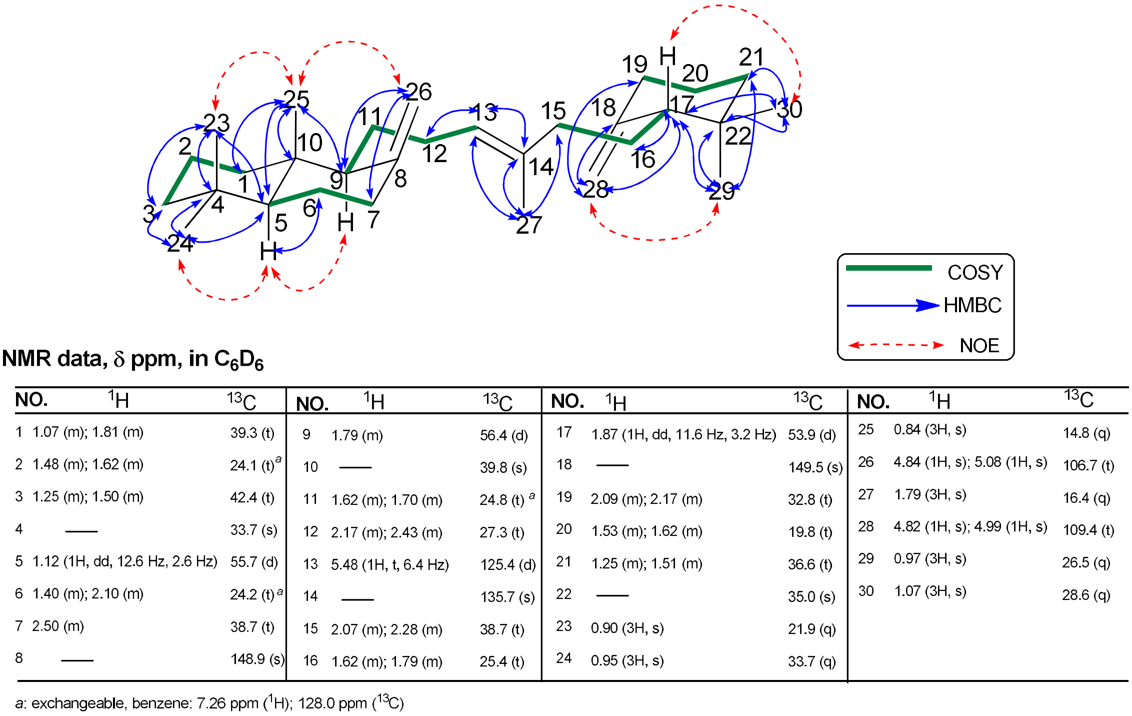

NMR data, δ ppm, in C_6D_6

NO.	^1H	^{13}C	NO.	^1H	^{13}C	NO.	^1H	^{13}C	NO.	^1H	^{13}C
1	1.07 (m); 1.81 (m)	39.3 (t)	9	1.79 (m)	56.4 (d)	17	1.87 (1H, dd, 11.6 Hz, 3.2 Hz)	53.9 (d)	25	0.84 (3H, s)	14.8 (q)
2	1.48 (m); 1.62 (m)	24.1 (t)[a]	10	——	39.8 (s)	18	——	149.5 (s)	26	4.84 (1H, s); 5.08 (1H, s)	106.7 (t)
3	1.25 (m); 1.50 (m)	42.4 (t)	11	1.62 (m); 1.70 (m)	24.8 (t) [a]	19	2.09 (m); 2.17 (m)	32.8 (t)	27	1.79 (3H, s)	16.4 (q)
4	——	33.7 (s)	12	2.17 (m); 2.43 (m)	27.3 (t)	20	1.53 (m); 1.62 (m)	19.8 (t)	28	4.82 (1H, s); 4.99 (1H, s)	109.4 (t)
5	1.12 (1H, dd, 12.6 Hz, 2.6 Hz)	55.7 (d)	13	5.48 (1H, t, 6.4 Hz)	125.4 (d)	21	1.25 (m); 1.51 (m)	36.6 (t)	29	0.97 (3H, s)	26.5 (q)
6	1.40 (m); 2.10 (m)	24.2 (t)[a]	14	——	135.7 (s)	22	——	35.0 (s)	30	1.07 (3H, s)	28.6 (q)
7	2.50 (m)	38.7 (t)	15	2.07 (m); 2.28 (m)	38.7 (t)	23	0.90 (3H, s)	21.9 (q)			
8	——	148.9 (s)	16	1.62 (m); 1.79 (m)	25.4 (t)	24	0.95 (3H, s)	33.7 (q)			

a: exchangeable, benzene: 7.26 ppm (^1H); 128.0 ppm (^{13}C)

Figure S29. NMR assignment of compound **13** measured in C_6D_6.

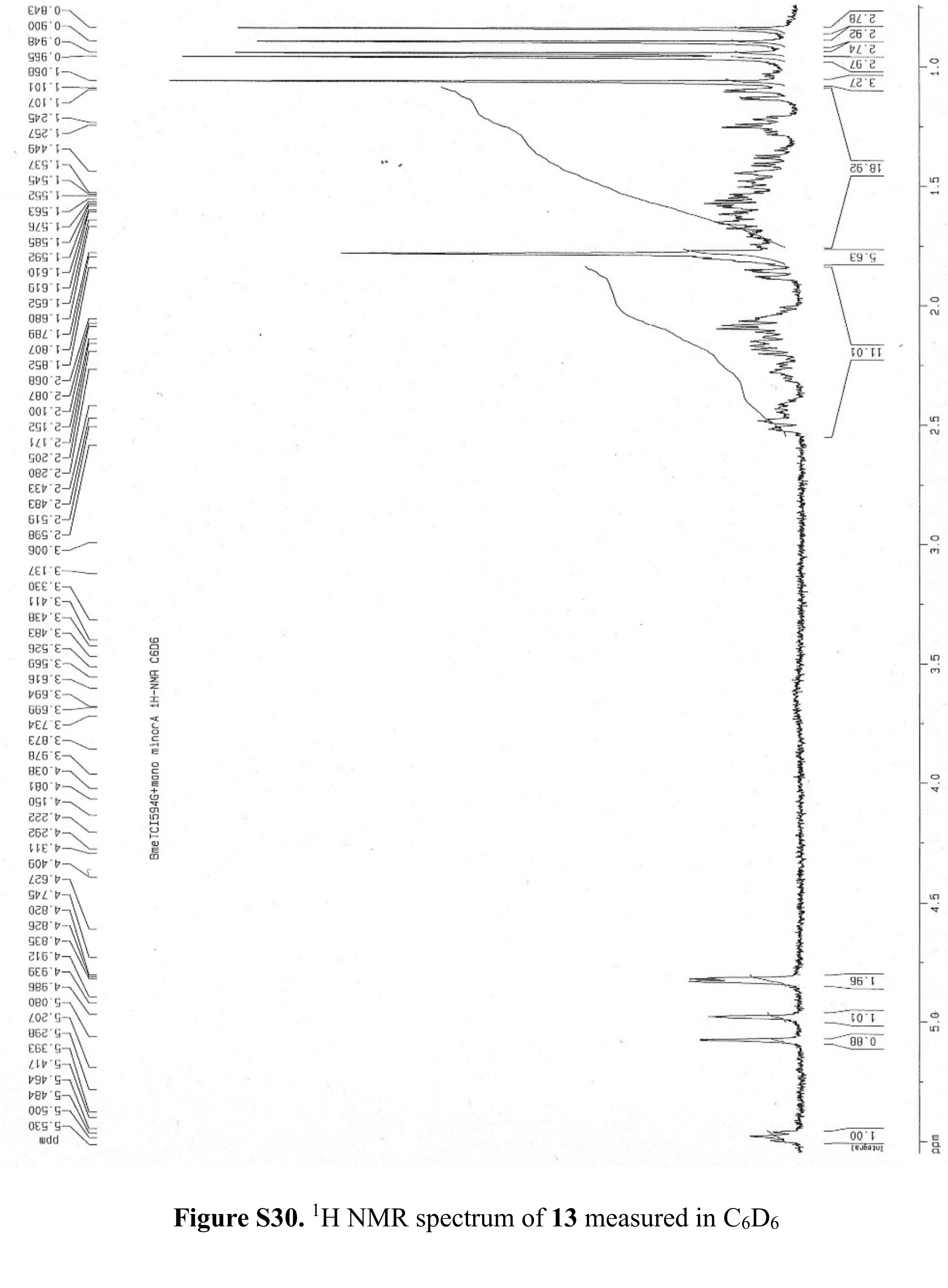

Figure S30. ^{1}H NMR spectrum of **13** measured in C_6D_6

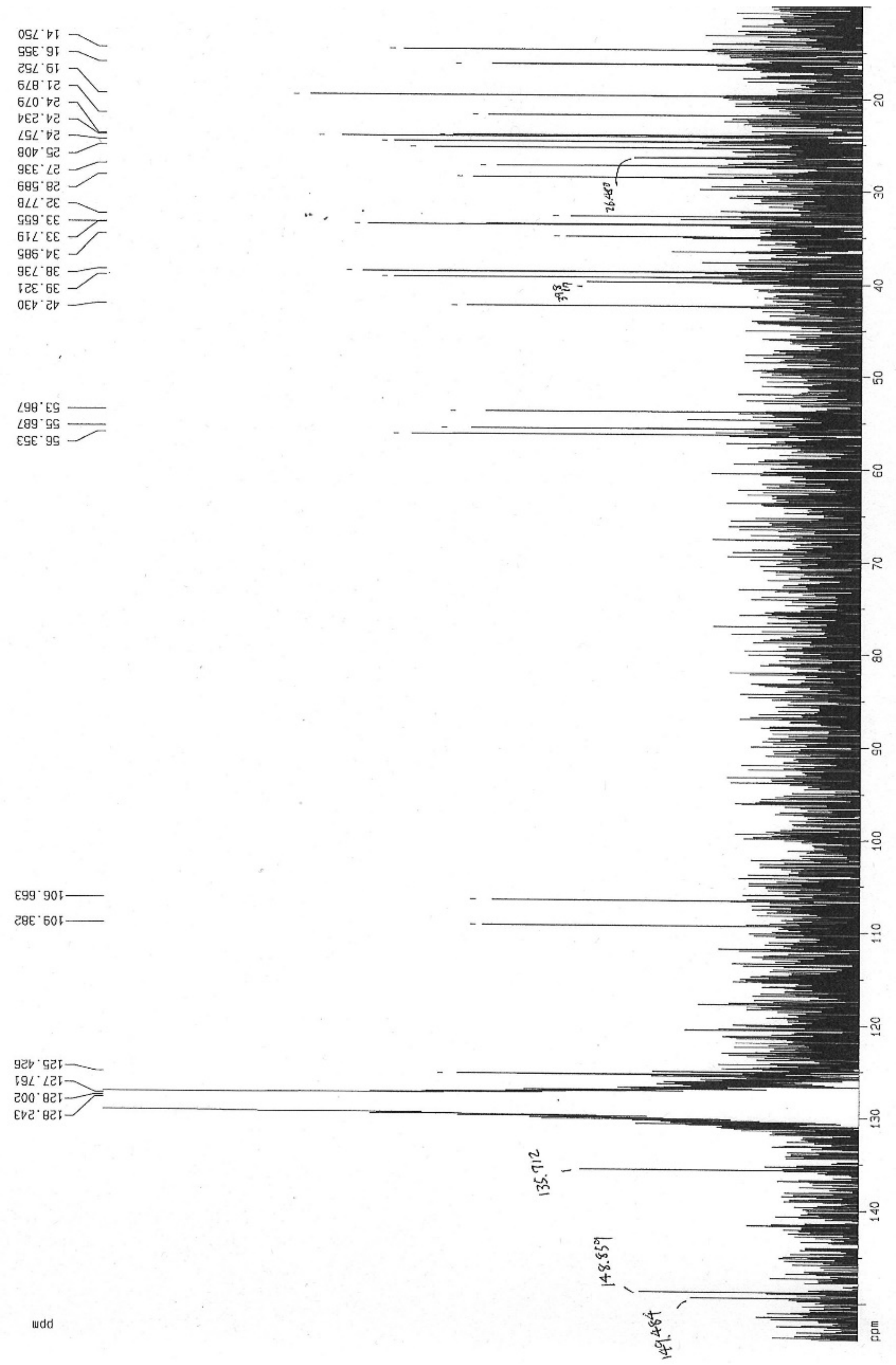

Figure S31. ^{13}C NMR spectrum of **13** measured in C_6D_6

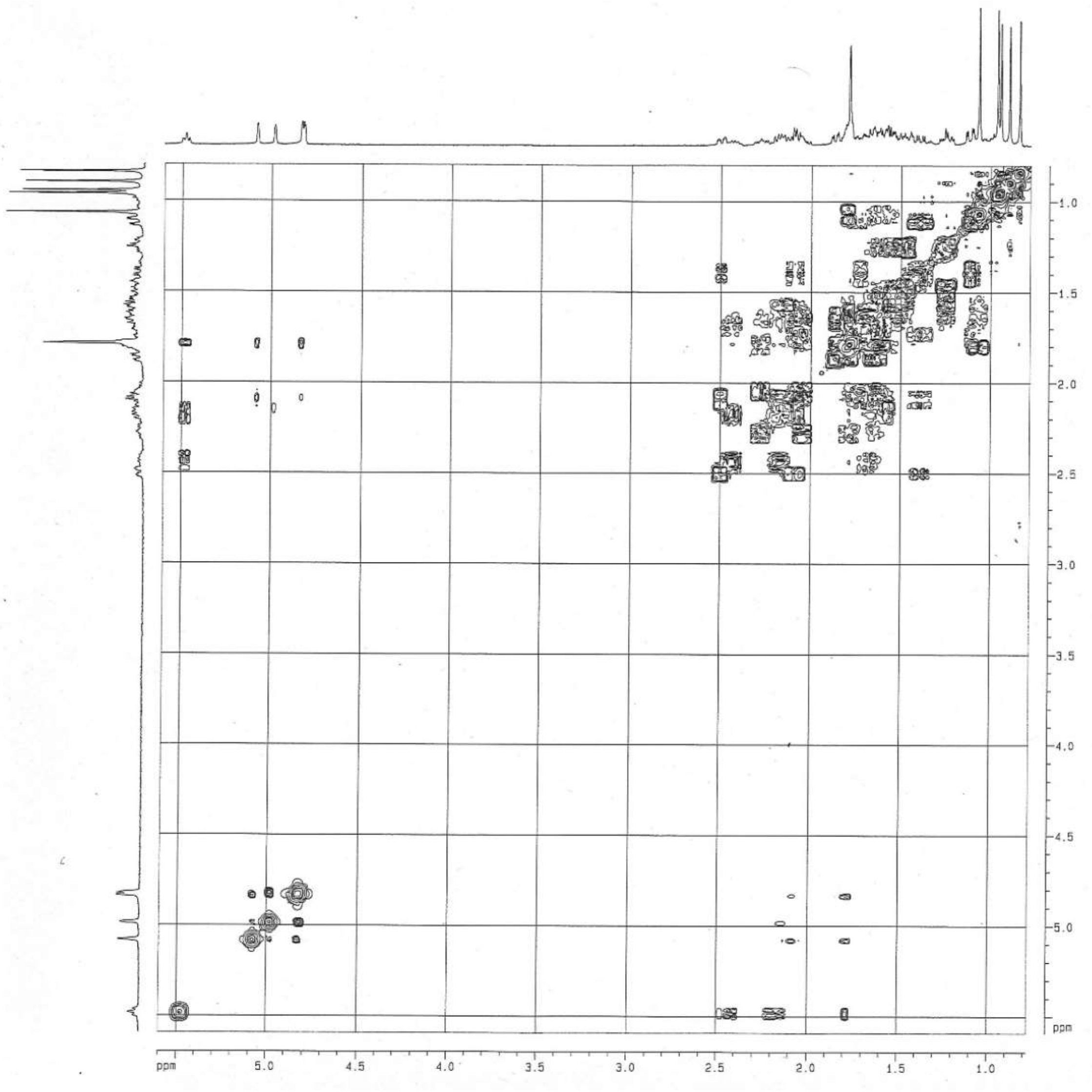

Figure S32. ^{1}H-^{1}H COSY spectrum of **13** measured in C_6D_6

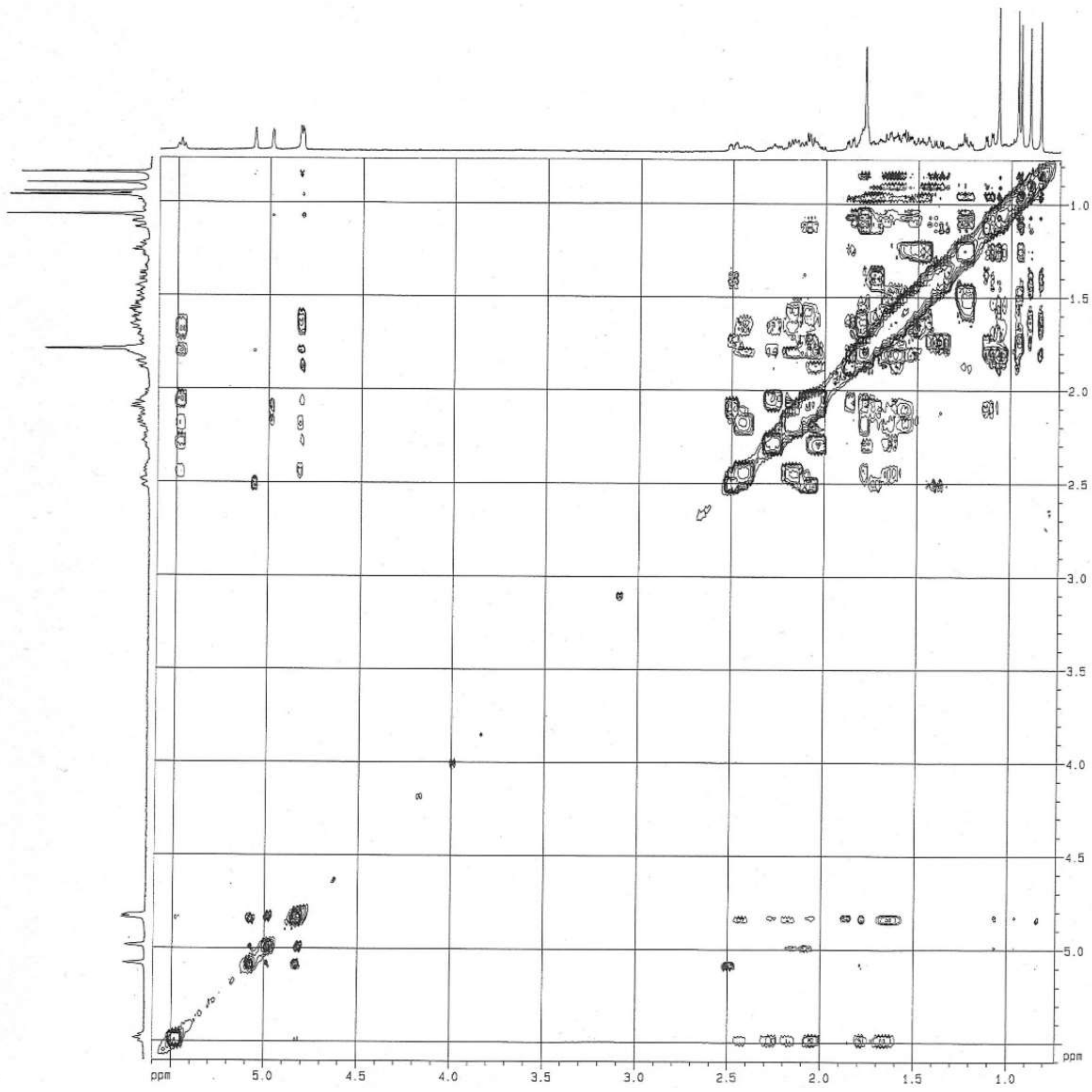

Figure S33. NOESY spectrum of **13** measured in C_6D_6

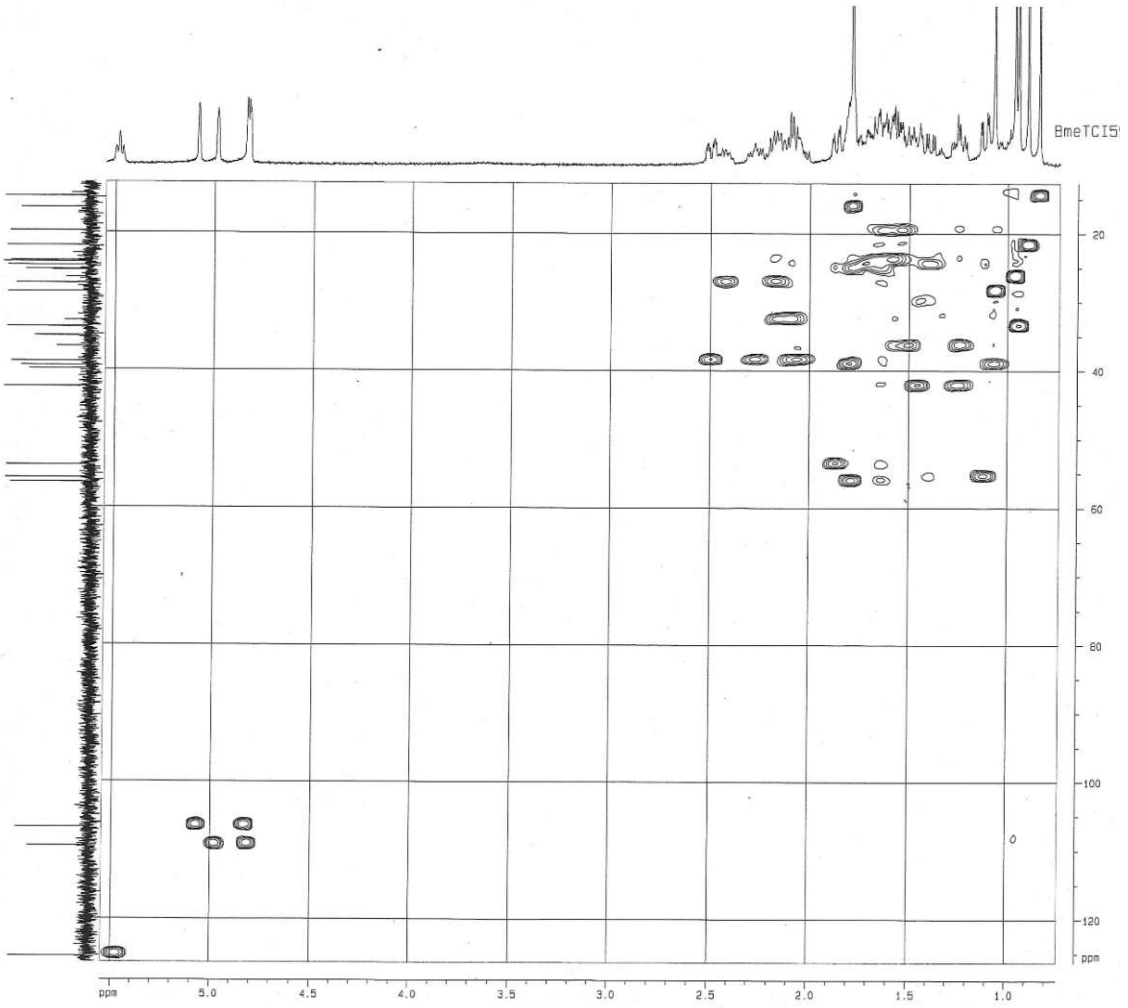

Figure S34. HSQC spectrum of **13** measured in C_6D_6

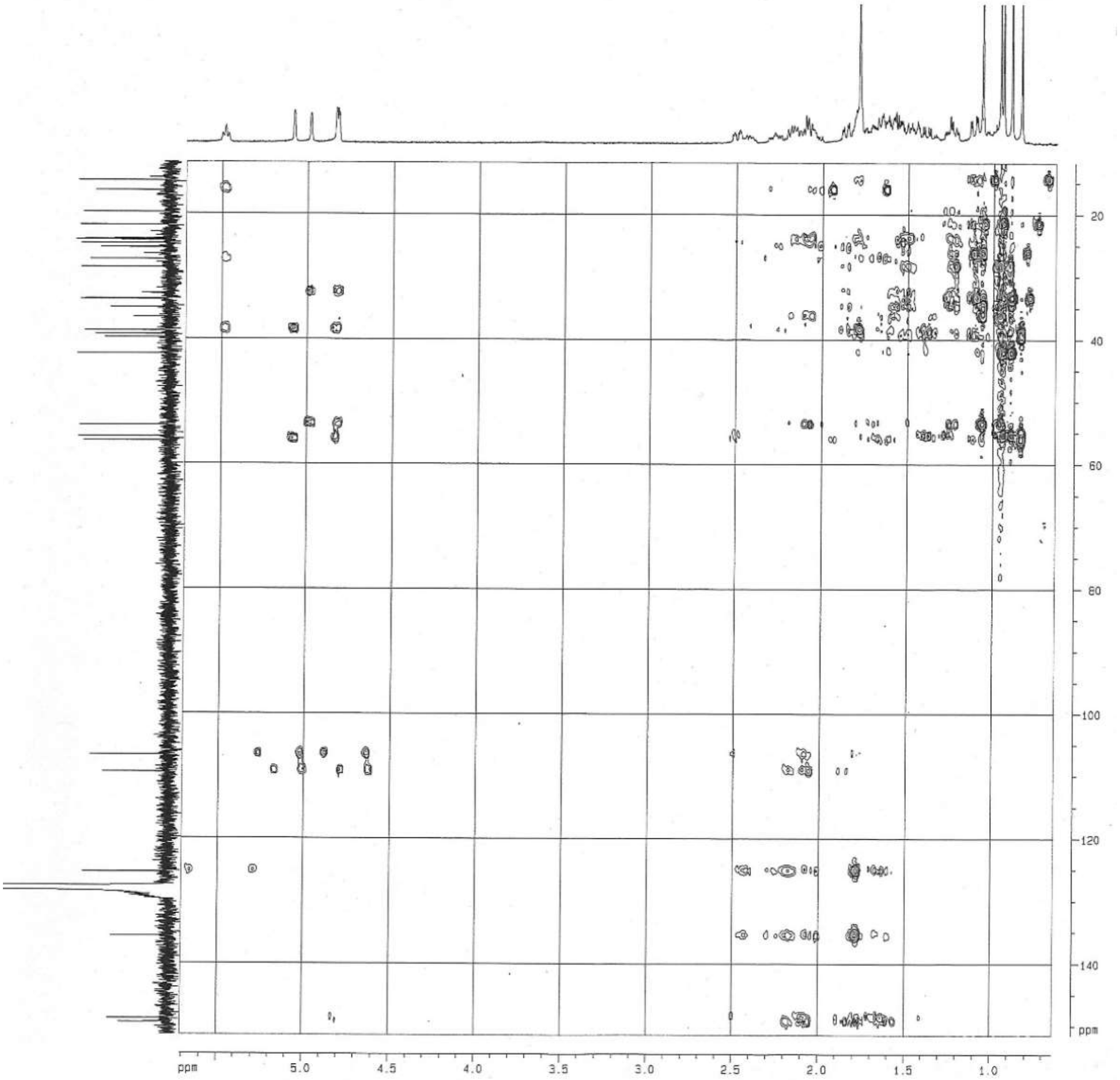

Figure S35. HMBC spectrum of **13** measured in C_6D_6

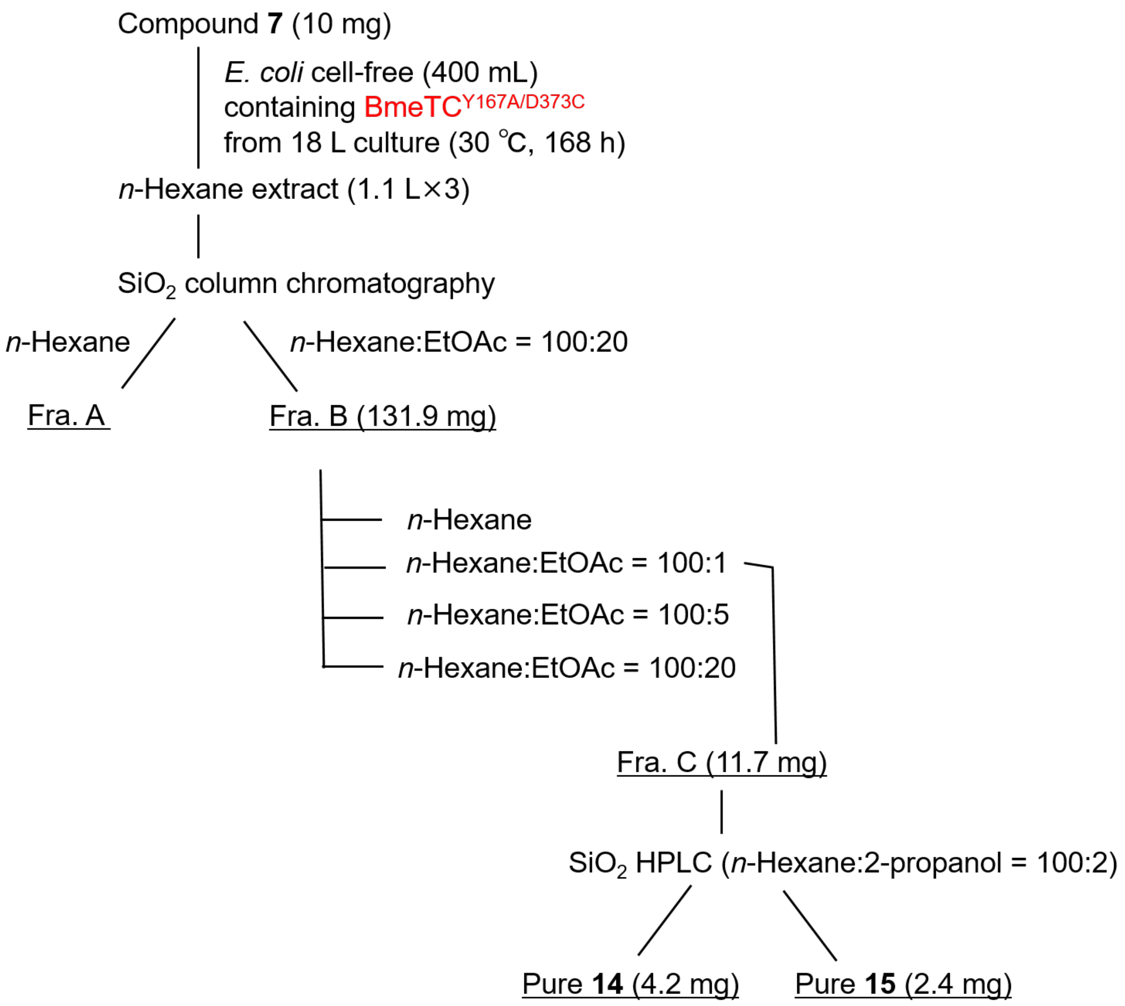

Fig. S36. Isolation of **14** and **15**

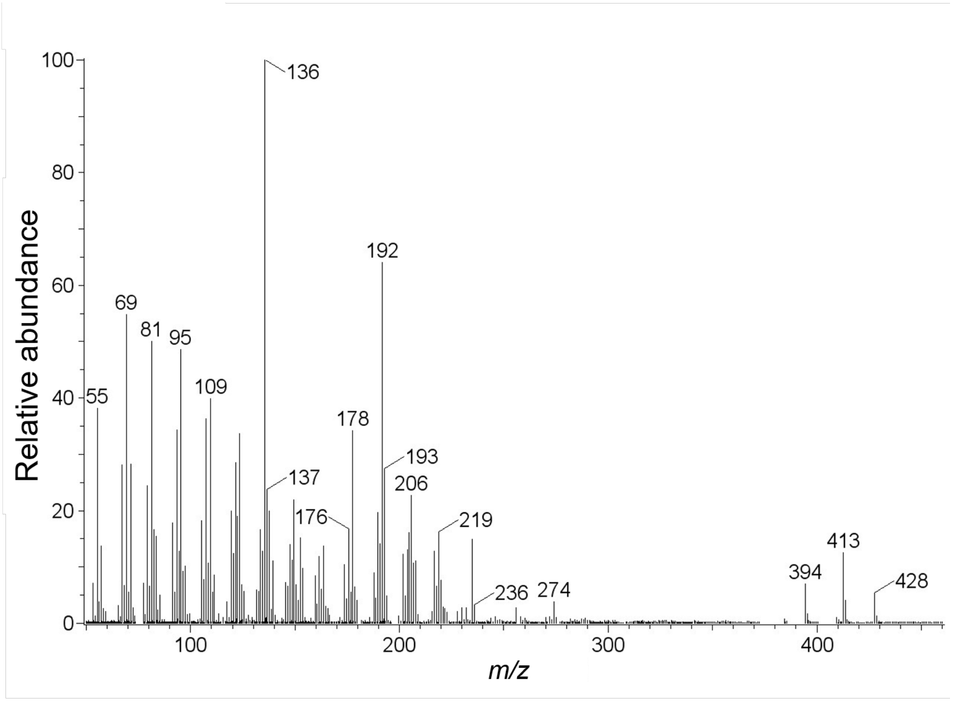

Figure S37. Mass spectrum (EI) of the compound **14**

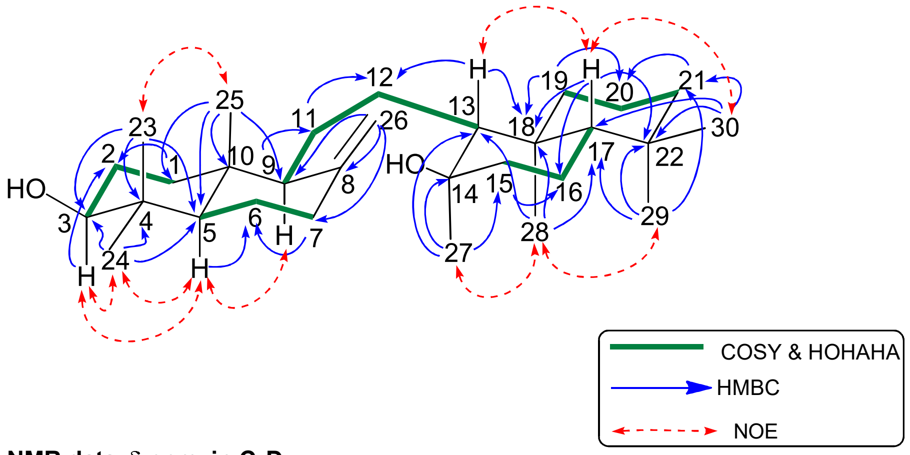

NMR data, δ ppm, in C_6D_6

NO.	^1H	^{13}C	NO.	^1H	^{13}C	NO.	^1H	^{13}C	NO.	^1H	^{13}C
1	1.02 (m); 1.74 (m)	37.4 (t)	9	1.50 (m)	58.3 (d)	17	0.80 (m)	56.3 (d)	25	0.77 (s)	14.9 (q)
2	1.74 (m)	28.5 (t)	10	——	39.3 (s)	18	——	39.5 (s)	26	5.07 (s); 5.16 (s)	107.9 (t)
3	3.06 (1H, t, 8.1 Hz)	78.5 (d)	11	1.66 (m)	28.3 (t)	19	0.93 (m); 1.64 (m)	39.8 (t)	27	1.04 (s)	24.3 (q)
4	——	39.1(s)	12	0.95 (m); 1.84 (m)	25.5 (t)	20	1.56 (m); 1.64 (m)	18.9 (t)	28	0.70 (s)	15.6 (q)
5	0.93 (m)	54.9 (d)	13	0.92 (m)	62.6 (d)	21	1.14 (m); 1.34 (m)	42.4 (t)	29	0.76 (s)	21.7 (q)
6	1.34 (m); 1.62 (m)	24.3 (t)	14	——	73.8 (s)	22	——	33.3 (s)	30	0.86 (s)	33.6 (q)
7	1.99 (m); 2.42 (m)	38.7 (t)	15	1.25 (m); 1.74 (m)	45.1 (t)	23	0.79 (s)	15.7 (q)			
8	——	148.1 (s)	16	1.08 (m); 1.48 (m)	20.9 (t)	24	1.00 (s)	28.5 (q)			

benzene: 7.15 ppm (^{1}H); 128.0 ppm (^{13}C)

Figure S38. NMR assignment of compound **14** measured in C_6D_6.

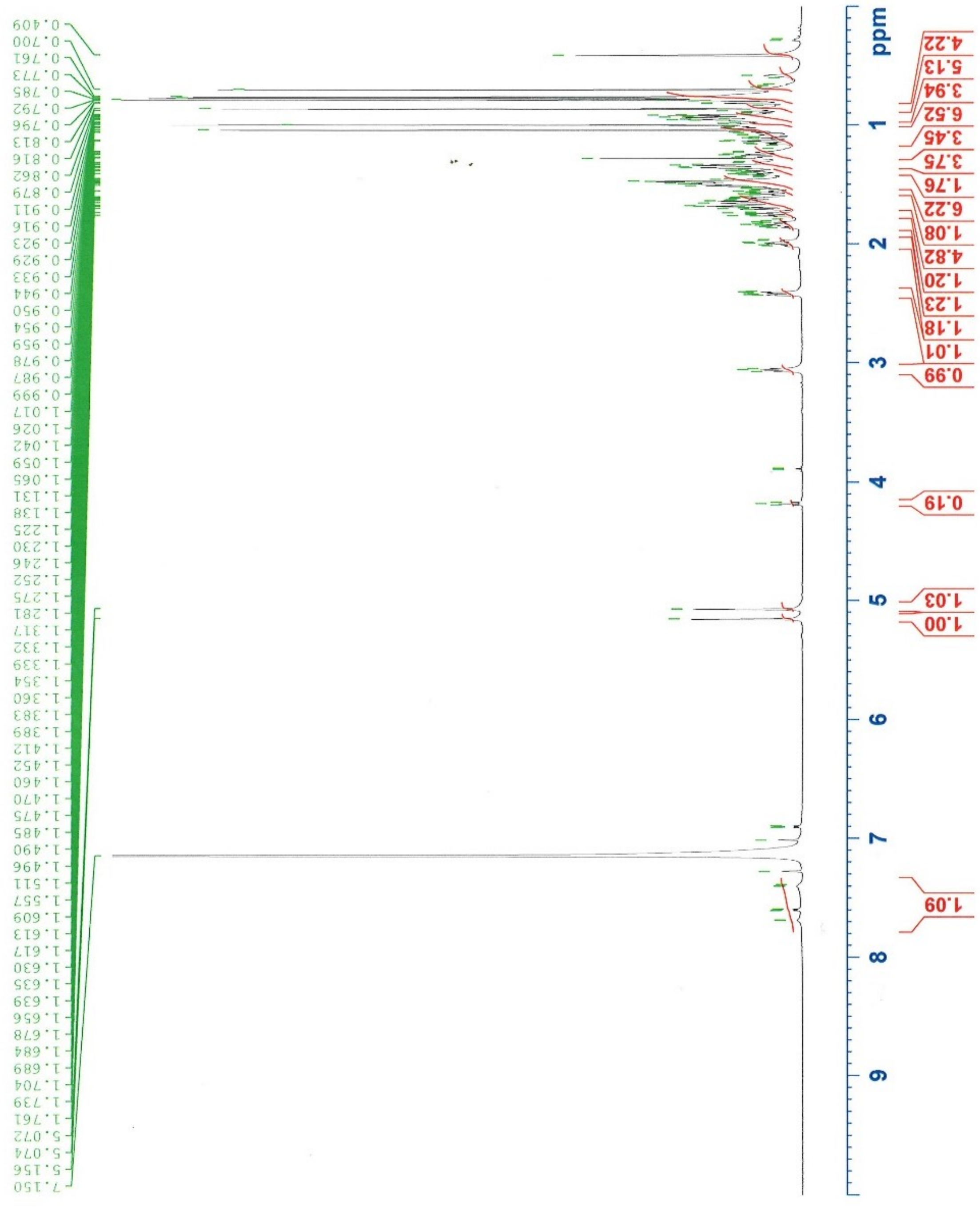

Figure S39. ^{1}H NMR spectrum of **14** measured in C_6D_6

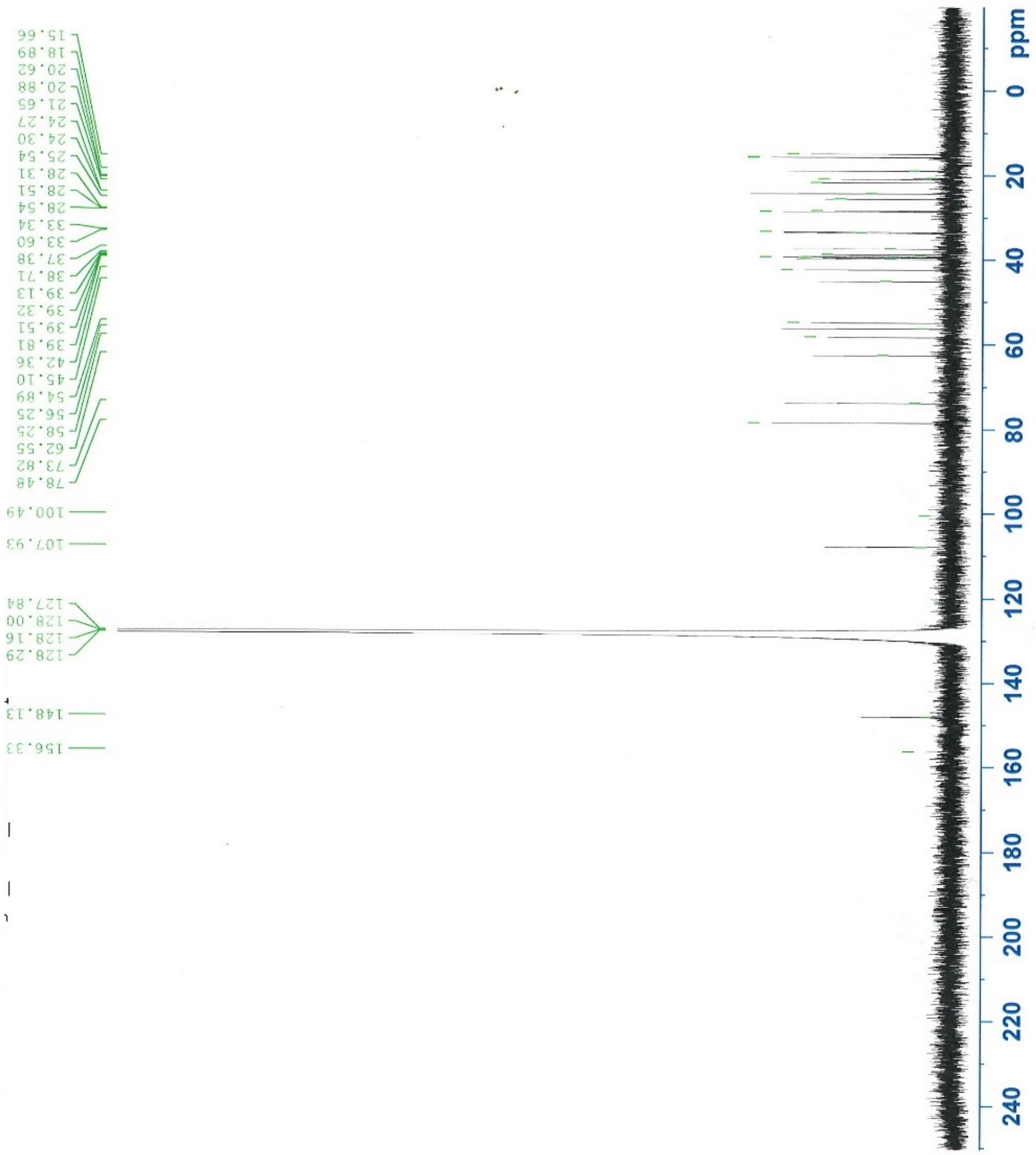

Figure S40. ^{13}C NMR spectrum of **14** measured in C_6D_6

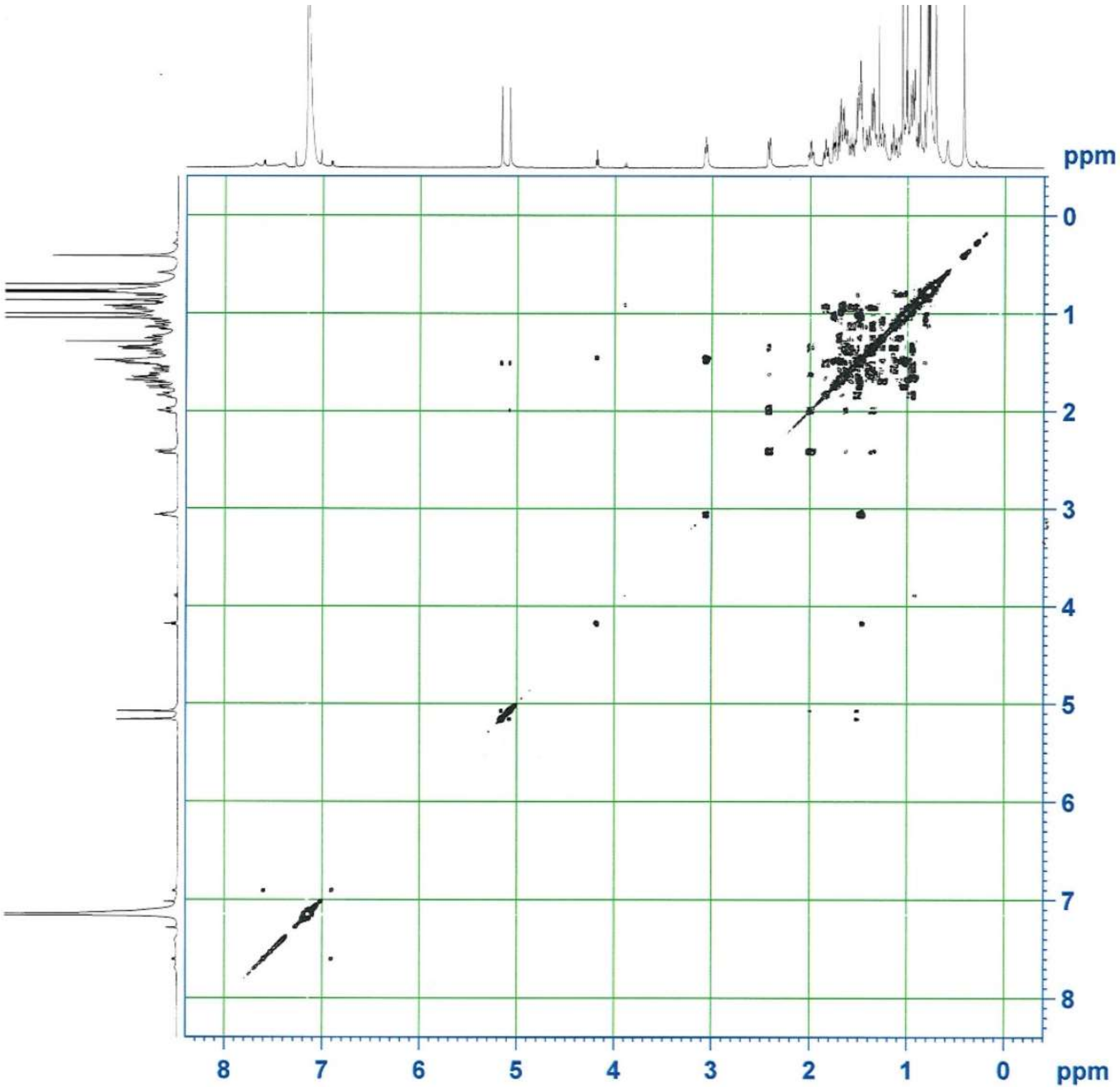

Figure S41. ^{1}H-^{1}H COSY spectrum of **14** measured in C_6D_6

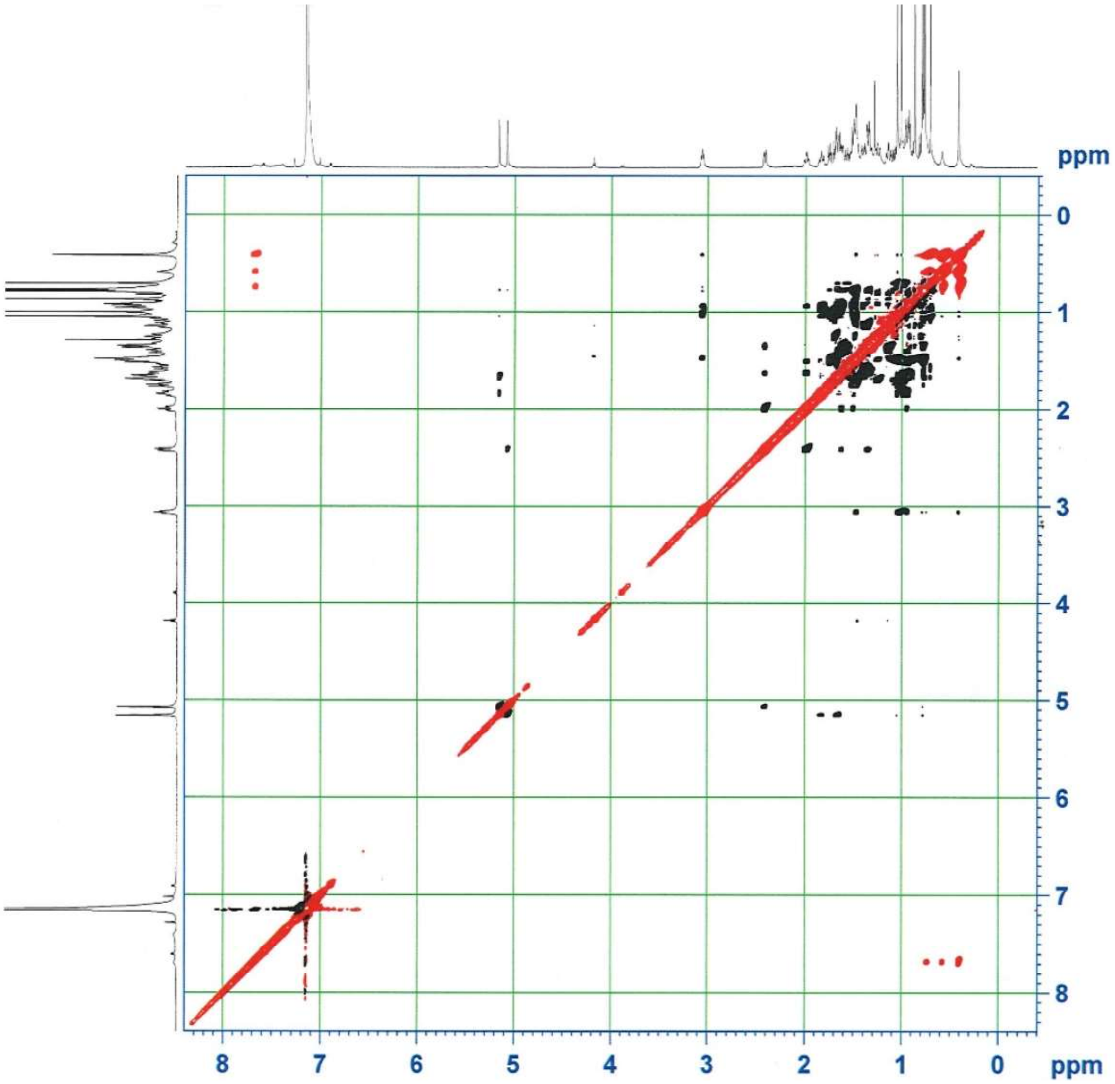

Figure S42. NOESY spectrum of **14** measured in C_6D_6

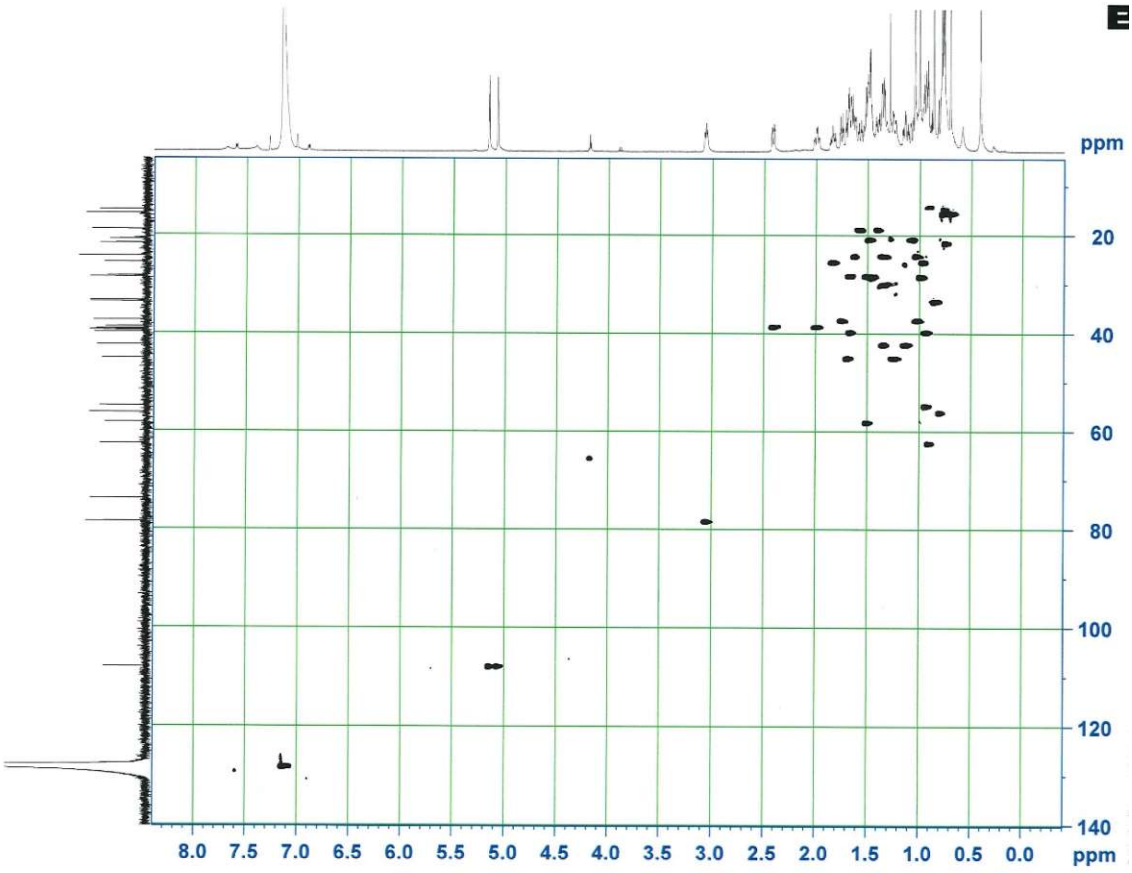

Figure S43. HSQC spectrum of **14** measured in C_6D_6

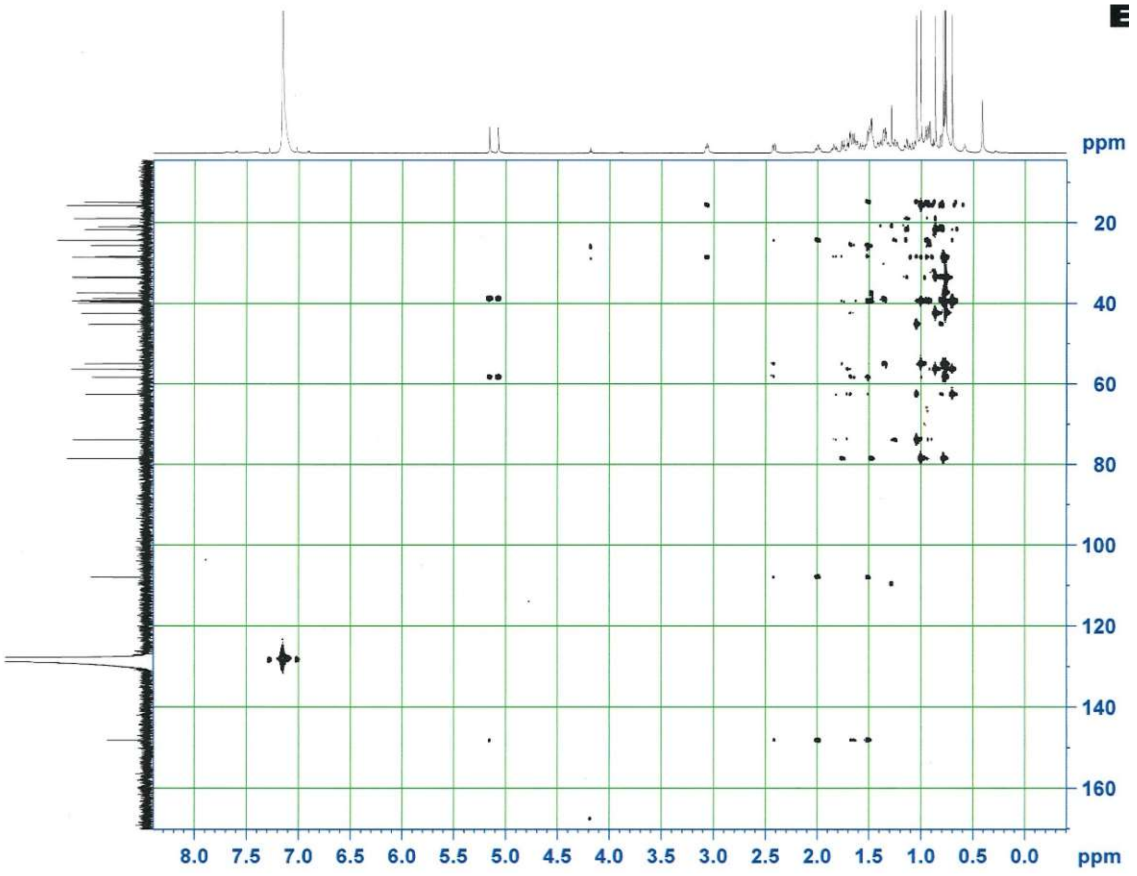

Figure S44. HMBC spectrum of **14** measured in C_6D_6

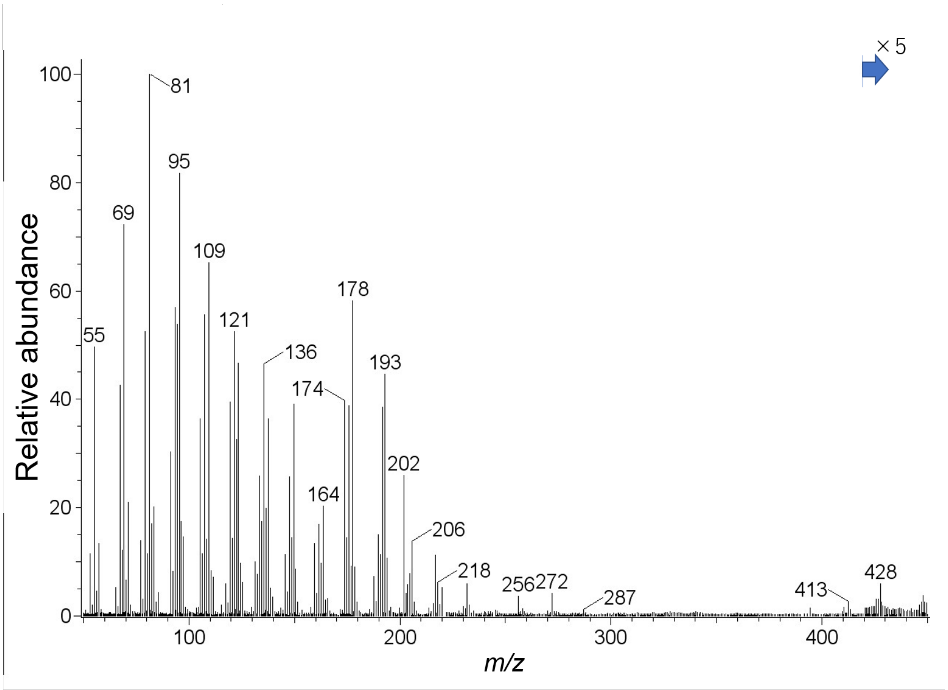

Figure S45. Mass spectrum (EI) of the compound **15**

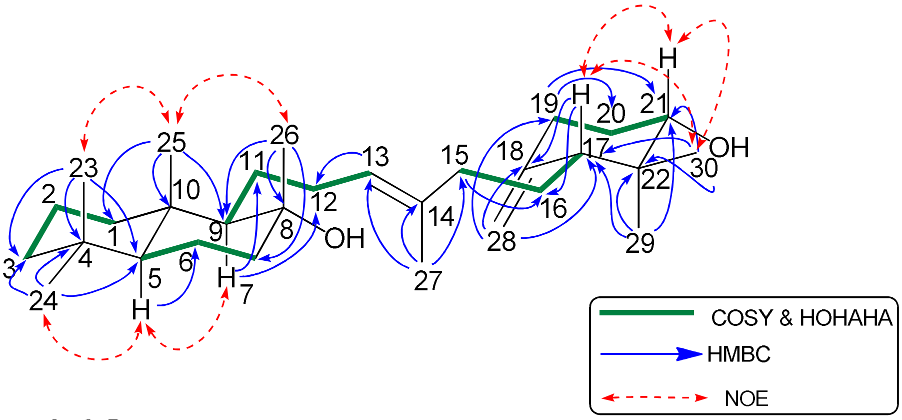

NMR data, δ ppm, in C_6D_6

NO.	^{1}H	^{13}C	NO.	^{1}H	^{13}C	NO.	^{1}H	^{13}C	NO.	^{1}H	^{13}C
1	0.91 (m); 1.61(m)	39.9 (t)	9	0.98 (1H, t, 3.9 Hz)	61.5 (d)	17	1.66 (m)	51.7(d)	25	0.67 (3H, s)	15.6 (q)
2	1.35 (m); 1.52 (m)	18.8 (t)	10	——	39.2 (s)	18	——	148.0 (s)	26	1.04 (3H, s)	24.1 (q)
3	1.10 (m); 1.33 (m)	42.3 (t)	11	1.32 (m); 1.60 (m)	26.1 (t)	19	1.36 (m); 1.60 (m)	32.6 (t)	27	1.75 (3H, s)	16.5 (q)
4	——	33.3 (s)	12	2.20 (m); 2.31 (m)	32.1 (t)	20	1.22 (m)	31.9 (t)	28	4.73 (s); 4.91 (s)	108.6 (t)
5	0.77 (m)	56.1 (d)	13	5.46 (1H, t, 6.9 Hz)	125.8 (d)	21	3.13 (1H, dd, 9.6, 4.2 Hz)	76.8 (t)	29	0.79 (3H, s)	16.5 (q)
6	1.06 (m); 1.46 (m)	20.8 (t)	14	——	135.4 (s)	22	——	40.6 (s)	30	1.02 (3H, s)	26.3 (q)
7	1.26 (m); 1.74 (m)	44.9 (t)	15	1.98 (m); 2.30 (m)	39.2 (t)	23	0.74 (3H, s)	21.7 (q)			
8	——	73.6 (s)	16	1.76 (m)	24.5 (t)	24	0.84 (3H, s)	33.5 (q)			

benzene: 7.15 ppm (^{1}H); 128.0 ppm (^{13}C)

Figure S46. NMR assignment of compound **15** measured in C_6D_6.

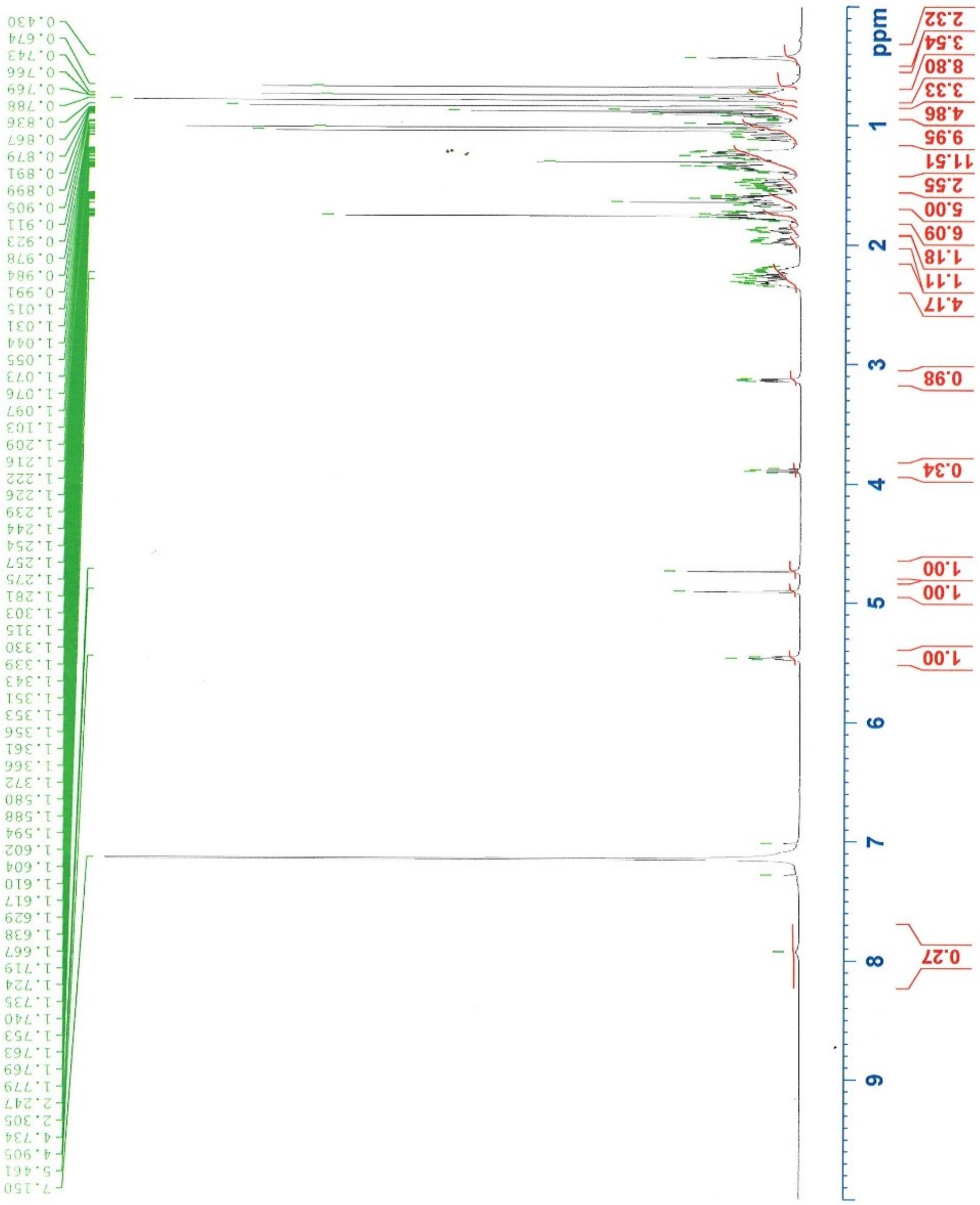

Figure S47. ^{1}H NMR spectrum of **15** measured in C_6D_6

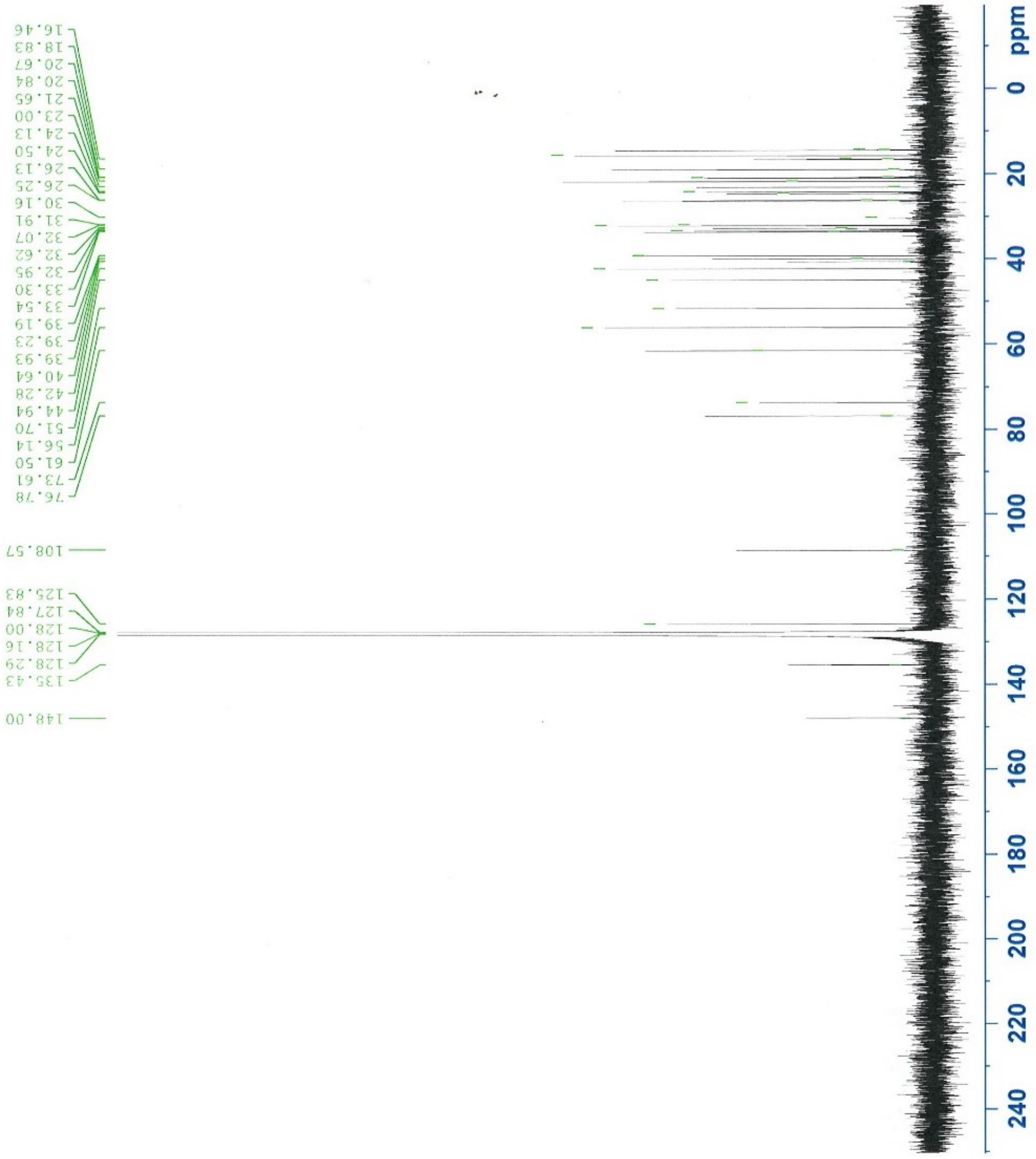

Figure S48. ^{13}C NMR spectrum of **15** measured in C_6D_6

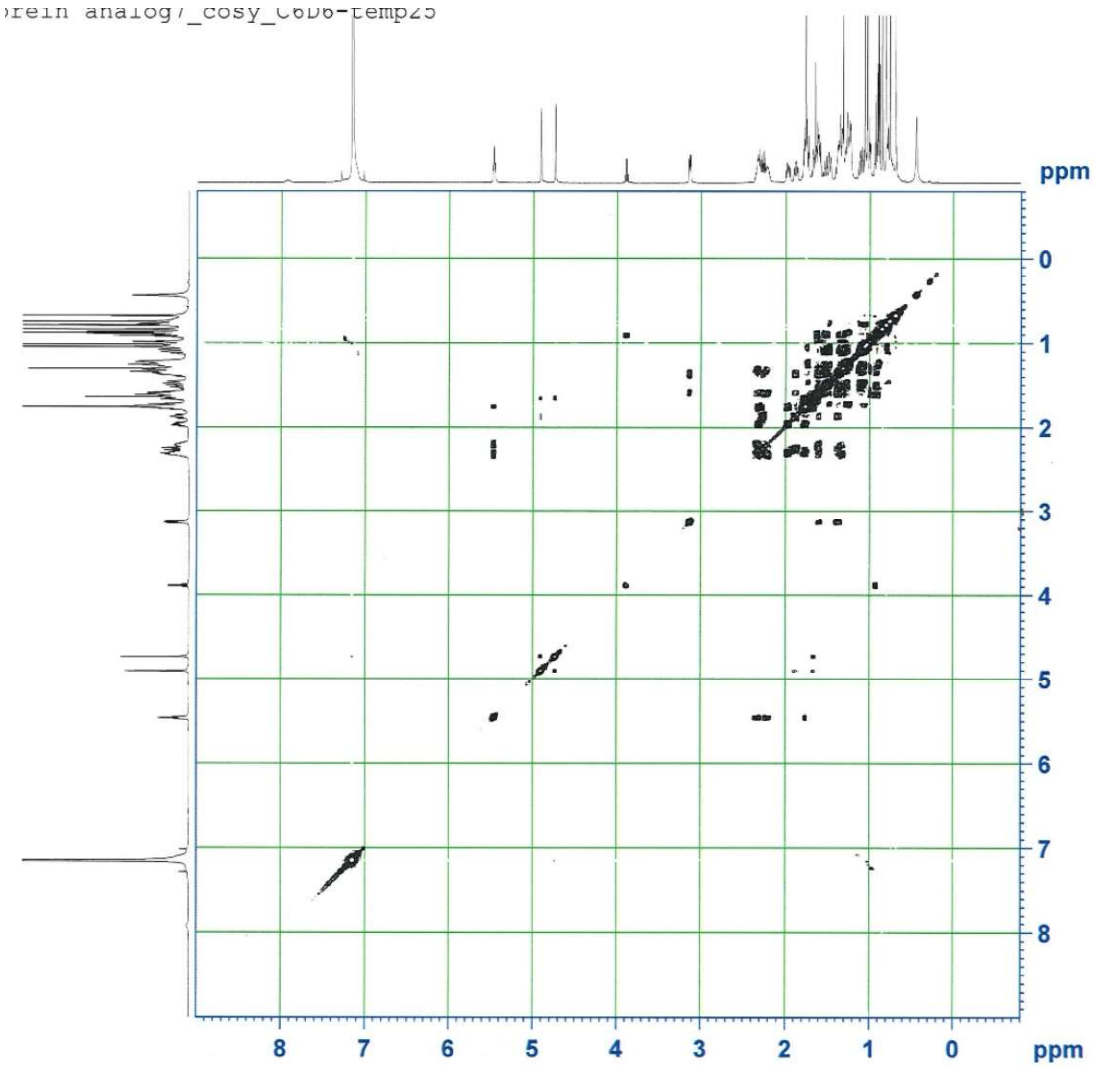

F

igure S49. ^{1}H-^{1}H COSY spectrum of **15** measured in C_6D_6

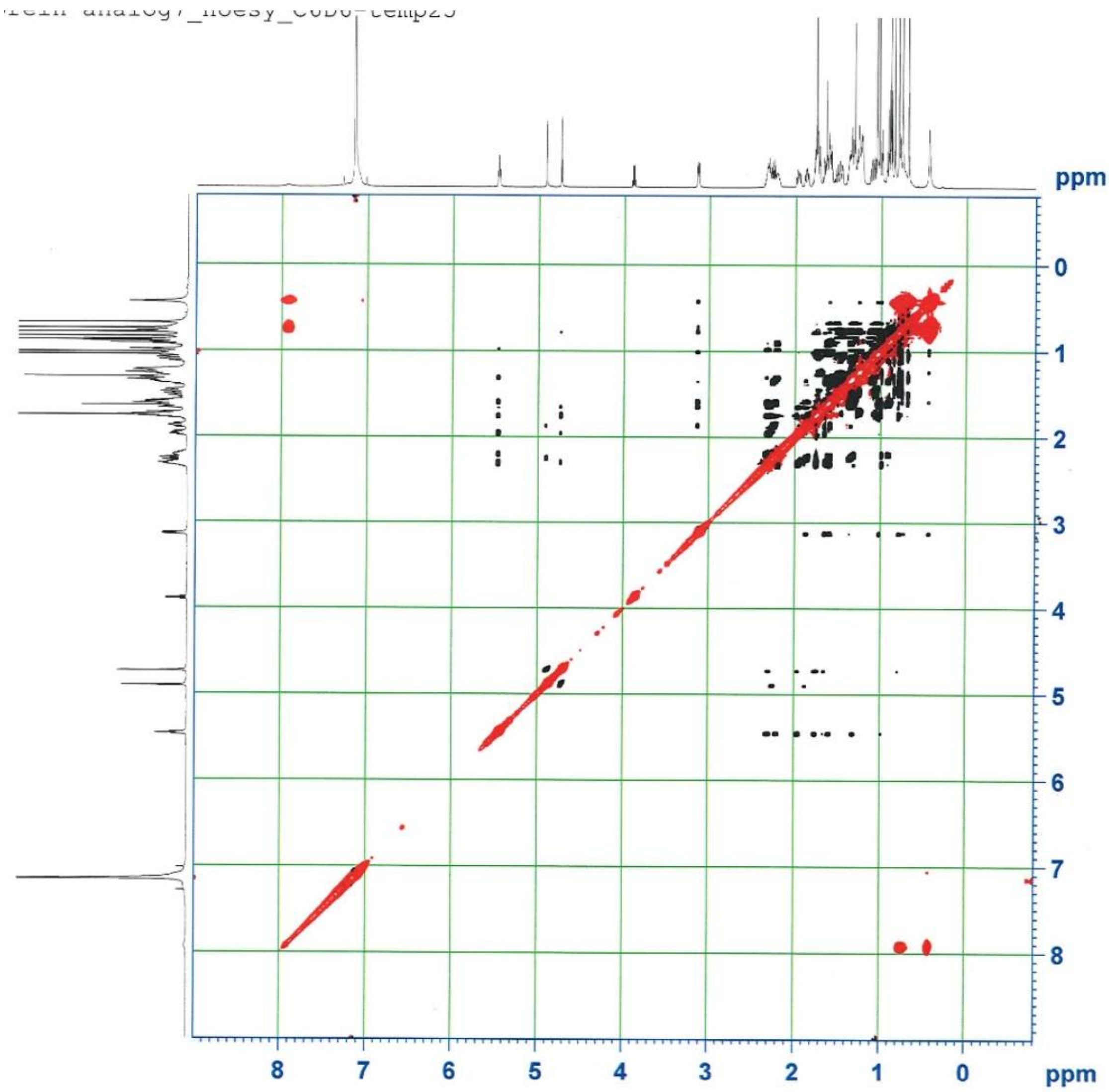

Figure S50. NOESY spectrum of **15** measured in C_6D_6

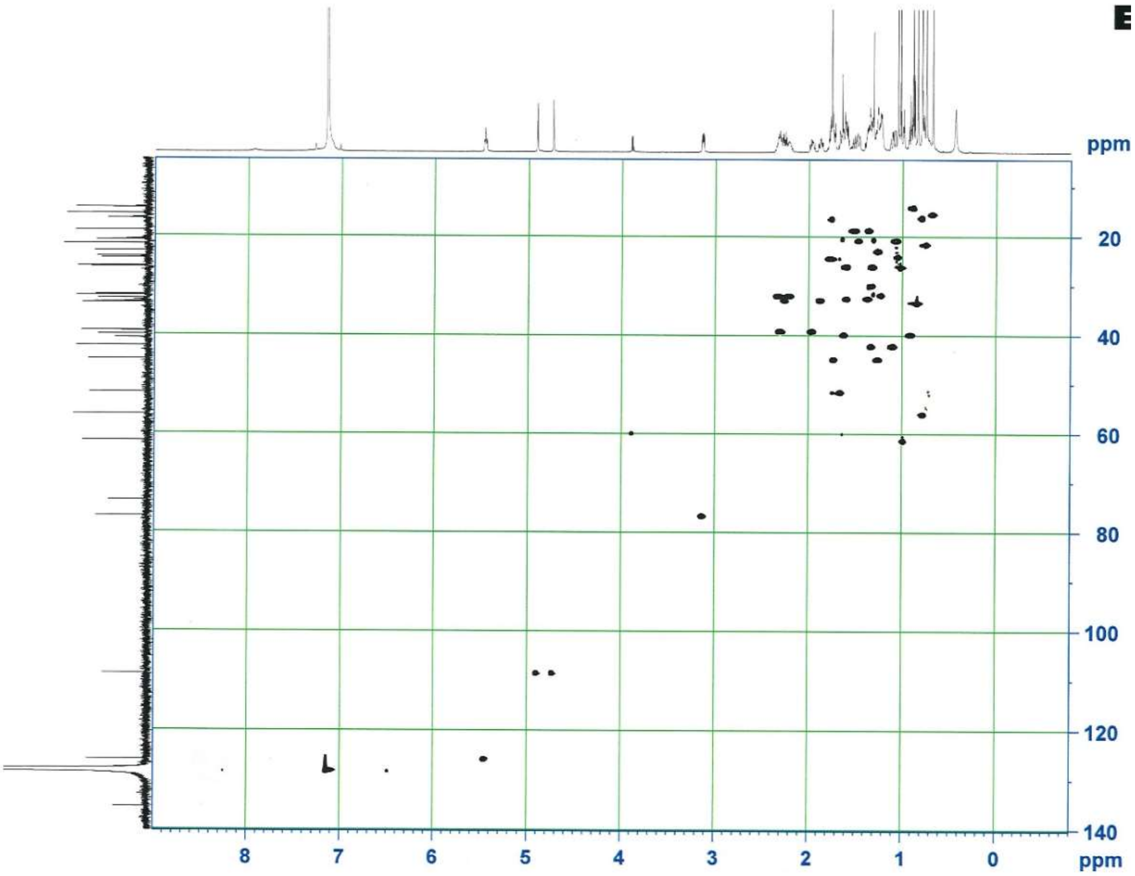

Figure S51. HSQC spectrum of **15** measured in C_6D_6

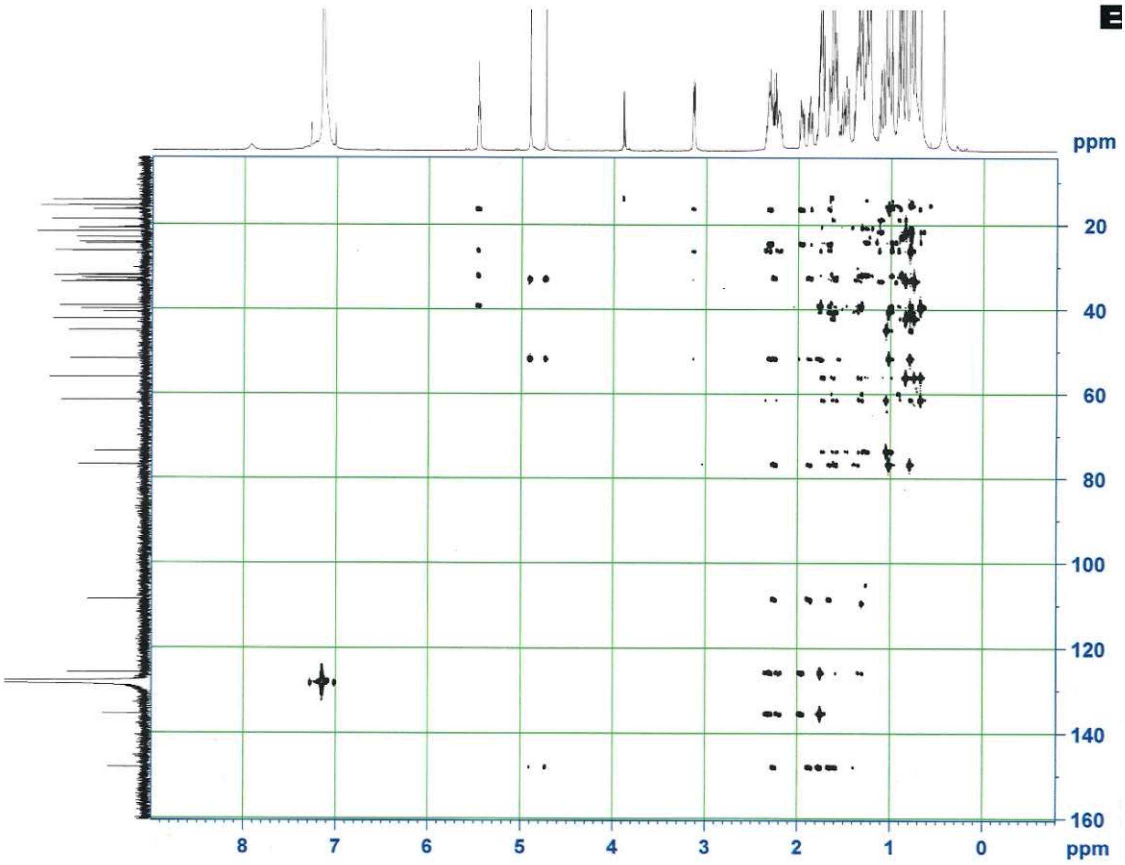

Figure S52. HMBC spectrum of **15** measured in C_6D_6

2. Gas chromatogram of reaction products and residual substrate.

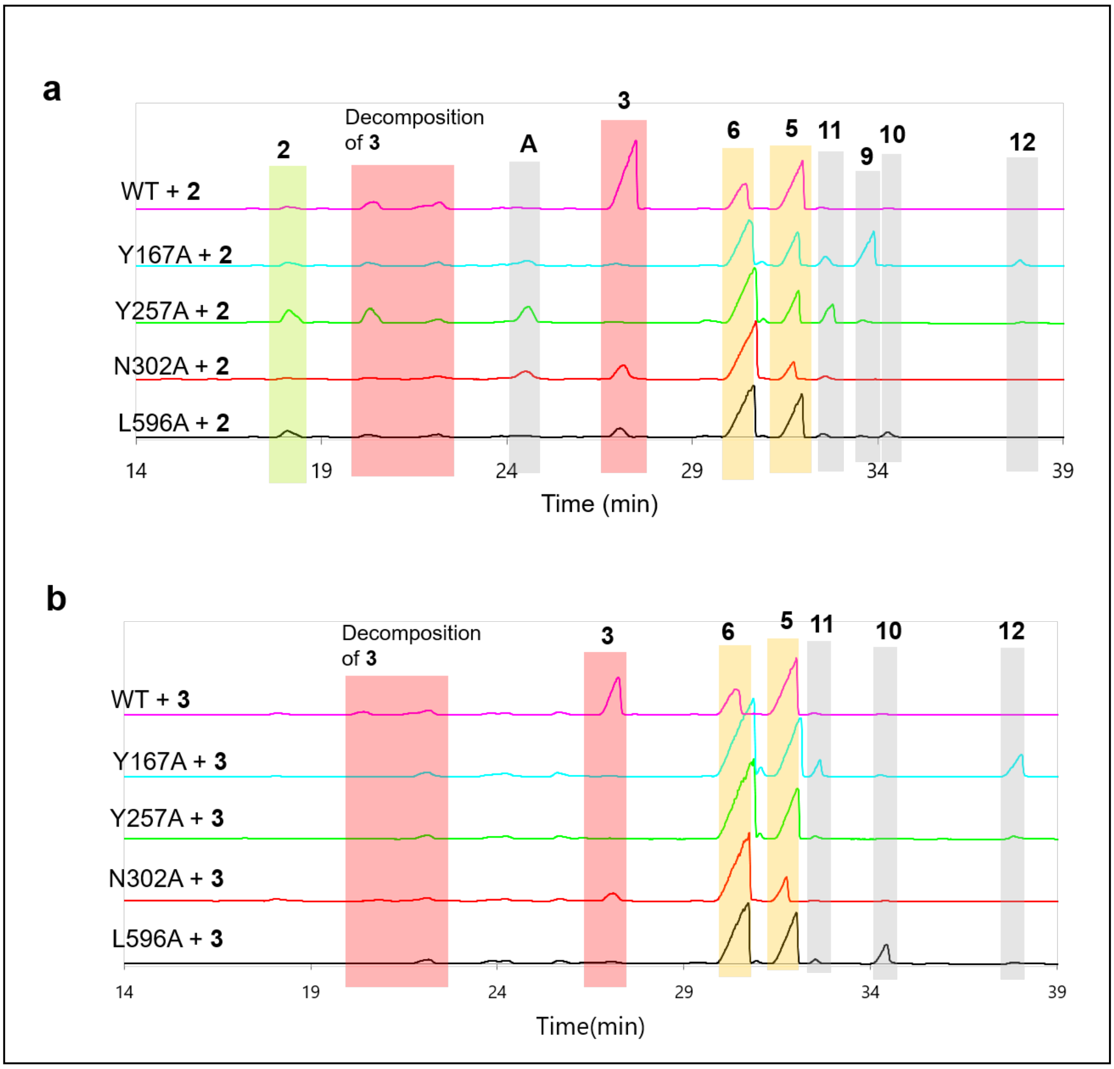

Figure S53. Gas chromatogram of reaction products and residual substrate.

(a) Substrate: **2**. **(b)** Substrate: **3**. These results showed that **9** is converted directly from **2**, whereas **10-12** is produced from **2** via **3**.

Although the product profiles had been analyzed in the previous study [5], unknown compounds **9-12** were identified in this study.

3. Vitamin D binding affinities of vitamin Ds and triterpenes

Table S1. Vitamin D binding affinities of vitamin Ds and triterpenes.

	**Concentration at 50% maximum light intensity (μM)**	**Relative binding affinity**
**VD-III (1,25$(OH)_2D_3$)**	0.00064 ±0.00022	176000
**VD-II (25$(OH)D_3$)**	0.074±0.015	1510
**VD-I (Vitamin D_3)**	10.5 ± 2.2	10.7
**1**	112 ± 29	1
**3**	128 ± 38	0.88
**4**	n.d.	-
**6**	11.1 ± 1.5	10
**10**	24.6 ± 8.2	4.6
**11**	12.6 ± 2.1	8.9
**13**	16.0 ± 2.2	7.0
**14**	13.9 ± 2.5	7.1
**15**	45.2 ± 17.1	2.5

The concentration at which relative intensity reached 50% of the maximum value for **4** could not be estimated due to its very low VDR binding affinity (relative intensity at a concentration of 100 μM for **4** was 0.82 ± 0.07). Data are presented as the mean ± SD of at least triplicate determinations.

4. Binding energies of native and synthetic ligands in the complex structures of VDR.

Table S2. Binding energies of native and synthetic ligands in the complex structures of VDR.

Ligands	Binding energies (kcal/mol)
**VD3**	-195.333
**1**	-121.863
**6**	-126.609
